# Allergies, asthma treatment, and eviction diet have a significant impact on the respiratory effort during sleep and the apnea-hypopnea index in children with obstructive sleep apnea-obesity/asthma association: A STROBE-compliant study

**DOI:** 10.1097/MD.0000000000041730

**Published:** 2026-02-13

**Authors:** Kalomoira Kefala, Philippe Guerin

**Affiliations:** a Cabinet d’allergologie de l’enfant et de l’adolescent, Paris, France; b CSMD Clavel, Foundation «Œuvre de la Croix-Saint-Simon», Paris, France; c Clinique Lambert, Ramsay Générale de Santé, Colombes, France (at the time of the study); d Breath Clinic, Les Clarines, Riom-ès-Montagnes, France.

**Keywords:** allergy, apnea-hypopnea index, asthma treatment, eviction diet, obesity, obstructive sleep apnea, polysomnography, respiratory effort during sleep, respiratory polygraphy, sleep-disordered breathing

## Abstract

The pathophysiological mechanisms implicated in obstructive sleep apnea (OSA) associated with asthma/allergies remain unclear. Apnea-hypopnea index (AHI) alone is insufficient to accurately guide adequate treatment without identifying the specific patient profile. The same recommendations apply for children suffering from sleep-disordered breathing/asthma either OSA. Asthma is considered favoring OSA; however, it is unclear if allergies preexist predisposing patients to OSA. OSA correlates to obesity; however, links between obesity, allergy, and OSA remain unexplored. Obesity is considered a risk factor for OSA. Nevertheless, children with OSA increase their body mass index (BMI) despite adequate sleep apnea treatment and adapted weight interventions. We aimed to study the respiratory polygraphy (PG)/polysomnography (PSG) profile of children with OSA-asthma association and the influence of allergies and asthma treatment (AT)/eviction diet (ED) on the AHI/respiratory effort/BMI to diagnose, treat, and prevent pediatric OSA-asthma and obesity-associated early and accurately. We effectuated a combined STROBE-compliant study with a cross-sectional/case control/diagnostic part and a cohort. We used Statistical Package for the Social Sciences and path analysis (analysis of a moment structure). We evaluated the effects of AT, allergies, and allergen eviction on PG/PSG parameters such as the AHI, the respiratory effort, the BMI, the respiratory distress index, the sleep fragmentation, oxygen desaturation index, and sleep fragmentation ventilatory origin. We identified that AT, ED, and the coexistence of non-IgE-mediated and respiratory allergies influenced the AHI, respiratory effort during sleep, and the BMI. Increased respiratory effort during sleep innately correlates with sleep-disordered breathing/OSA related to allergies, especially the coexistence of respiratory and non-IgE mediated allergies, and is on the origin of the sleep fragmentation of children suffering OSA-asthma/associated, even if AHI remains in low levels; decreases (as AHI) with AT or ED, and if untreated, contributes to AHI increase, thus favoring the persistence of OSA and its comorbidities (hyperactivity, decrease in school performance, behavior/concentration problems) asthma and obesity. Consideration of AT, allergies, and ED upon interpretation of PG/PSG parameters could ameliorate the diagnosis and treatment of OSA-asthma-associated and possibly avoid, upon their origin, asthma, and obesity.

## Graphical abstract

**Figure:**
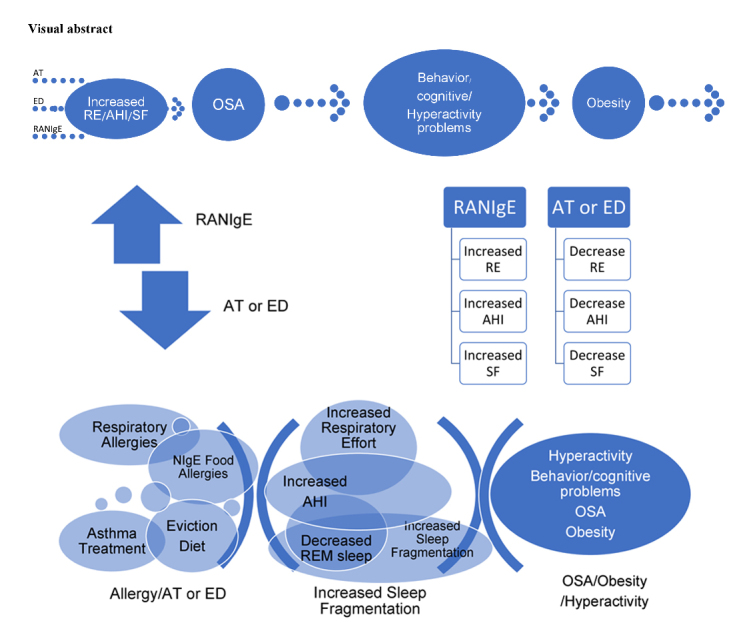

**Key points**

- A significant number of children suffering from OSA do not ameliorate with current treatments.
- AHI cannot sufficiently identify OSA and distinguish between obstructive and nonobstructive OSA/SDB.
- Many children with OSA continue to increase their BMI under continuous positive airway pressure.
- Allergic children suffer a significant SF non-explained by associated respiratory events.
- RE during sleep increases in allergic children.
- SF/increased RE contribute to BMI increase.
- Allergies use sleep disorders/SF/RE increase as mediators for its consequences (cognitive/behavioral disorders/asthma/obesity).
- SF should alert even if AHI is in low values.
- Increased RE should alert for allergy/asthma/obesity.
- Allergy diagnosis and treatment in preschool children should be a priority in public health policies to avoid sleep disorders, OSA and its consequences, asthma, and obesity.

## 1. Introduction

Adequate treatment, favorable factors, and pediatric (ped)-obstructive sleep apnea (OSA)-asthma-allergy-obesity subgroups in mild/severe pedOSA-asthma, along with the effects of treatment on apnea-hypopnea index (AHI)/respiratory effort (RE)/body mass index (BMI), remain unexplored.

Obstruction induced by allergic inflammation cannot be explained only by AHI; apnea in obstructive OSA is mainly retro lingual.^[1]^ Rhinitis usually refers to viral infections that cause confusion among the parents.

The current recommendations exist for children with obstructive OSA (premature babies and congenital malformations). Nonobstructive asthma/allergy-associated OSA appears to be a distinct entity. However, we applied the recommendations for obstructive OSA in children with OSA-allergic asthma, with inconsistent efficacy.

Th1 inflammation has been implicated in obesity.^[2]^ Obese children with normal spirometry results (case 4, Online Repository Material, Supplemental Digital Content, http://links.lww.com/MD/P846) did not follow AT. Nevertheless, the links between obesity, allergies, and OSA remain unclear with no tailored treatment(s). The efficacy of tonsillectomy and adenotonsillectomy (T&A) is lower in obese children than in nonobese children.^[3]^

In OSA-asthma assessment, nocturnal asthma symptoms are related to sleep fragmentation (SF). SF disrupts nocturnal hormonal secretion.^[4]^ Stress and hormone secretion increase appetite.^[5]^ Sleep deprivation is linked to obesity,^[6]^ altered metabolic rate, and increased hunger,^[7]^ leading to the failure of weight maintenance/weight loss interventions.^[4,8]^

Significant sleep splitting decreases rapid-eye-movement (REM) sleep and mentally restorative sleep^[9]^ (cases 5 and 6, Online Repository Material, Supplemental Digital Content, http://links.lww.com/MD/P846).^[10,11]^ A decrease in REM sleep correlates with obesity^[12]^ as it alters energy balance, increases food intake, and decreases energy use.^[13]^

The hypothesis that SF impairs growth hormone (GH) secretion and leads to growth stagnation does not explain why many allergic children: (a) have a normal BMI despite suffering severe persistent sleep-disordered breathing (SDB) and (b) become overweight/obese, which is compatible with the fact that sleep deprivation favors obesity.^[6]^

Impaired GH secretion following severe sleep deprivation. However, the lack of GH secretion at night may be decompensated during the day and its secretion is age-dependent.^[14]^

Stress and low blood sugar levels/nutrition influence GH release, which regulates carbohydrate and lipid metabolism^[15,16]^ rapid weight loss and/or malnutrition increases, whereas obesity decreases GH.^[17]^ Decreased GH levels may be related to a metabolic imbalance that mimics insulin resistance.^[16]^ Therefore, SF could provoke a decrease in GH levels, which could favor obesity through insulin resistance.

We investigated the respiratory polygraphy (PG)/polysomnography (PSG) and clinical profile of allergic children suffering from OSA-asthma along with the effect of treatments and concomitant allergies on AHI/RE/BMI to (a) supply personalized treatment/recommendations and (b) explore the pathophysiology of pedOSA-asthma-associated.

We distinguished the following: (a) adequate treatment differs in SDB/OSA-asthma/allergy-associated and obstructive-origin OSA^[11,18,19]^; (b) obesity could be a consequence of an inadequately treated OSA allergy^[20]^; (c) RE could help correctly identify the OSA subgroup and the evolution of obesity^[20–22]^; and (d) the evaluation of concurrent treatments (AT or eviction diet [ED]) and PG parameters (RE/SF) could help correctly interpret PG/PSG.^[20,21]^

## 2. Methods

Our study used a cross-sectional/case-control diagnostic part and cohort (acronym: TRUST IT ALL STUDY) to evaluate the origin of the SDB/OSA-asthma association and the effect of AT/ED on PG/PSG in 2 to 16-year-old children (N) who proceeded for allergology advice and concomitant SDB mostly spontaneously (parents’ initiative) either addressed by general practitioner (GPs)/specialists (otorhinolaryngologist [ENT]/dentists). We explored whether allergies accompany SDB before the establishment of severe asthma.

We consecutively recruited eligible children from January 2018 to August 2019 from 2 primary care centers and 1 outpatient clinic, incorporating children of various ethnicities and continents from a broader area around Paris and nearby towns. The primary care centers and the outpatient clinic were public and private clinic/offices. The private clinic/offices accepted patients from all social classes. Patients whom social insurance was totally covered from the government were also accepted. Thus, there were no bias upon selection of patients depended on their social class.

The patients decided whether they needed appointments after initial exploration and advice. As the classification was based on recruitment, the loss to follow-up was not problematic. Non-opposing content was obtained from the parents. The study was approved by the Institutional Review Board of the Conseil d’Orientation Scientifique Ramsay Générale de Santé Comité d’Ethique (No. IORG-IRB: IORG0009085, No. IRB: COS-RGDS-2018-06-030-Approval IRB-KEFALA-K).

No examinations were performed, in addition to those necessary for each patient. Therefore, we overcame the following selection bias: (a) patients with severe asthma, (b) social class, and (c) unnecessary exams/treatment.

We used a detailed questionnaire to collect clinical signs, domestic exposure, personal/family history, and demographic data. Percentages were calculated on the basis of the number of answered questionnaires. Many parents could leave unanswered questions which they considered not bothersome; we record them as missing. Moreover, the parents recorded more clinical signs than those initially included in the questionnaire.

Performed at home:

- PG: (a) Cidelec LX (tracheal sound sensor) 24 N; (b) somnolter (jawac, recorded mandibular movements) 50 N.
- PSG (Cidelec LXe) on consecutive days if PG was inconclusive: 3 N.

AHI was differentiated between the supine and non-supine positions. We verified that sleeping was avoided in the supine position, particularly in adolescents and children with obesity.^[23]^

Clinical information and reference standards (normal: AHI ≤ 1/hour total sleep time (min) [TST]; 1 < AHI ≤ 5: mild OSA; 5 < AHI ≤ 10: moderate OSA; AHI > 10/hour TST: severe OSA^[24]^) were available to PG/PSG performers/readers. We followed AHI/OSA diagnosis and treatment guidelines.^[24,25]^

We recorded AT/ED/ENT exam/T&A/spirometry for suspected asthma, post-effort for exercise-induced asthma, complete blood count, skin prick tests, and specific IgE for common aeroallergens; IgE-mediated food allergy (IgEFA) for clinical signs; and patch tests (PT) for milk/wheat ± soy/other for digestive signs/eczema/ED. PT was evaluated according to the International Contact Dermatitis Research Group criteria.^[26]^ Non-IgE-mediated food allergy (NIgEFA) was diagnosed as positive PT with clinical amelioration after 2 months of ED.

Somnolter only: RE, SF, sleep fragmentation ventilatory origin (SFVO), respiratory distress index (RDI).

PSG only: micro-arousals, intra-sleep arousals > 30 seconds.

The patients who were not under AT/ED either had no allergy, nor obesity/overweight were used as controls in the relative groups.

To evaluate influence of treatments (AT/ED) and allergies on AHI/RE, we grouped: AT or ED: asthma treatment or eviction diet (ATED), respiratory allergies (RA) + NIgE: RANIgE, mite allergy (MA) + NIgE: MANIGE.

We created a combined (dummy) variable (ATED.RANIgE) to represent both the treatment (AT or ED) and the coexistence of RANIgE: ATED + RANIgE: ATED.RANIgE.

RE/AHI categorized: RE > 20%, RE > 28%, AHI > 6.8 n/h.

The effect of combined treatments/allergies on AHI/RE increased/decreased more as compared to the separate effect of AT/ED and RA/NIgE.

We report the results with the group (AT or allergen eviction) and/or RANIgE that the effect in decreasing/increasing AHI/RE was more significant and thus, more clinically applicable.

Dichotomous variables (BMI adjusted age/sex).

For the comparisons concerning obesity, we created 2 dichotomous variables through creating Groups 0 and 1 (BMI adjusted for age/sex):

(1) “obesity group” (obesity/overw vs HW):
  Group 0 consisted of healthy weight children (BMI 5th–85th percentile) whereas Group 1 of both overweight (BMI > 85th percentile and <95th percentile) and obese (BMI ≥ 95th percentile) children.
  Group (G) 0: Healthy weight (HW) (BMI 5th–85th percentile).
  G1: overweight (abbrev: overw) (BMI > 85th to <95^th^ percentile) + obesity (BMI ≥ 95th percentile).
(2) “obesity versus non-obesity” (HWoverw/ vs obesity):
  We grouped healthy weight (BMI 5th-85th percentile) and overweight children (BMI > 85th percentile and <95th percentile) as Group 0 whereas children suffering obesity (BMI ≥ 95th percentile) as Group 1.
  G0: HW + overw.
  G1: obesity.

Outcomes: obesity, obesity/overweight, AHI, RE, AHI > 6.8, RE > 20, and RE > 28.

Exposures: AT, ED, ATED, A, NIgE, RA, RANIgE, obesity, and obesity/overw.

Potential confounders or effect modifiers: ATED, RANIgE, RE, obesity, and overweight.

ATED, RANIgE, obesity/overweight were taken into consideration as confounders (when the one among them was measured) and adjusted to identify the causality factors and the real effect of each factor.

Exploratory factor analysis and principal component analysis revealed 5 principal variables: ATED, RANIgE, AHI, RE, and BMI.

Principal component analysis/exploratory factor analysis identified RE as a factor independent of AHI. The remaining PG parameters (RDI/oxygen desaturation index/SF/SFVO) related to AHI were all related to obstruction.

We used Statistical Package for the Social Sciences to evaluate percentages, PG/PSG parameters, correlations, *t*-test, effect sizes (eta, eta-squared, epsilon-squared, omega-squared, Cohen *d*, Hedge correction, Glass delta), analysis of variance, crosstabs tabulations (Table 2), receiver operating characteristic curves, regression analysis, univariate general linear model (UGLM), poisson regression, and binary logistic regression. Exact sig. 2-sided are reported.

**Table 2:** Standardized total, direct, and indirect effects through path analysis with serial mediation in AMOS.

	Total effects				Direct effect					Indirect effects						
Hypothesis	*β*	SE	*T*	*P*	*β*	SE	*T*	*P*	Hypothesis	*β*	SE	*T*	*P*	Percentile bootstrap 95% CI		Result
														L	U	
RANIgE → BMI	0.426	0.149	2.85	**.022**	0.163	0.172	0.94	.306	RANIgE → (RE + AHI) → BMI	0.263	0.165	1.59	.155	−0.041	0.503	
RANIgE → RE	0.459	0.121	3.79	**.002**	0.459	0.121	3.79	**.002**								
RE → BMI	0.296	0.255	1.160	.326	0.296	0.255	1.16	.326								
RANIgE → AHI	0.323	0.161	2.00	**.082**	0.323	0.161	2.00	**.082**								
AHI → BMI	0.395	0.290	1.36	.295	0.395	0.290	1.68	.295								
AT or AE → BMI	−0.286	0.117	−2.44	**.021**	−0.056	0.168	−0.33	.605	AT or ED → (RE + AHI) → BMI	−0.230	0.149	−1.54	.212	−0.413	0.077	
AT or AE → RE	−0.378	0.123	−3.073	**.006**	−0.378	0.123	−3.07	**.006**								
AT or AE → AHI	−0.300	0.124	−2.41	**.025**	−0.300	0.124	−2.41	**.025**								

*β* = estimate, bootstrap 5000, AE = allergen eviction, AHI = apnea-hypopnea index, AMOS = analysis of a moment structure, AT = asthma treatment, BMI = body mass index, CI = confidence intervals, L = lower, RANIgE = respiratory and non-IgE mediated allergies, RE = respiratory effort, SE = standard error, U = upper.

We used the initial statistical analysis (correlations, exploratory factor analysis, principal factor analysis, regression analysis) to evaluate the principal variables examined: ATED, RANIgE, AHI, RE, BMI, and obesity/overweight. The supplementary variables (RDI, oxygen desaturation index, SF, SFVO) were thus, not applied in all subsequent statistical studies.

We tested the clinical relevance of the statistical significance and effect sizes^[27]^: (a) ATs and allergies interpreted the PG parameters more accurately and (b) lower RE and absence of allergies corresponded to lower BMI. This provides an internal validation of the present study.

Path analysis (analysis of a moment structure 28) was used to assess the model (Figure S1, Supplemental Digital Content, https://links.lww.com/MD/P657). For bootstrapping, no missing values were permitted, to be able to calculate 2-tailed *P* values in the indirect effects, so only 32 N were included. The data were screened and examined for assumptions of path analysis. The variables in path analysis are: ATED/RANIgE/RE/AHI/BMI.

Based on the rule of thumb (5–10 N/parameter), the estimated sample size was 50 N.

Included: 78 N to avoid omitting eligible children (bias in patient selection).

We excluded 1 child (noncompliant), 2 siblings (the second from 2 brother pairs to respect the rule of independence), and one 2-year-old girl (severe obstructive OSA,^[18]^ initial follow-up in a tertiary center).

74 N remained in the study.

Follow-up: 40 N, >2 months; 3 N, >6 months; 19 N, 1 to 2 years; 18 N, until August 2023 (4–5.5 years).

Through e-mail/telephone interviews, we found that children who ceased follow-up belonged to one of the following categories:

1. ameliorated
2. moved abroad.
3. Parents avoided treating children’s allergies.

PG sensitivity/specificity has been explored as a screening test in populations positive for the disease.^[28]^ We did not perform PG/PSG in children without signs of SDB, as this would not have any clinical relevance for patients.^[28]^

Based on the requirement of a minimal sample size for sensitivity/specificity for positive disease,^[28]^ we set a 95% confidence interval, 5% error margin, and 0.8 power analysis.

For 50% prevalence, the minimum sample size for sensitivity/specificity was 20 N.^[28]^ At the end of the study, we verified that the estimated prevalence of positive disease corresponded to that initially calculated.^[28]^

## 3. Results

The children were positive for SDB-asthma/allergy association as they suffered from: (a) recurrent/chronic clinical signs of SDB, including snoring (98.4%); (b) asthma signs (90.4%) with an atopic profile (eczema, recurrent rhinitis/conjunctivitis, asthma) (94.3%); and (c) 71% confirmed allergies (RA/food allergies) (partly reported in Supplementary Text, Supplemental Digital Content, http://links.lww.com/MD/P846).

### 3.1. Statistical Package for the Social Sciences evaluated

**Figure 1.**
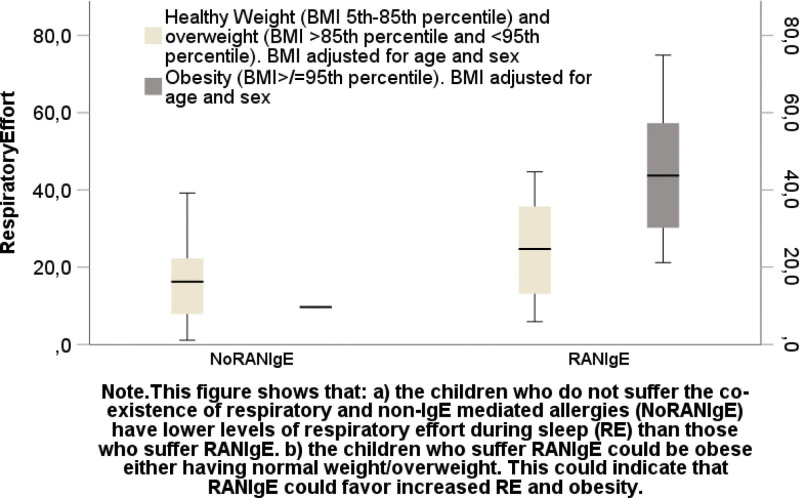
Clustered boxplot of the RE by RANIgE by obesity group. RANIgE = respiratory and non-IgE mediated allergies, RE = respiratory effort.

Percentages
  – Girls: 33.8%, boys: 66.2%.
  – Age: (*M*: 6.28 years, standard deviation = 3.18).
  – The children were addressed by parents (74.3%), ENT (8.1%), GP (14.9%), and dentists (1.4%).
  – BMI: HW (54.1%), growth stagnation (8.1%), overweight (16.2%), and obesity (21.6%).
  – RA: 57.1%, MA: 52.2%, RANIgE: 34%, IgEFA and NIgEFA: 8.1%.
Mean/median for skewed variables (BMI) of AHI, RE, BMI visualized in clustered bar means, multiple line means, boxplots to explore interactions and confounders, by group based on:
  – ATED (Figures S2A–C and S3A and B, Supplemental Digital Content, https://links.lww.com/MD/P657).
  – RANIgE (Figure S3C–E, Supplemental Digital Content, https://links.lww.com/MD/P657).
  - HWoverw/ vs obesity (Figure S4A, Supplemental Digital Content, https://links.lww.com/MD/P657 and Fig. 1).
  - obesity/overw vs HW (Figure S4B–G, Supplemental Digital Content, https://links.lww.com/MD/P657).
Correlations. AHI/RE correlated to BMI more than in between them:
– BMI/AHI, *r*(73) = 0.619, *P* < .001.
– BMI/RE, *r*(50) = 0.457, *P* < .001.
– AHI/RE, *r*(49) = 0.248, *P* = .085.
*t*-test. PG
– under ATED:
  – lower:
    – AHI(*M*), *t*(70) = −3.079, *P* = .004 effect size (*d* = 0.662).
    – RE, *t*(48) = 2.728, *P* = .009, effect size (*d* = 0.788).
– no RANIgE:
  – lower:
    – RE(*M*), *t*(32) = 2.896, *P* = .002, effect size (*d* = 1.237).

The effect of ATED/RANIgE on AHI/RE was superior to that of AT/ED/RA/NIgE alone.

**Figure 2.**
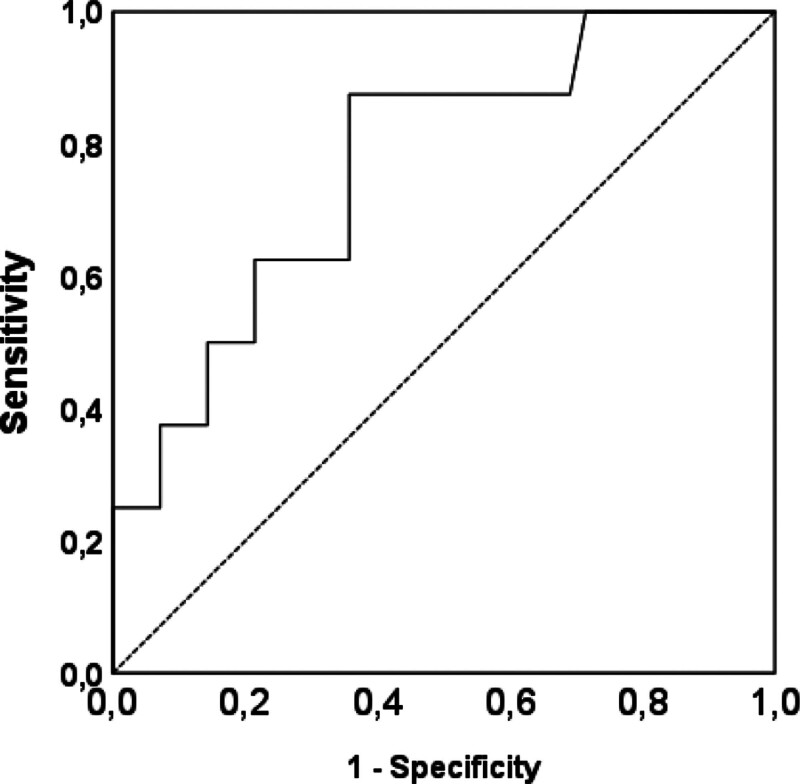
ROC curve (BMI adjusted for age and sex) of the RE to predict obesity (BMI ≥ 95th percentile) and overweight (BMI > 85th percentile and <95th percentile) versus normal weight (BMI 5th–85th percentile). BMI = body mass index, RE = respiratory effort, ROC = receiver operating characteristic.

Analysis of variance (Table S1, Supplemental Digital Content, https://links.lww.com/MD/P658)
  – ATED on AHI (eta: 0.317, eta-squared: 0.107) (*P* = .002) (*F* 7.793).
  – RANIgE for RE (eta: 0.523, eta-squared: 0.274) (*P* = .002) (*F* 11.694).
  – RANIgE effect on RE > 28% (eta: 0527, Somer *D*: 0.569).
Cross tabs (Table 1)
  – obesity/overweight; RANIgE, *Χ*^2^(1, N = 50) = 7.219, *P* = .012 (Somer *D* 0.450).
Number needed to treat (Table 1). ATED for:
  – AHI < 6.8 n/h: 4.9.
  – RE < 22%: 3.
  – avoiding obesity/overw: 3.8.
  Number needed to harm (Table 1)
– RANIGE for:
  – RE > 20%: 2.2.
  – obesity/overw: 3.1.
– RE ≥ 20% for:
  – obesity/overw: 3.7.
Receiver operating characteristic curves (Figures S5–S9, Supplemental Digital Content, https://links.lww.com/MD/P657) predicted:
– obesity/overw vs HW based upon:
  - RE (AUC = 0.769, *P* = .017) (Fig. 2).
  - AHI (AUC = 0.768, *P* = .004) (Figure S5, Supplemental Digital Content, https://links.lww.com/MD/P657).
  - RANIgE (AUC = 0.725, *P* = .029) (Figure S6, Supplemental Digital content, https://links.lww.com/MD/P657, Table S2, Supplemental Digital Content, https://links.lww.com/MD/P658).
– based upon RANIgE: To predict:
  - BMI (AUC = 0.755, *P* = .003) (Figure S7, Supplemental Digital Content, https://links.lww.com/MD/P657).
  - RE (AUC = 0.788, *P* = .007) (Figure S8, Supplemental Digital Content, https://links.lww.com/MD/P657).

**Table 1:** Statistical comparisons (cross tabs chi-square/Pearson chi-square) between groups (RANIgE, AHI > 6.8, AT, RE>20, RE>22, obesity/overweight), effect sizes, risk estimate, number needed to treat (NNT) and number needed to harm (NNH) are reported.

	χ^2^ (*df*, N), *P*	CC, *P*	Eta	Somer *D*, dependent (Dep), *P*	Kendall tau-c, *P*	Kendall tau-b, *P*	Phi, *P*	Cramer V, *P*	Risk	MH OR (*P*)	NNT	NNH
RANIgE×AHI>6.8	4.76 (1,49), **.04**	0.298	0.312	0.340	0.252	0.312	0.312	0.312	4.35	4.356 (***.035***)		*2.2*
AT×AHI>6,8	4,261 (1,72), **.067**	0.236	0.243	−0.288	−0.190	−0.243	−0.243	−0.243	−0.212	0.212 (***.054***)	4.9	
AT or AE×AHI>6,8	4,58 (1,72), **.042**	0.349	−0.372	−0.247	−0.204	−0.252	−0.252	0.252	0.241	0.241 (***.042***)	4.9	
RANIgE×RE>20	6.63 (1,33), **.014**	**0.409** ***P* = .014**	**0.449**	Symmetric: **0.448** RANIgE Dep: **0.433** RE>20 Dep: **0.464** *P* = **.014**	**0.430** *P* =**.014**	**0.449** *P* = **.014**	**0.449** *P* = **.014**	**0.449** *P* = **.014**	7.5	7.5 (***.014***)		*2.2*
RANIgE×RE>22	4.53 (1,33), **.066**	**0.348** ***Exact P* = .066** **Approx. *P* = .027**	**0.371**	Symmetric: **0.371** RANIgE Dep: **0.361** RE22 Dep: **0.381** ***P* = .066** Approx. *P* = **.027**	**0.353** Exact *P* = **.066** Approx. *P* = **.027**	**0.371** Exact *P* = **.066** Approx. *P* = **.027**	**0.371** Exact *P* = **.066** Approx. *P* = **.033**	**0.371** Exact *P* = **.066** Approx. *P* = **.033**	5.0	5.0 (***.039***)		*2.6*
RE>20×obesity/overweight	6.603 (1,50), **.017**	**0.342** ***P* = .017**	**0.363**	Symmetric: **0.347** RE20 Dep: **0.494** Obesity/overweight Dep: **0.267** *P* = **.017**	**0.266** *P* = **.017**	**0.363** *P* = **.017**	**0.363** *P* = **.017**	**0.363** *P* = **.017**	11.375	11.375 (***.029***)		3.7
Obesity/overweight×RE>20	6.603 (1,50), **.017**	0.342	0.363	0.267	0.266	0.363	0.363	0.363	11.375	11.375 (***.029***)		*2*
RANIgE×obesity/overweight	7.219 (1,50), **.012**	0.355	0.380	0.450	0.288	0.380	0.380	0.380	7	7.0 (***.013***)		3.1
Obesity/overweight×RANIgE	7.219 (1,50), **.012**	0.355	0.380	0.321	0.288	0.380	0.380	0.380	7	7.0 (***.013***)		2.2

Effect sizes were reported by evaluating the contingency coefficient (CC)/Eta/Somer D/Kendall tau-c/Kendall tau-b/Phi/Cramer V. To evaluate the risk of disease, we reported the risk estimate (risk)/Mantel–Haenszel odds ratio estimate (MH OR). The number needed to treat (NNT) to avoid the outcome, and the number exposed to harm (NNH) to achieve the outcome were also evaluated.

Italicized values indicate statistical significance.

Bold values denote effect sizes that are at least medium or close to medium in magnitude. For Cohen’s D, effect sizes were interpreted as small (.2), medium (.5), and large (.8), with values <.2 considered negligible.

For Eta effect sizes were interpreted as small (.01), medium (.06), and large (.14). Values showing medium or larger effects, or consistently approaching medium effects across reported parameters, are highlighted in bold.

AE = allergen eviction, AHI = apnea-hypopnea index, AT = asthma treatment, RANIgE = respiratory and non-IgE mediated allergies, RE = respiratory effort, RE>20=RE>20%, RE>22= RE>22%, obesity/overweight=obesity and overweight vs healthy weight, SE = standard error.

*P* = exact *P*/approximate (Approx.) *P* is reported when exact *P* > .5.

ATED did not have an accurately predict obesity (Figure S9, Supplemental Digital Content, https://links.lww.com/MD/P657).

- A multiple UGLM was evaluated (Tables S3–S18 and S26–S32, Supplemental Digital Content, https://links.lww.com/MD/P658).
- Profile marginal plot: the RE remained lower under ATED when considering RANIgE (Figure S10, Supplemental Digital Content, https://links.lww.com/MD/P657).
- A UGLM was used to investigate whether a combined variable, ATED.RANIgE, predicted RE. The overall model: *F*(3, 32) = 12.442, *P* < .001, *R*^2^ = 0.563, and adjusted *R*^2^ = 0.518. ATED.RANIgE significantly independently predicted RE, *F*(3, 32) = 12.442, *P* < .001 (Tables S16 and S17, Supplemental Digital Content, https://links.lww.com/MD/P658).

**Figure 3.**
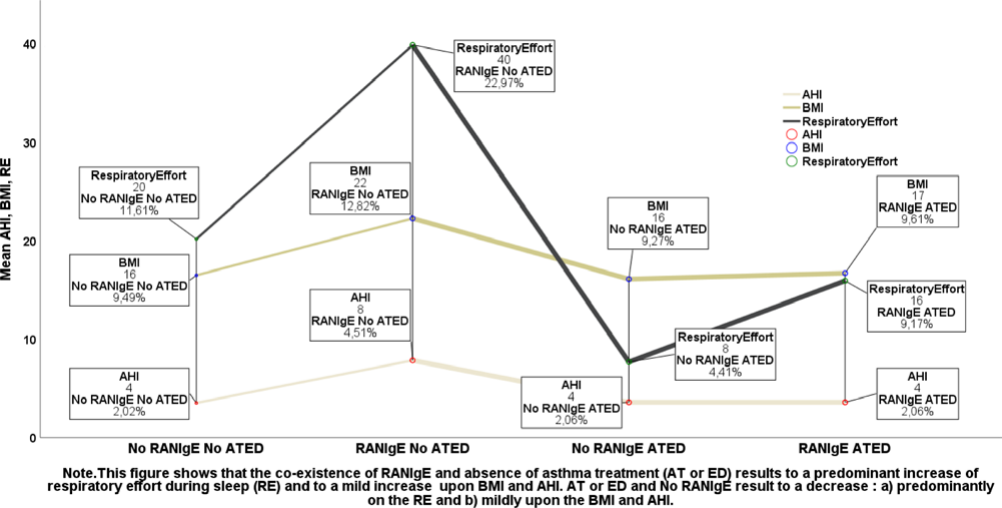
Mean values of AHI, BMI, and RE in children under AT or AE, either with no AT or AE, when RANIgE variable has been considered. The representation of both AT or AE and RANIgE is effectuated through a combined (dummy) variable (ATED.RANIgE) representing both the treatment (AT or ED) and the coexistence of RANIgE. AE = allergen eviction, AHI = apnea-hypopnea index, AT = asthma treatment, BMI = body mass index, ED = eviction diet, RANIgE = respiratory and non-IgE mediated allergies, RE = respiratory effort.

Post hoc comparisons (least significant difference): RE highest for the RANIgENoATED group and lowest for the NoRANIgE.ATED group (Fig. 3, Table S18, Supplemental Digital Content, https://links.lww.com/MD/P658, and Figures S11 and S12, Supplemental Digital Content, https://links.lww.com/MD/P657).

**Figure 4.**
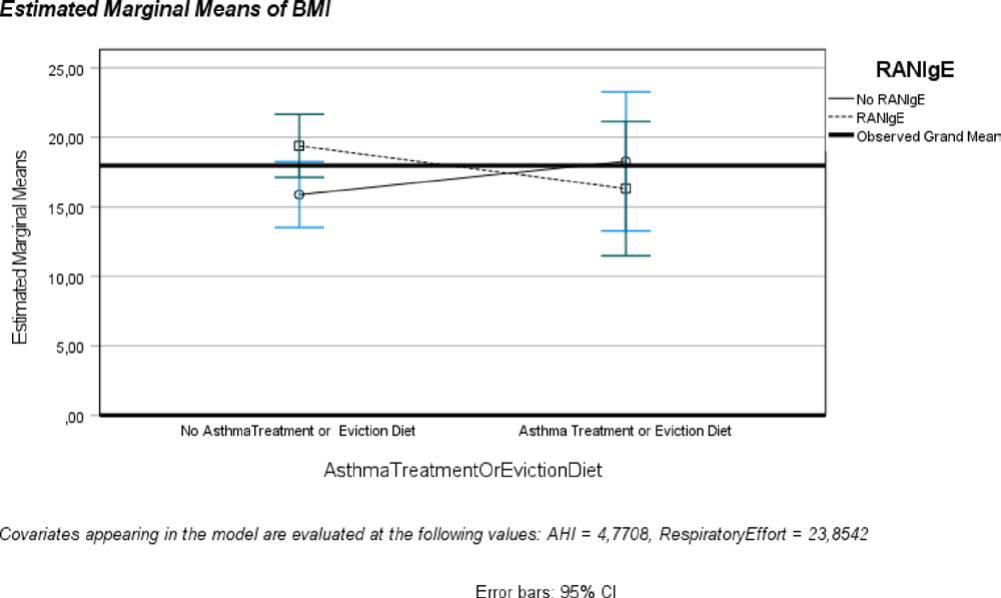
Profile plot of BMI according to both AT or ED and RANIgE. AT = asthma treatment, BMI = body mass index, ED = eviction diet, RANIgE = respiratory and non-IgE mediated allergies.

**Figure 5.**
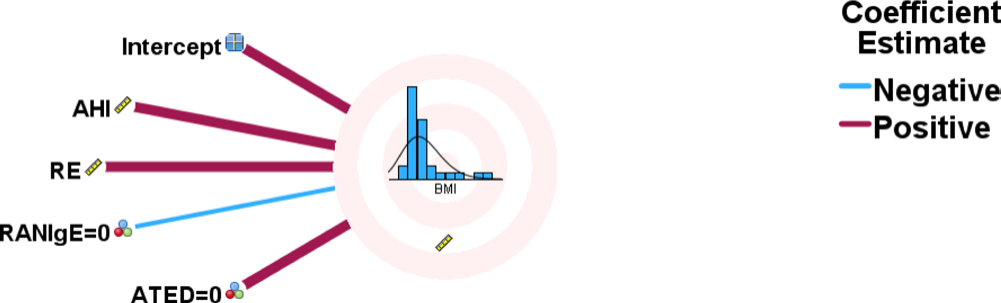
A generalized linear mixed model (GLMM) identified positive effects of AHI/RE/No ATED and a negative effect of the absence of RANIgE on BMI. AHI = apnea-hypopnea index, ATED = asthma treatment or eviction diet, BMI = body mass index, RANIgE = respiratory and non-IgE mediated allergies, RE = respiratory effort.

- Poisson regression: ATED and RE predicted BMI.
  – *Χ*^2^(5.982): *P* = .050 (Table S19, Supplemental Digital Content, https://links.lww.com/MD/P658). Deviance/df: 1.082, log Likelihood: −30.063, Bayesian Information Criterion: 67.321, Akaike’s Information Criterion (AIC): 69.555, and CAIC: 70.321 (Table S20, Supplemental Digital Content, https://links.lww.com/MD/P658).
  – RE significantly predicted BMI (*B* = 0.009, standard error = 0.0035, *P* = .014), but ATED did not (*B* = -0.074, standard error = 0.2025, *P* = .715) (Table S21, Supplemental Digital Content, https://links.lww.com/MD/P658).
- Profile plots evaluated:
  – RE by RANIgE by HWoverw/ vs obesity (Figure S13, Supplemental Digital Content, https://links.lww.com/MD/P657).
  – AHI by ATED by HWoverw/ vs obesity (Figure S14, Supplemental Digital Content, https://links.lww.com/MD/P657) and by obesity/overw vs HW (Figure S15, Supplemental Digital Content, https://links.lww.com/MD/P657).
  – The lines intersect, indicating interactions between AHI/RE, ATED/RANIgE, and obesity groups.
- Binary regression GLM indicated that, all else being equal, subjects having lower RE had fewer odds of having the outcome “obesity” than subjects having increased RE (OR = 4.120; 95% confidence intervals: −0.104 to −0.002; *P* = .042) (Tables S22–S24, Supplemental Digital Content, https://links.lww.com/MD/P658). Bayesian Information Criterion: 47.703, AIC: 41.967, CAIC: 50.703 (Table S25, Supplemental Digital Content, https://links.lww.com/MD/P658).
- UGLM investigated whether ATED and RANIgE predicted BMI while controlling for AHI/RE as covariates (Tables S26–S30, Supplemental Digital Content, https://links.lww.com/MD/P658): *F*(6, 23) = 6.336, *P* = .001, *R*^2^ = 0.691, adjusted *R*^2^ = 0.582. ATED × RANIgE × AHI (Table S31, Supplemental Digital Content, https://links.lww.com/MD/P658) significantly interacted with [AT or ED = 0] × [RANIgE = 1] × AHI (*P* = .001) (Table S32, Supplemental Digital Content, https://links.lww.com/MD/P658). The profile plot lines intersect predicting BMI with AHI/RE as covariates (Fig. 4), indicating interactions between RANIgE/ATED/BMI and contributing to the path analysis.
- A generalized linear mixed model identified positive effects of AHI/RE/No ATED and a negative effect of the absence of RANIgE on BMI (Fig. 5, Figure S16A and B, Supplemental Digital Content, https://links.lww.com/MD/P657, and Tables S33–S44, Supplemental Digital Content, https://links.lww.com/MD/P658).

### 3.2. Path analysis with serial mediation

Assessment of normality: no strong violation of normality. Multivariate kurtosis: 6.946; critical ration: 2.348 (Table S45, Supplemental Digital Content, https://links.lww.com/MD/P658). Multicollinearity was not observed (Tables S46 and 47, Supplemental Digital Content, https://links.lww.com/MD/P658). The model is recursive and overidentified. The residual plots suggest that the assumptions of linearity and homoscedasticity were tenable. Two multivariate outliers were detected which remained in the analysis.

Chi-square: 0.317, df: 1, *P* = .574, comparative fit index: 1.0, root-mean-square error of approximation: 0.000, incremental fit index: 1.017, normed fit index: 0.992, PCLOSE: 0.588 (90% confidence intervals: 0.000 to 0.391), AIC: 38.317.

### 3.3. Bollen–Stine bootstrap

Assessing the null hypothesis that the model is correct, our model fit the data very well, *P* = .718.

### 3.4. Parameters’ estimation

**Figure 6.**
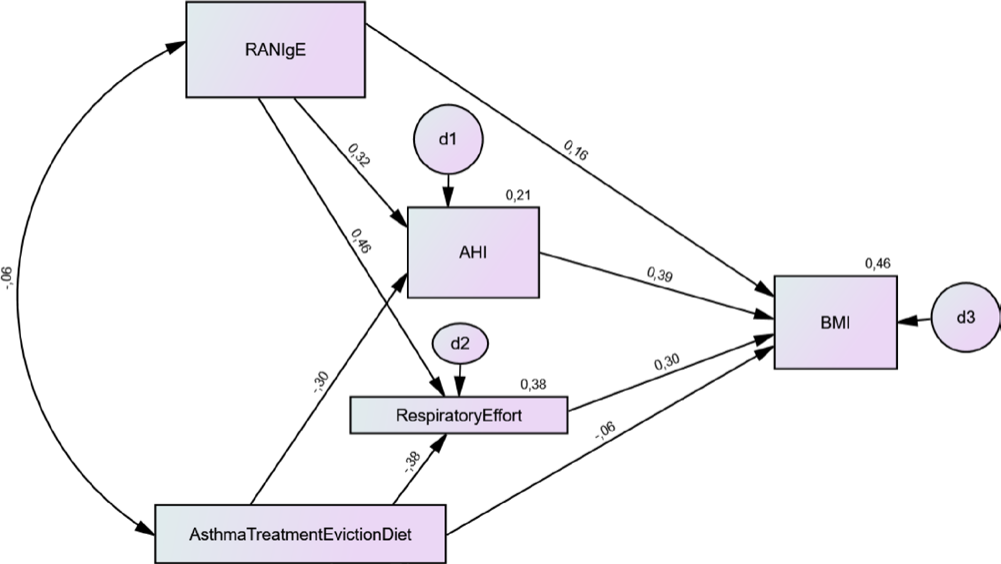
Standardized estimates of the direct effects of the path analysis.

Path coefficients for direct effects represent the regression coefficients in multiple regressions (unstandardized/standardized). The regression weights (Table S48, Supplemental Digital Content, https://links.lww.com/MD/P658), squared multiple correlations (Table S49, Supplemental Digital Content, https://links.lww.com/MD/P658), parameter estimates for standardized (Table 2, Fig. 6) and unstandardized (Table S50, Supplemental Digital Content, https://links.lww.com/MD/P6580 and Figure S17, Supplemental Digital Content, https://links.lww.com/MD/P657), and user-defined estimates for the indirect effects (Table S51, Supplemental Digital Content, https://links.lww.com/MD/P658) are reported.

RANIgE total and direct effect on RE (*P* = .002),

– RANIgE total effect on BMI (*P* = .022).
– RANIgE direct/indirect effects on BMI: nonsignificant.

The overall effect of RANIgE on BMI was mediated by its significant effect on RE.

ATED total/direct effect on:

– RE (*P* = .006)
– AHI (*P* = .025).

ATED total effect on BMI (*P* = .021).

ATED direct/indirect effect on BMI are not significant.

Similar to the effect of RANIgE on BMI, the total effect of ATED on BMI is mediated by the significant effect of ATED on BMI.

### 3.5. Moderators’ and mediation effect

ATED/RANIgE is a moderator that influences AHI/RE which in turn affects BMI. Mediated moderation occurs as the effect of exposure to RANIgE is greater in high-risk subjects (not under ATED). The interaction of RANIgE/ATED affects the mediating variable (AHI/RE), which influences BMI.^[29]^

## 4. Discussion

Allergic children experienced a significant SF despite a low AHI since the onset of RA, which correlated with an increase in RE/BMI^[20]^ and a decrease in REM^[10,11]^ (cases 5 and 6, Supplementary Text, Supplemental Digital Content, http://links.lww.com/MD/P846).^[10,11]^

PG/PSG under ATED was inconclusive. RE/AHI decreased under ATED, which treats inflammation, and increased without ATED. Parents resisted mentioning ATED, which encompasses intermittent treatments, oral corticosteroids, and ongoing ED without a specific medical diagnosis, indicating reluctance to explore and diagnose allergies or asthma. This identifies the burden on these children, which deserves our attention, and has not been accurately identified until now.

Orthodontic complications are common in OSA.^[30]^ Therefore, we advised our patients to consult an orthodontist.^[31]^ Several children had already wore braces (Repository Text, Supplemental Digital Content, http://links.lww.com/MD/P846). However, the OSA symptoms persisted. Others (usually <6-year-old) had oral disorders that may accompany NIgE allergies. Children were 6-year-old (*M*: 6.28 years, standard deviation = 3.18), and even young as 3-year-old. Orthodontic interventions are not routine practice for children. Studies on facial characteristics and orthodontic treatments in pediatric OSA are usually performed in older children (aged 8 years).^[30,32]^ The increased RE evaluated persistent ENT inflammation and induced obstruction, which could distort the development of children’s dental, facial, and oropharyngeal structures, and growth in the early stages of facial structure expansion through a constant fight of facial muscles to liberate the upper airways from diffuse inflammation.

We propose that a decrease in RE during sleep could contribute to healthier development of dental and oropharyngeal structures, thus avoiding facial abnormalities and orthodontic complications in elderly children with OSA.

We present 2 cases of OSA-obstructive origin, a genetic syndrome with skeletal/facial abnormalities, and another with a history of prematurity to distinguish OSA-obstructive origin from SDB, potentially evolving into associated OSA-asthma.^[18,19]^ Distinguishing facial abnormalities since birth (genetic syndromes/prematurity) from facial particularities evolving later would help to differentiate OSA-obstructive origin and non-obstructive through PG parameters.

We investigated RE as a physiological mechanism impacting facial growth in the absence of a clear obstruction in children with OSA and comorbidities such as asthma and obesity, which were not explained through facial characteristics. Allergic obese children gain weight while exhibiting significantly increased RE, despite being on continuous positive airway pressure.^[20,22]^ Increased RE during sleep may be a physiological mechanism contributing to obesity.

PGs/PSGs were effective during periods free off febrile illnesses or asthma attacks. Increased RE indicates unresolved inflammation, favoring asthma, corticosteroid use, and obstruction, thus increasing metabolic burden.

Nearly half of the children suffer from recurrent nonfebrile illnesses (otitis, bronchitis, rhinitis) and BMI disorders accompanied by SDB, contributing to constant stress and promoting cortisol hypersecretion, and obesity.^[22,33,34]^

The children slept for an adequate duration of time. TST was normal (mean TST = 8 hours 37 minutes).

Adult sleep deprivation typically results in extreme daytime sleepiness. However, the children were excited and hyperactive during the daytime and had difficulty falling asleep. Clinical signs were consistent with related stress and increased RE, impairing children from falling asleep easily, remaining asleep, and physically and mentally having a restorative sleep.

Most of the children experienced MA. Mites seek humid environments, such as beds. Nocturnal transpiration in children with allergies favors a vicious SDB circle.

A child suffering NIgEFA (case 10, Supplementary Text, Supplemental Digital Content, http://links.lww.com/MD/P846) developed a delayed positive skin prick tests to mites, which subsided after specific mites’ eviction and ED (Figure S18A–D, Supplemental Digital Content, https://links.lww.com/MD/P657). NIgE allergies seem to favor RA onset, and treatment of Th1 inflammation (NIgEFA) seems to prevent the onset of Th2 inflammation (RA).

Children with both RA/NIgE allergies (Th1 and Th2 inflammation) presented with the greatest increase in RE during sleep and suffered the most from obesity. The metabolic burden of allergies owing to increased RE reflects the stress mechanisms that burst when RA is combined with NIgE allergies.

RANIgE favor OSA-asthma via ENT inflammation.^[21]^

The effect of RANIgE on BMI surpassed the negative effects of ATED, thus prompting RANIgE treatment.

The cumulative effect of ED/AT on RE/AHI/BMI explains the role of gastroesophageal reflux disease in asthma and its amelioration following ED. An increase in RE is an effective method for

1. identifying the preschool children suffering from OSA-asthma-associated, and
2. measuring the efficacy of an ED/AT.

We identified only 6/74 (8.1%) IgEFA cases, supporting the delayed mechanisms involved in the association between OSA and asthma.

Milk, wheat, and soy were the main NIgEs. Dairy, wheat, and soy additives such as fast-acting carbohydrates are the principal ingredients of industrialized foods and children’s favorites. They represented the major core of adolescents’ alimentation as they did not try to follow an ED.

We explored whether RE was only a characteristic (and thus a trait) of obese children (which it is), as well as the causality of obesity. If RE only characterized obese children, then we could only perform weight interventions to decrease the BMI.

If RE is the cause of obesity, it could alert us to identify high-risk children and the factors provoking an increase in RE, establish easily applicable health policies, and avoid obesity and its deleterious consequences.

Regression analysis was used to identify the important parameters that explain obesity. Nevertheless, the significant interaction between RANIgE × ATED × AHI prevented us from identifying valuable parameters due to their interactions (Tables S26–S32, Supplemental Digital Content, https://links.lww.com/MD/P658). The generalized linear mixed model and path analyses elucidated the causal relationships between the variables. The Bollen–Stine bootstrap confirms that our model fits the data very well. A graphical model (parameter estimates) showed that ATED/RANIgE levels inversely affected the BMI by moderating AHI/RE. Therefore, subjects at high risk for the RANIgE effect were not under ATED.

Moderate-to-large effect sizes were verified in various ways (Somer *D*/eta/eta-squared, and Cohen *d*/Hedge correction/contingency coefficient/Phi/Kendall tau-b). Therefore, we did not need a large sample size to reach significant conclusions.^[27]^

The results could be probabilistic given the substantial magnitude of the effects and the adequate and representative sample size of the pediatric population afflicted with coexisting SDB/allergies/asthma, commonly referred to as OSA-asthma associated.

The acquired knowledge regarding ATEDs/allergies assisted us in providing effective advice to the patients. Case 1 (Supplementary Text, Supplemental Digital Content, http://links.lww.com/MD/P846) became obese after the T&A despite adequate weight intervention and corticosteroid cessation. His BMI decreased only after the initiation of sublingual immunotherapy.

Increased RE during sleep inherently correlates with SDB/OSA related to allergies, especially RANIgE, and is the origin of SF in OSA asthma, even if AHI remains low. It decreases (similar to AHI) under ATED, and if untreated, contributes to an increase in AHI and OSA persistence. OSA comorbidities underlie a physiological mechanism that evolves in a deleterious manner, in parallel and independently of AHI. If AHI was the only mechanism governing OSA, OSA would be cured once AHI decreased, and no comorbidities would evolve. RE is the underlying mechanism favoring OSA comorbidities, which persist if the diagnosis and treatment are only focused on AHI.

Therefore, SF can alert physicians, even those with low AHI. An increase in SF/RE can be the root cause of REM sleep disturbance, hyperactivity, behavioral/orthodontic problems, and obesity in children with allergies through a burst of stress-related mechanisms. The increase in RE could detect high-risk children prone to developing an OSA-asthma association early, thereby avoiding unnecessary operations and preventing the loss of time to apply appropriate personalized treatment. Pediatric-OSA-asthma can be renamed pediatric-OSA-allergy. Therefore, attention should be paid to early diagnosis and treatment of allergies. An easy guideline for GPs/ENT/pediatricians could be to test for NIgEFA (milk/wheat/soy) and perennial RA (mites/Alternaria alternata) in preschool children with non-obstructive SDB.

The early diagnosis and treatment of allergies should be prioritized to prevent sleep disorders and their deleterious consequences. Any alimentation that disturbs children’s sleep and favors stress pathways should be avoided. Therefore, obese children should not wait for allergies to be detected. Public health policies should focus on alerting physicians to the early detection of allergies in order to prevent asthma, OSA, and obesity. AT/ED/allergies significantly affected SDB/OSA/BMI, providing valuable insights for personalized and comprehensive treatment plans for affected children, and potentially mitigating pediatric asthma and obesity.

## 5. Conclusion

SF (through AHI/RE increase) mediates the pathway to OSA, asthma, and obesity, whereas allergies/ATEDs moderate (increase/decrease) the effects of SF on asthma, OSA, and obesity.

## Acknowledgments

The authors specially acknowledge all his patients and their parents. The authors thank Dr François Lavaud, Service de Pneumo-Allergologie, CHU Reims, France; Dr Claude Ponvert, Faculté́ de Médecine Paris-Descartes, Service de Pneumo-Allergologie Pédiatrique, Hôpital Necker-Enfants Malades, Paris, France; National Board of French Allergists, Conseil National Professionnel d’Allergologie (CNPA); French National Allergy Association, Société Française d’Allergologie (SFA); Syndicat Français des Allergologues (SYFAL); Direction Recherche Ramsay Santé; Vincent Lechat, Sleep Technician, Paris, France; Stefan Ljubisavljevic, Sleep Technician, Paris, France; Thierry Gogneau, Paris, Hauts-De-France, Normandie, Champagne, Ardennes, France; Pierre Falzone, Sleep technician, Liège, Belgium; Dr Larisa Anghel, University of Medicine and Pharmacy in Iasi.

## Author contributions

**Conceptualization:** Kalomoira Kefala.

**Data curation:** Kalomoira Kefala.

**Formal analysis:** Kalomoira Kefala.

**Investigation:** Kalomoira Kefala.

**Methodology:** Kalomoira Kefala.

**Project administration:** Kalomoira Kefala.

**Resources:** Kalomoira Kefala.

**Software:** Kalomoira Kefala.

**Supervision:** Kalomoira Kefala.

**Validation:** Kalomoira Kefala.

**Visualization:** Kalomoira Kefala.

**Writing – original draft:** Kalomoira Kefala.

**Writing – review & editing:** Kalomoira Kefala, Philippe Guerin.

## Supplementary Material






