# Supplementary material for: Allergies, asthma treatment, and eviction diet have a significant impact on the respiratory effort during sleep and the apnea-hypopnea index in children with obstructive sleep apnea-obesity/asthma association: A STROBE-compliant study
Source: Medicine (Baltimore). 2026 Feb 13;105(7):e41730. doi: 10.1097/MD.0000000000041730 (PMC12908835; doi:10.1097/MD.0000000000041730)
Supplement: Supplementary file 1 [file medi-105-e41730-s001.docx]

**Repository Text**

**Case reports:**

In this section, we present several cases illustrating the burden of sleep disorders related to allergies, the interactions between asthma, SDB, hyperactivity, concentration and behavioural problems, stagnation of growth or overweight, and difficulties related to diagnosis, treatment, and follow-up of patients(11-16, 18, 20).

• Case 1. A 9.5-year-old obese boy presented with RANIgE (wheat) and severe sleep disorders. He underwent adenotonsillectomy and stopped snoring. However, he continued to gain weight despite follow-up with a nutritionist. He decided to discontinue asthma drugs (inhaled corticosteroids) because he thought that these drugs were at the origin of his obesity. Nevertheless, the patient’s BMI continued to increase. The patient finally decided to start sublingual immunotherapy at 12 years of age. Four months after the onset of immunotherapy, he started losing weight, although he did not modify his dietary habits.

• Case 2. An 11-year-old boy presented with progressive recurrent rhinitis, otitis, asthma exacerbations, constipation, abdominal pain, severe sleep disorders, and obesity. He had RANIgE (wheat) and was exposed to passive smoking from both parents. The patient had no family history of allergies. He followed a short eviction diet and stopped experiencing digestive signs. He was set on Continuous Positive Airway Pressure (CPAP), and he had an improvement; however, he could not get rid of the CPAP. After the onset of sublingual immunotherapy, he managed to remove CPAP and stabilize his weight (11, 20, 21).

• Case 3. A 3.5-year-old boy presented with progressive recurrent gastroesophageal reflux, bronchiolitis, rhinitis, otitis, bronchitis, eczema, asthma, severe sleep disorders, diurnal signs of repercussion, severe OSA, and severe obesity. The patient had RANIgE (wheat). He found it difficult to follow the eviction diet. Sublingual immunotherapy was not initiated because the patient was too young. He underwent an ENT operation and managed to get rid of the CPAP. However, his BMI continued to increase from 31 to 44 at 7 years of age, although he consulted an obesity center (20, 21).

• Case 4. A 16-year-old obese boy (BMI: 84 kg/m2) was examined for exercise-induced asthma and sleep disorders. Spirometry was normal (FEV1/FVC, 86.2%; FEV1:100%; FVC:121%). However, DEM25/75(L/s) was decreased (73%-67%/theoretical value). The patient had recurrent asthma exacerbations and required recurrent oral corticosteroids. Overnight PG revealed severe OSA (AHI: 32n/h). He was set on CPAP, but could not tolerate it. An allergy workup revealed non-IgE-mediated allergies to milk and wheat. He also suffered recurrent constipation and abdominal pain and was advised to follow a two-months eviction diet of milk and wheat with prescription of an amino-acid formula. He came back two years later, accompanied by his 2-year-old sister for allergy exploration. His BMI had decreased to 25 kg/m^2^. His mother confirmed that he followed the eviction diet, did not use any other oral corticosteroids, and could tolerate exercise provided he used a short-acting bronchodilator before exercise.

• Case 5. A 6-year-old boy had  severe sleep disorders, mild OSA, and secondary enuresis with diurnal repercussions and behavioural disturbances for several years, in parallel with chronic (severe persistent) rhinitis, eczema, and gastrointestinal signs since he was an infant. The digestive symptoms were ameliorated by empiric milk eviction. Follow-up by a psychologist was ineffective for the sleep disorders. An allergology work-up revealed mild sensitization to mites, but the parents doubted that this sensitization could be responsible for his sleep and behavioural problems. PSG revealed microarousals that were not explained by respiratory events. Treatment with mite eviction, nasal corticosteroids, antihistamines, and oral anti-leukotrienes had no effect on his sleep and behavioural problems, but specific sublingual mite immunotherapy induced rapid (3 months) resolution of these problems, with no recurrence in a three-year follow-up (12, 13).

• Case 6. A 9-year-old boy with uncontrolled allergic asthma and mite allergy was examined for severe persistent sleep disorders despite being set on asthma treatment with inhaled corticosteroids and oral anti-leukotrienes. PG performed under asthma treatment could not identify the origin of his sleep disorder. PSG, also performed during asthma treatment, revealed micro-arousals that were not explained by respiratory events. The parents did not accept allergen immunotherapy (AIT) because they doubted that allergies to mites could be responsible for the sleep disorders (13).

• Case 7. A 3.5-year-old girl was diagnosed with recurrent bronchitis, rhinitis, otitis, and a sleep disorder. The patient’s condition was significantly ameliorated with asthma treatment, but PG under asthma treatment revealed mild OSA. Allergology work-up revealed a non-IgE-mediated allergy to wheat and milk, and SPT was slightly positive for mites. She was prescribed a specific mite eviction, but could not follow the AE. Two years later, she returned with recurrent cough, abdominal pain, diarrhoea, eczema, and sleep disorders. She underwent asthma treatment again, but his asthma did not improve. Allergology work-up was diagnosed again (patch tests) as a non-IgE-mediated allergy to wheat and milk, although she did not consume milk since the age of 2 years, with negative SPT to common aeroallergens. She started serious eviction of wheat and milk with calcium supplementation, and her digestive symptoms, recurrent cough, eczema, and sleep disorders were significantly ameliorated.

• Case 8. A 7-year-old boy under milk eviction since infancy was explored for stagnation of growth with constant severe fatigue and irritability, impairing his daily ordinary activities (especially school presentations). His mother and brother had pollen and mite allergies. Skin tests were performed when the patient was diagnosed with non-IgE-mediated allergies to milk, wheat, and soymilk. A two-month wheat eviction induced amelioration of constipation. At the age of 5 years, he had OSA confirmed as PG. Tonsillectomy did not ameliorate diurnal symptoms. ENT revealed no obstacles in the upper airways, but PSG revealed severe OSA. CPAP ameliorated the child’s growth, but he still had severe difficulty falling asleep and getting up in the morning. Follow-up at a sleep center did not ameliorate his sleep problems. Skin prick tests performed at 7 years of age revealed mild sensitization to mites. The mother did not want to follow allergen eviction or other allergen-specific treatments.

• Case 9. A 7-year-old obese boy (BMI: 24.6 kg/m2) was examined after a GP request for chronic rhinitis for a two-year-period despite adequate use of nasal sprays. He had eczema since early infancy, chronic sinusitis, recurrent tracheitis, conjunctivitis, otitis, recurrent urticaria, persistent constipation, gastroesophageal reflux, hyperactivity, aggressiveness towards his siblings, dyspraxia, obesity, orthodontic problems, and speech problems. Although he was treated with speech therapy, he did not snore, slept with mouth open, and had a normal sleep (21h-7h). Allergy tests revealed a mite allergy (IgE d1:71.30 kU/L). An overnight PG revealed an AHI (3.4n/h), an abnormal Oxygen Desaturation Index (ODI: 7.2 n/h), and an increased RE (43.7%). Two months later, patch tests were positive for milk and wheat, and strongly positive for soymilk. A control PG performed at the same time, revealed AHI: 21.2 n/h, ODI: 14.8 n/h, RE: 28.3%. The parents preferred to continue the ongoing speech therapy, orthodontic, and ENT follow-up, and they would not start mite AIT.

• Case 10. A 5-year-old boy was initially diagnosed with asthma but no associated allergies. He had undergone adenotonsillectomy at the age of 2 years, but with no amelioration. There had no family history of allergies. Passive smoking was observed by the father but away from the child. He had a severe nocturnal cough, recurrent laryngitis, and asthma symptoms, followed by an asthma center. Initial allergy exploration was performed in this center, but the SPT was negative for common aeroallergens at ages 4 and 5 years. Airway resistance increased at the age of three years. At 5 years of age, pulmonary function test results were normal. No immediate-type allergies were detected. Although he was treated for severe asthma with a combination of long-acting beta2-agonists, high-dose inhaled corticosteroids, and anti-leukotrienes, he frequently used short-acting beta2-agonists and recurrently needed oral steroids and nebulized corticosteroids. Owing to mild OSA, he was set on CPAP, which induced complete remission of his nocturnal cough, and the child could progressively cease the use of asthma drugs.

He returned for exploration three years later for severe eczema, constant pruritus, digestive symptoms, and constant fatigue. Prick-tests performed with common aeroallergens and food allergens yielded negative immediate responses, but were positive at 24h for mites and milk (Supplementary Figure 17A-D). The SPT to milk continued to increase further (Supplementary Figure 17B), although the SPT to mites did not increase further (Supplementary Figure 17C). Patch tests were positive for milk, wheat (Supplementary Figure 17D), and soymilk. The child followed an eviction diet for milk, wheat, and soy milk and presented with clinical amelioration. Seven months later, the SPT reaction to milk or mites was no longer delayed.

**SPSS analysis**

**Percentages**

The questions that the parents left as non-answered were those usually considered non-bothersome for them [recorded as missing in **Online Repository**]. The parents reported supplementary clinical signs not initially included in the questionnaire; [the numbers reported as missing corresponded to those who did not record these supplementary signs].

Demographic characteristics:

1) The children were aged 2-16-year-old (*M*: 6.28 years, SD = 3.18). Girls (F): 25/74 (33.8%), boys (M): 49/74 (66.2%) (ratio M/F = 1.45).

2) Children were addressed by (missing: 1/74): a) the parents themselves: 55/73 (74.3%), b) the ENT: 6/73 (8.1%), c) the GP: 11/73 (14.9%), d) the dentist: 1/74 (1.4%).

3) BMI classification:

a) Normal weight (BMI percentile range in, 5%-85%): 40/74 (54.1%)

b) stagnation of growth (BMI percentile range < 5%): 6/74 (8.1%)

c) at risk of overweight (BMI percentile range 85-95%): 12/74 (16.2%)

d) Obese (BMI percentile range > 95%): 16/74 (21.6%).

4) Positive Family history of allergy/asthma32/44 (72.7%) (missing: 30/74, 40.5 %).

5) Domestic environmental exposure (when they were known):

a) tobacco exposure: 20/29 (69%),

b) humidity in the apartment/house 5/74 (6.8%)

c) pets living at home: 6/21 (28.6%).

6) Removal of tonsils/adenoids (T/A) with no improvement in 18/41 (valid 43.9%) [missing: 60/74 (81.1%)]. Some of them had removed the T/A before the age of 2 years.

7)  Clinical signs of SDB:

a) during falling asleep:

• Difficulties falling asleep: 11/13 (14.9%) (missing 61/74, 82.4%),

• 3/5(4.1%) felt they could not breathe while falling asleep [missing 69/74, 9(3.2%)].

b) during sleep:

• 60/61 (98.4%): snoring (missing 13/74, 17.6%).

• 26/27 (96.3%): slept with mouth open (missing 47/74, 63.5%).

• 22/23 (29.7%): nocturnal sweating (missing 51, 68.9%).

• 4/74 (5.4%): vomiting during the night only, without any associated viral or other infection.

• 5/9 (6.8%): nocturnal enuresis (missing: 65/74, 81.1%).

• 1/74 (1.4%): Ground their teeth while sleeping. This question was initially not included in the clinical history questionnaire.

c) upon awakening:

• 1/74 children (1.4%): Morning headaches (missing 73/74, 98.6%)

• 8/9 (88.9%): difficulties waking up in the morning (missing 87.8%).

d) during daytime:

• 10/15 children (66.7%) continued taking naps ( 79.7%).

• 37/74 (50%): sleepiness (missing 45.9%),

• 37/38 (97.4%): tiredness (missing 48.6%),

• 29/74 (39.2%): sunken eyes,

• 17/74 children (23%) “heavy” respiration.

8) Clinical signs of hyperactivity/hypersensitivity/concentration/learning problems:

• 8/12 children (66.7%): cried for nothing, and they were irritable or furious with their siblings without reason (missing 83.8%).

• 3/74 (4.1%): hypersensitivity (missing 95.9%),

•26/30 (35.1%) (Missing: 44/74 59.5%): hyperactivity,

• 11/14 (78.6%): concentration/learning problems (missing 60/74, 81.1%).

9) Orthodontic problems/oral disorders:

• 3/6 children (50%): Oral disorders (missing 91.9%)

• 4/9 (44.4%): orthodontic problems (missing 87.8%),

• 3/13 children (23.1%) wore braces (missing 82.4%).

10) Associated recurrent illness/digestive signs/gastroesophageal reflux:

• 25/28 children (89.3%): They were recurrently sick (missing 62.2%).

• 33/44 (75%) (missing: 40.5%): digestive signs (recurrent constipation/diarrhoea, alternating constipation diarrhoea, recurrent abdominal pain).

• 16/21 (76.2%): Gastroesophageal reflux in infancy (missing 71.6%).

11) Associated reported allergic signs:

• 66/70 (94.3 %): atopic profile (eczema, recurrent rhinitis/conjunctivitis, asthma) (missing 5.4%).

• 48/50 (96 %): recurrent rhinitis with no associated fever ( 24/74, 32.4%).

• 10/11 (90.9 %) recurrent rhinitis with no associated fever since early infancy (missing 85.1%)

• 21/25 (84%) recurrent otitis (missing 66.2%).

• 21/28 (75 %) eczema (missing 62.2%).

• 7/24 (29.2 %): recurrent conjunctivitis (missing 67.6%).

• 66/73 (90.4 %): asthma signs (missing: 1.4%).

• 52/55 (94.5 %): recurrent cough (missing 25.7%).

• 25/33 (75.8 %): Asthma on the effort (missing 55.4%).

• 12/17 (70.6 %): recurrent laryngitis (missing 77 %).

12) Under AT or ED on the night of the overnight PG:

• 26/73 (35.6 %) under AT (missing 1.4%).

• 11/73 (15.1 %) under ED (missing 1.4%).

• 32/73 (43.8 %) under AT or ED (missing 1.4%).

13) RA and IgEFA/NIgEFA:

• 51/68 (71 %) had an allergy (RA/FA) (missing: 8.1%).

• IgEFA was reported in only 6/74 (8.1%): shrimp (1 case, 1.4%), hazelnut (3 cases, 4.1%), cow’s milk (1 case, 1.4%), and hen’s egg (1 case, 1.4%).

• 50/74 (67.56%) reported eczema or digestive signs and were therefore tested for NIgEFA.

• 31/50 (62%) had a NIgEFA (missing: 24/74 32.4%). All six (6) children with IgEFA also had concomitant NIgEFA.

• More than half of the children (40/70, 57.1 %) had an RA (missing 5.4 %), and nearly half of them (36/69, 52.2 %) had a dust mites’ allergy (MA) (missing 6.8 %).

• 5/68 (7.35 %) in between the children tested for Alternaria Alternata (AA) were sensitized to AA (missing 44.6%).

• 50/74 were tested for both RA and NIgEFA (milk, wheat, +/- soymilk), &

• 17/50 (34%) suffered both a NIgEFA and a RA (MA: 14/50, 28%) (missing: 24/74, 32.4%).

14) SLIT was initiated in five children who had a significant amelioration of their allergies and were overweight (12, 14, 19-21).

**Analysis of results of PG recordings**

**Total analysis time (TS) (minutes) in PG recordings**: M = 657.8 minutes, SD= 102,195 (minimum= 352 min, maximum= 900 min).

**Total sleep time (TST) (minutes) in PG recordings:** M= 517.05 minutes, (8 h 37 min) SD= 103,877 (minimum= 193 min, maximum= 709 min).

**Correlation results**

- We found a strong correlation between:
- 1) BMI and AHI, *r* (73) = .619, *p* < .001, CI [.453, .753],
- 2) AHI and ODI, *r* (71) = .577, *p* < .001, CI [0.397, .714],
- 3) AHI and RDI, *r* (49) = .621, *p*< .001, CI [0.411, 0.768],
- 4) SF and RDI, *r* (50) = .763, *p*< .001, CI [.616, .859],
- 5) SF and SFVO, *r* (50) = .821, *p* <.001, CI [0.703, 0.895],
- 6) SFVO and RDI, *r* (50) = .923, *p* <.001, CI [0.868, 0.956],
- 7) SFVO and RE, *r* (50) = .567, *p* <.001, CI [0.343, 0.730],
- 8) RE and RDI, *r* (50) = .625, *p* <.001, CI [0.420, 0.770],
- 9) RDI and ODI, *r* (49) = .607, *p* <.001, CI [0.392, 0.759].
- We found a moderate to strong correlation between:
- 1) BMI and RE, *r* (50) = .457, *p*< .001, CI [0.204, 0.652],
- 2) SFVO and ODI, *r* (49) = .436, *p* .002, CI [0.177, 0.639],
- 3) SFVO and AHI, *r* (49) = .423, *p* .002, CI [0.161, 0.629],
- 4) SF and AHI, *r* (49) = .305, *p* .033, CI [0.026, 0.540].
- We found a moderate correlation between:
- 1) SF and ODI, *r* (49) = .347, *p* .015, CI [.073, .572],
- 2) SF and RE, *r* (50) = .330, *p* .019, CI [.056, .557],
- 3) AHI and RE, *r* (49) = .248, *p* .085, CI [-.035, .495],

IgEFA correlated to:

1) AHI, r (73) = .332, *p* .004, 2) SF, r (50) = -.289, p .042, 3) increased AHI>6,8 r (73) =.265, *p* .024

Coexistence of MANIgE as compared to no MANIgE correlated to:

1) AHI r (49) = 290, *p* .043, 2) RE r (33) = .396, *p* .022

2) RDI r (33) = .471, *p* .006, 4) SF r (33) = .355, *p* .043

3) ODI r (49) = .397, *p* .005, 6) SFVO r (33) = .486, *p* .004

4) AHI>6,8 r (49) =.270, (*p* .060) 8) RE>22% r (33) = .303, *p* .086

5) RE>28% r (33) = .433, *p* .012, 10) RE>20% r (33) = .387, *p* .026

Coexistence of RANIgE as compared to no RANIgE correlated to:

1) AHI r (49) = 276, *p* .055, 2) RE r (33) = .523, *p* .002

2) RDI r (33) = .409, *p* .018, 4) ODI r (49) = .416, *p* .027

3) SFVO r (33) = .427, *p* .013

4) increased AHI>6,8 r (49) =.312, (*p* .029) 7) increased RE>22% r (33) = .371, *p* .034

5) increased RE>28% r (33) = .527, *p* .002, 9) increased RE>20% r (33) = .449, *p* .009

**ANOVA** revealed a significant association between the groups (**Supplementary Table 1. Online Repository)**:

- A medium to large effect on AHI, of the:

1. AT (Eta:, 317 Eta squared: 100) (*p* .007) (F 7,793).
2. NIgEFA (Eta: 354 Eta-squared: 125 (*p* .013) (F 6,716).
3. RANIgE (Eta: 276, Eta squared: 076) in a marginally significant manner (*p* .055) (F 3,882).

- a medium effect on AHI of the:

1. MANIgE (Eta: 326, eta squared: 084) (*p* .043) (F 4,331).

- a medium to large effect on AHI of the:

1. IgEFA and NIgEFA (Eta:, 332, Eta squared: 110) (*p* .004) (F 8,768).

- a large effect on RE of the:

1. RANIgE (Eta:, 523; Eta squared: 274) (*p* .002) (F 11,694).
2. MANIgE (Eta: 396, Eta squared: 157) (*p* =.022) (F 5,777).

- a medium effect of :

1. The co-existence of IgEFA and NIgEFA (Eta:0,289, Eta squared: 0,084) in SF (*p* .042) (F 4,376).

**t-test results (Supplementary Table 1. Online Repository)**

**AHI**

• Mean (M) AHI was found to be lower in children who followed AT (M = 3.0207, SD = 1.964) than in those who did not (M = 6.821 cm, SD = 7.646).

• Children who were not under AT the time of the PG experienced significantly higher (M) AHI than children who were under AT the time of the overnight PG, t(70) = 3.015, p = .004.

• The effect size for this analysis (d = .580) exceeded Cohen’s (1988) convention for medium effects (d = .50).

• (M) AHI mean and median values of AHI significantly varied between children under AT or ED (M = 3.237, SD = 2.147) and no AT or ED (M = 7.327, SD = 8.051) (Figures 2a, 2b).

• Children who were under AT or ED upon PG experienced significantly lower (M) AHI than children who were not under AT or ED at the time of overnight PG, t (70) = -3.079, p = .004.

• The effect size for this analysis (d = .662) was found to exceed Cohen’s (1988) convention for a medium effect (d = .50).

• (M) AHI significantly varied between children with NIgEFA (M = 7.49, SD = 8.095) and those without NIgEFA (M = 3.073, SD = 2.182).

• Children who did not undergo NIgEFA upon PG experienced significantly lower (M) AHI than children who experienced NIgE upon PG, t (47) = 2.317, p = .008.

• The effect size for this analysis (d = .679) was found to exceed Cohen’s (1988) convention for a medium effect (d = .50).

• AHI × RANIgE: ANOVA revealed a marginally significant association; however, the t-test did not reveal a significant association, indicating the existence of more than two groups.

• (M) AHI non significantly different between children with coexistence of RANIgE (M = 8.443, SD = 8.362) and no RANIgE (M = 4.484, SD = 5.578) (Figure s3a, 3b, 3c).

• Children who did not suffer RANIgE upon PG did not experience non significantly lower (M) AHI than children who suffered RANIgE upon PG, t (47) = 1.970, p = .100.

• The effect size for this analysis (d = .600) exceeded Cohen’s (1988) convention for medium effects (d = .50).

• **RE**

• (M) RE significantly varied between children under AT or ED (M = 15.170 cm, SD = 12.916) and no AT or ED (M = 27.027 cm, SD = 16.305) (Figures 2a,2b, 4a, 4b).

• Children who were under AT or AE upon PG experienced lower RE levels than those who were not under AT or AE upon PG, and this was statistically significant (t (48) = 2,728, p = .009).

• The effect size for this analysis (d = .788) was found to approximate Cohen’s (1988) convention for a large effect (d = .80).

• RE significantly varied between children with RANIgE (M = 33.358, SD = 20.018) and without RANIgE (M = 15.4, SD = 10.296) (Figures 4a, 4b, 5a, 5b).

• Children who did not experience RANIgE upon PG experienced significantly lower (M) RE than children who experienced RANIgE upon PG (t (32) = 2.896, p = .002.

• The effect size for this analysis (d = 1.237) was found to exceed Cohen’s (1988) convention for a large effect (d = .80).

An independent-samples t-test was conducted, and a one-way ANOVA revealed a significant difference in the following groups:

1) AHI in RANIgE (p .029) (df 47, F 5.088, t 2.256) (Cohen’s D, 699, Hedges’, 688) (eta-squared: 098).

2) AHI in NIgEFA (p .013) (df 47, F 6,716, t 2,592) (Cohen’s D, 748, Hedges’, 736) (eta-squared: 125).

3) AHI in co-existence IgEFA and NIgEFA (p .004) (df 71, F 8,768 t 2,961) (Cohen’s D 1,372, Hedges’ 1,358) (eta-squared: 110).

4) SFVO in Allergy (p .01) (df 42 F 7.228, t 2.689) (Cohen’s D —,888, Hedges’ —,872) (Eta-squared: 147).

5) SF for MANIgE (p .071) (df 26, F 3.543, t 1.882) (Cohen’s D, 742, Hedges’, 721) (Eta-squared: 120).

6) RDI for RANIgE (p .018) (df 31, F 6.228, t 2.496) (Cohen’s D .903, Hedges’, 881) (Eta-squared: 167).

7) RDI in Allergy (p .031) (df 42, F 5.002, t 2,237) (Cohen’s D .739, Hedges’, 726) (Eta-squared: 106).

8) RE in the Atopic Profile (p =.047) (df 46, F 4.163, t 2.040) (Cohen’s D 1,217, Hedges’ 1,197) (Eta-squared, 083).

**Cross tabs (Table 1)**

• A Χ^2^ test of independence revealed a significant association between obesity/overweight and RANIgE, Χ2(1, N = 50) = 7,219, p =

.012. RANIgE had a medium effect on obesity (Somer’s D, 450, Eta and Phi, Cramer’s V, Kendall’s tau-b, 380, Kendall’s

tau-c, 288).

• **RANIgE** had a:

medium effect upon increase of the:

• AHI>6.8 n/h [Eta, 312; Somer’s D (RANIgE-dependent): 340; Phi and Cramer’s V; and Kendall’s tau-b, 312]

• RE>20% [Eta, 449, Somer’s D (RANIgE dependent): 433, Phi and Cramer’s V, and Kendall’s tau-b, 449]

• RE>22% [Eta, 371, Somer’s D (RANIgE dependent): 361, Phi and Cramer’s V, and Kendall’s tau-b, 371]

and

• A large effect upon increasing RE>28% (Eta .527, Somer’s D .569, Phi and Cramer’s V, and Kendall’s tau-b .527).

**The number needed (NN) to be exposed** to RANIGE to harm (having RE >20% during sleep) was 2.2 (In Figure 1 is visualized the clustered box plot of the RE by RANIgE by obesity group).

• The NN to be exposed to RANIGE ( AHI/= 6.8n/h during sleep) was 2.9.

• The NN to be exposed to RE =20% during sleep to harm (become obese/overweight) was 3.7.

• The NN to be exposed to RANIGE to harm (becoming obese/overweight) was 3,1. On average, 3,1 children would have to be

• Exposure to the experimental situation (RANIgE instead of no RANIgE) for one additional child to obtain the study outcome

• (obesity/overweight).

• AT or AE had a medium effect upon decreasing RE<22% [Eta, 333, Somer’s D(ATED-dependent) -,333, Phi and Kendall’s tau-

b and Cramer’s V —,333]

• The effect of AT or AE was small upon decreasing AHI <6.8n/h (Eta, 252, Somer’s D —,309, Phi and Kendall’s tau-b and Cramer’s

V —,252), but it was larger than the effect of AT alone [Eta, 243, Somer’s D(AT dependent) —,288, Phi and Kendall’s tau-b, and

Cramer’s V —,243].

• AT or AE had a small to medium effect on obesity/overweight [Eta .339, Somer’s D (ATED-dependent) -.440, Phi and Kendall’s

tau-b -.339 and Cramer’s V .339]

• The **Number Needed to Treat** (**NNT)** (with AT or AE) to have the outcome (AHI<6.8n/h) was 4.9.

• The NNT (with AT or AE) to achieve the outcome (RE<22%) was 3.

• The NNT (with AT or AE) to avoid the outcome (obesity/overweight) was 3.8.

**ROC curves**

• ROC analysis (BMI adjusted for age and sex) indicated the following.

• a) RE showed moderate accuracy (AUC =0.769, SE=.088, CI= .597 .942, p=.017) in predict obesity (BMI≥ 95th percentile) and overweight (BMI >85th percentile and <95th percentile) versus normal weight (BMI 5th-85th percentile). An RE greater than or equal to 28.4% would be related to a sensitivity of 62.5% and to a specificity of 21.4%, which suggests reliance on it would produce a moderate number of false positives (Figure 2).

• b) AHI showed moderate accuracy (less than the RE) (AUC =0.768, SE=.094, CI= .583 .952, p=.004) in predict obesity (BMI≥ 95th percentile) and overweight (BMI >85th percentile and <95th percentile) versus normal weight (BMI 5th-85th percentile). BMI adjusted for age and sex. An AHI greater than or equal to 5.05% would be related to a sensitivity of 66.7% and to a specificity of 27.9% which suggests reliance on it would produce a moderate number of false positives (27.9%) (Suppl. Figure 5. Online Repository).

c) RANIgE showed moderate accuracy (AUC = 0.725, SE =.094, CI = .542 .908, p =.029) to predict obesity (BMI≥ 95th percentile) and overweight (BMI >85th percentile and <95th percentile) versus normal weight (BMI 5th-85th percentile (Suppl. Figure 6 and Suppl. Table 2. Online Repository).

• d) The use of AT or ED alone did not show a discrimination value (AUC = 0.295, SE =.074, CI = .151 .440, p = .031) to predict obesity (BMI≥ 95th percentile) versus normal weight (BMI 5th-85th percentile) and overweight (BMI >85th percentile and <95th percentile) (Suppl. Figure 9). Online Repository).

e) The use of RANIgE showed a moderate discrimination value (AUC = 0.755, SE =.075, CI = .608 .902, p = .003) for predict BMI (Suppl. Figure 7). Online Repository).

• f) The use of RANIgE showed a moderate discrimination value (AUC = 0.788, SE =.088, CI = .615 .960, p = .007) in predict RE (Suppl. Figure 8. Online Repository).

**Multiple UGLM are reported in Suppl. Tables 3-18 and 26-32**

**Multiple UGLM are reported in Suppl. Tables 3-18 and 26-32**

**A UGLM** was estimated to investigate whether AT, AE, and RANIgE levels predict RE. Tests to see if the data met the assumption of collinearity indicated that multicollinearity was not a concern *(RANIgE, Tolerance = .981, VIF = 1.020; AT or ED, Tolerance = .981, VIF = 1.020) (****Suppl. Table 3.*** **Online Repository).** *Coefficients of* a UGLM were estimated to investigate whether AT, AE, and RANIgE predict RE*).*

The overall model was statistically significant (F (2, 32) = 14,293, *p* <.001). Together, the predictors accounted for a moderate amount of variance in the outcome (R^2^= .488, Adjusted R^2^= .454. AT or AE significantly independently predicted RE, F (1,32) = 12,539, *p* <.001(Suppl. **Table 4. Online Repository*)***. In the parameter estimates, AT or AE predicted RE significantly (B = 15,602, SE = 4.406, t (1)) = 3.541, *p* < .001. We found a large effect of AT and AE on RE (Partial Eta squared: n^2^= .295, Observed Power= .929, computed using alpha=.05, CI: 6.604 – 24.601) (Suppl. **Table 5. Online Repository*)***. RANIgE significantly independently predicted RE (F (1,32) = 12.073 p .002. In the parameter estimates, RANIgE significantly predicted RE (No RANIgE B = -15,729 SE = 4.527, t (1)) = -3.475, p .002. We found a large effect of RANIgE on RE (Partial Eta squared: n^2^= .287, Observed Power= .919, computed using alpha=.05, CI: -24.975–-6.484) (**Supplementary Figure 10. Estimated Marginal Means of RE. Online Repository**).

**A UGLM** was estimated to investigate whether RANIgE and obesity versus healthy weight/overweight (***obesityVsHW/overw*** predicts RE. Tests to see if the data met the assumption of collinearity indicated that multicollinearity was not a concern (RANIgE, Tolerance = .853, VIF = 1.172; obesity and overweight versus healthy weight group, Tolerance = .853, VIF = 1.172) (**Supplementary Table 6.Online Repository** Coefficients of a UGLM estimated to investigate whether RANIgE and ***obesityVsHW/overw*** predict RE). .

The overall model was statistically significant F (3, 32) = 7,806, p <.001. Together, the predictors accounted for a moderate amount of variance in the outcome (R2= .447, Adjusted R2= .390. RANIgE significantly independently predicted RE, F (1,32) = 9,940 p .004 (**Suppl. Table 7. Online Repository**. Tests of Between-Subjects Effects of a univariate GLM which was estimated to investigate whether RANIgE and obesityVsHW/overw predict RE.). In the parameter estimates, the absence of RANIgE significantly predicted RE (B = -39,575, SE = 14.643, t (1)) = -2.703, p .011 (**Suppl. Table 8. Online Repository**. Parameter Estimates of a univariate GLM estimated to investigate whether RANIgE ***and obesityVsHW/overw*** predict RE.). The lines in the profile plot intersect, which indicates that there is an interaction between RANIgE, obesity group, and RE (**Supplementary Figure 13. Online Repository**).

However, when grouping obesity with overweight versus healthy weight, there was a marginal significance for the obesity and overweight groups versus healthy weight (***obesity/overwVsHW***), and the R square increased to .455. This GLM is described in more detail below.

**A UGLM** was estimated to investigate whether AT or AE and obesity predicted AHI. Tests to determine if the data met the assumption of collinearity indicated that multicollinearity was not a concern (AT or AE, Tolerance = .922, VIF = 1.084; ***obesity/overwVsHW***, tolerance = .894, VIF = 1.118) (**Suppl. Table 13**. Coefficients^’^ Online Repository).

The overall model was statistically significant (F (3, 71) = 18,895, p <.001). Together, the predictors accounted for a moderate amount of variance in the outcome (R2= .455, Adjusted R2= .431. AT or AE significantly independently predicted AHI, F (1,71) = 8,611 p .005. A significant interaction was found between AT or AE and ***obesity/overw VsHW***, F (1,71) = 6,777, p .011. **obesity/overwVsHW** marginally significantly predicted AHI, F (1,71) = 3,107 p .082 (**Suppl. Table 14. Tests of Between-Subjects Effects. Online Repository**). In the parameter estimates, the lack of AT or AE significantly predicted AHI (B = 14,536, SE = 5.096, t (1)) = 2.852, p .006 (Suppl. Table 15). Online Repository Parameter Estimates). The lines in the profile plot intersect, indicating that there is an interaction between AT or AE, ***obesity/overwVsHW*** and AHI **(Suppl. Figure 14. Online Repository).**

**However, when controlling for** the assumption of homogeneity of regression slopes, we see that there is a statistically significant interaction between RANIgE * AsthmaTreatmentAllergen Eviction* AHI (p <.001(Suppl. **Tables 27. Tests of Between-Subjects Effects, Suppl. Table 28. *Parameter Estimates.* Online Repository*)***. In this case, the assumption of homogeneity of the regression slopes was violated. The more this assumption is violated, the more prone it is to fail to reject the null hypotheses (Type II error, or “false negative error”). This results in a significant relationship,  but is indicated as non-significant; therefore, we do not reject the null hypotheses.  In our case, the groups differ significantly on the covariate (AHI) (thus an interaction) then adding the covariate into the analysis will not “control for” or “balance out” those differences across the groups. The significant interaction between RANIgE and AT or ED in the covariance of AHI is at the origin of failing to show significance in the relationship between the different independent variables and the dependent variable.

**A Poisson regression analysis** was used to investigate whether AT, ED, and RE could predict BMI.

The likelihood ratio Χ^2^ test (5,982) indicated that the full model was a marginally significant improvement in fit over a null (no predictors) model (p =.050) (Suppl. **Table 19**. **Online Repository*)***. Goodness of fit criteria: Deviance/df = 1.082, Log Likelihood = -30.063, BIC = 67.321, AIC = 69.555, CAIC = 70.321 (**Suppl. Table 20**. **Online Repository*)***.

RE significantly predicted BMI (B= .009, S.E.=.0035, *p* .014) (**Suppl. Table 21**. **Online Repository*)***. For every unit increase in RE, the predicted log count of BMI increased by .009. ATED was not a significant predictor of BMI (B= -.074, S.E.=.2025, p .715). For every one-unit increase in ATED, the predicted log count of BMI decreased by -.074.

A **binary logistic model of regression** a GLM was performed to ascertain the effects of RE and ATED on the likelihood that participants would develop obesity/overwVsHW. A preliminary analysis suggested that the assumption of multicollinearity was met *(ATED, Tolerance = .866, VIF = 1.155; RE, Tolerance = .866, VIF = 1.155) (****Suppl. Table 22****.* **Online Repository***).* The overall model was statistically significant, Likelihood Ratio x^2^ (2, N= 50) = 8.000, *p*= .018, suggesting that it could distinguish between those with an obesity/overweight and those without an obesity/overweight diagnosis (**Suppl. Table 23. Online Repository*.***). The predictor variable, RE, was tested by theoretical deduction to verify that there was no violation of the assumption of logit linearity. The predictor variable RE in the binary logistic analysis was found to contribute to the model. The unstandardized beta weight (B) for the Constant; B= 4.002, SE=1.2468, Wald= 10.301, p .001. The unstandardized B for the predictor variable (RE) was B = (-.053), SE= .0261, Wald=4.12, *p* =.042. *The results of the binary regression GLM indicated that, all else being equal, subjects having lower RE had fewer odds of having the outcome “obesity” than subjects having increased RE (OR = 4,120; 95% CI: -.104 to -.002; p = 0.042)* (**Suppl. Table 24.** **Online Repository**)*. Goodness of fit: BIC: 47.703, AIC: 41.967, CAIC: 50.703 (****Suppl. Table 25****.* **Online Repository***).*

**A UGLM** was estimated to investigate whether AT or ED and RANIgE predicted BMI, while controlling AHI and RE as covariates. Tests to determine if the data met the assumptions of Levene’s test of equality of error variances and tests for heteroskedasticity indicated that the assumptions were not violated *(****Suppl. Table 29****.* *Levene’s Test of equality of error variances and* ***Suppl. Table 30.*Online Repository** *F Test for Heteroskedasticity****.****).*

The overall model was statistically significant, F (6, 23) = 6,336, *p* .001. Together, the predictors accounted for a moderate amount of variance in the outcome (R^2^= .691, Adjusted R^2^= .582. In the table of tests of between-subject effects, we see that there is a significant interaction between AT or ED * RANIgE * AHI (**Suppl. Table 31.Online Repository.** *Tests of Between-Subjects Effects* of a UGLM which was estimated to investigate whether RANIgE and AT or ED predict BMI.***)***. In the parameter estimates, we obtained more information, as we see that there is a significant interaction (*p* .001) specifically between the absence of AT or ED and the co-existence of RANIgE to influence AHI [AT or ED= 0] * [RANIgE=1] * AHI (**Suppl. Table 32.Online Repository***. Parameter Estimates.***)**. The lines in the profile plot intersect, which indicates that there is an interaction between RANIgE, AT or ED, and BMI while evaluating AHI and RE as covariates (**Figure 4**).

The profile plot of BMI according to both AT or ED and RANIgE is shown in Figure 4 along with the rest of the profile plots already presented. The GLMM that we report afterward helped us to create the path analysis that we present in the next part of our statistical analysis.

- ***A Generalized Linear Mixed Model (GLMM):*** Due to the skewed distribution of BMI, a GLMM was created and the *positive effects of AHI/RE/No ATED and a negative effect of the absence of RANIgE on BMI (Figure* ***5****, Suppl.Fig.****16a-b****, Suppl. Tables* ***33-44****). The Fixed effects appear to be strong with narrow Confidence Intervals (CIs). The variation between subjects does not seem to be considerable.*

***Path analysis with serial mediation (Suppl. Tables 45-51)***

**Ingredients**

**Indirect effect:** The impact of RANIgE and AT or AE (IVs) on BMI (DV) through the mediating variables (AHI × RE) (a × b)

**Total effect:** Impact of IV on DV without involvement of mediator (c)

**Direct Effect:** The Impact of IV on DV in the presence of mediator (c)

**Type of mediation:** Partial or Full

**Full mediation:** This form of mediation exists if predictor (IV) exerts its total influence through the mediating variable.

**Partial mediation:** This form of mediation exists if the predictor variable (IV) exerts influence via the mediator. It also exerts some influence directly on the outcome variable and not via the mediating variable (MV). Thus, the influence is extended in DV through both IV and MV. Thus, paths a*b (indirect effect) and c’ (direct effect) are both significant.

A moderation effect means that the effect of a continuous independent variable (X; e.g., AHI or RE) on a continuous dependent variable (Y; e.g., BMI) depends on the moderator’s level (M: categorical or continuous. M, e.g., RANIgE or AT or ED).

Mediation analysis focuses on the causal pathway between the independent variable (IV) and dependent variable (DV) through an intervening variable. The moderation analysis concentrates on how a moderator variable affects the relationship between IV and DV.

A mediation analysis was performed to assess the mediating role of RE and AHI in the relationship between RANIgE/AT or ED and BMI.

The model below is a recursive path model.

**Fit of the model:**

The indices of model-data fit evaluated were CMIN, RMSEA, SRMR, CFI, and AIC. A perfect model fit is indicated by SRMR = 0, as in our case(23). CFI values greater than .90, indicates adequate fit, ((23), as has been the case in our model.

**Bollen-Stine Bootstrap (Default model)**

The model fit better in, 1409 bootstrap samples.

It fit about equally well in 0 bootstrap samples.

It fit worse or failed to fit in 3591 bootstrap samples.

Similarly, in the regression analysis, significant parameter estimates were RANIgE on RE (p = .002), AT or AE on RE (p =0.006), AT or AE on AHI (p = 0.025), RANIgE on BMI (p = .022) and AT or AE on BMI (p= .021). The marginally significant parameter estimate was RANIgE for AHI (p = 0.082).

Of the eight direct effects, six are statistically significant. There was a negative direct effect of AT or AE on BMI (-.06), RE (-.38), and AHI (-.30). There were positive direct effects between RANIgE and RE (.459), RANIgE and AHI (.323), RANIgE and BMI (.163), RE and BMI (.296), and AHI and BMI (.395). For the indirect effects, there was a negative effect for AT or AE → RE+AHI → BMI (-.230) and a positive effect for RANIgE → RE+AHI → BMI (.263), both of which were not statistically significant.

**Standardized Estimates (Table 2):** When examining standardized estimates, we can see the relationships expressed in terms of standard deviations (SDs).

If RANIgE increased by one SD, while AHI and RE were held constant, BMI would be expected to increase by 0.426 SD. If AHI increased by one SD, while RANIgE, RE, and ATED were held constant, BMI would be expected to increase by 0,39 SD. If RE increased by one SD, while AHI, ATED, and RANIgE remained constant, BMI would be expected to increase 0,29 SD. If ATED increased by one SD, while AHI, RE, and RANIgE remained constant, BMI would be expected to decrease by 0,286 standard deviations.

Based on an estimated total effect of RANIgE on BMI of .426, we can see that if RANIgE was increased by one SD without holding AHI, RE, and ATED constant, then BMI would increase .426 SD.

Based on an estimated total effect of ATED on BMI of -.286, we can see that if ATED was increased by one SD without holding AHI and RE and ATED constant, then BMI would decrease by-.286 SD.

**Standardized indirect effects.**

We then performed user-specific estimands (Supplementary Table **51**. Online Repository) to calculate the specific indirect effects of RANIgE to AHI on BMI, RANIgE to RE on BMI, AT, or AE on AHI on BMI, and AT or AE on RE on BMI. We found that the specific indirect effect of AT or AE on BMI was not significant (p.212).

We also found a non-significant indirect effect of RANIgE level on BMI (β= .263, t= 1.59, p= .155). However, the total (direct and indirect) effect of RANIgE on BMI, with the inclusion of the mediator, was significant (β=.426 t=2.85 p= .022). That is, both the direct (unmediated) and indirect (mediated) effects of RANIgE on BMI. When RANIgE_IV was increased by 1, BMI_DV increased by .426. This indicates that RE mediates the relationship between RANIgE and BMI.

**Unstandardized Estimates (Suppl.Table 50):**

The effects of RANIgE on BMI include both direct and indirect effects. The direct effect of RANIgE on BMI predicts that if one were to have a RANIgE 100 more than the mean, and yet had an average AHI and RE, BMI would be 14% higher than the mean.

On the other hand, the total effect of RANIgE on BMI was 3.749, which indicates that if one increases RANIgE 100 times and allows AHI and RE to vary as it naturally would, there would be a net increase in BMI of 37%.

Increasing AHI by one unit while holding all other parameters constant would cause a BMI increase of 0,432%.

Increasing RE by one unit while holding all other parameters constant would cause a BMI increase of 0.08%.

Increasing RANIgE by one unit will increase AHI by 2,59 n/h and RE by 15%, while it will have a direct effect on increasing BMI by 1.43 kg. The partial coefficient (1,43) of RANIgE on BMI is the effect of RANIgE on BMI once the influence of AHI and RE are removed.

The effect of ATED on BMI also includes both direct and indirect effects. The direct effect of ATED on BMI predicts that if one were to have an ATED increased by one unit, and yet had an average AHI and RE, BMI would be 0,47 kilos lower than the mean.

Increasing the ATED by one unit will decrease AHI by 2,33 n/h and will decrease RE by 11.93%, whereas it will decrease BMI by 0.05 kg. The Partial coefficient (0,05) of ATED on BMI was the effect of ATED on BMI once the influence of AHI and RE was removed.

On the other hand, the total effect of ATED on BMI is -2.435, which indicates that if one increases ATED by one unit and allows AHI and RE to vary as it would naturally, there would be a net decrease in BMI of -2.435.
