# Supplementary material for: Allergies, asthma treatment, and eviction diet have a significant impact on the respiratory effort during sleep and the apnea-hypopnea index in children with obstructive sleep apnea-obesity/asthma association: A STROBE-compliant study
Source: Medicine (Baltimore). 2026 Feb 13;105(7):e41730. doi: 10.1097/MD.0000000000041730 (PMC12908835; doi:10.1097/MD.0000000000041730)

Suppl. Figure 1. Conceptual Model of Path Analysis with serial mediation in AMOS.

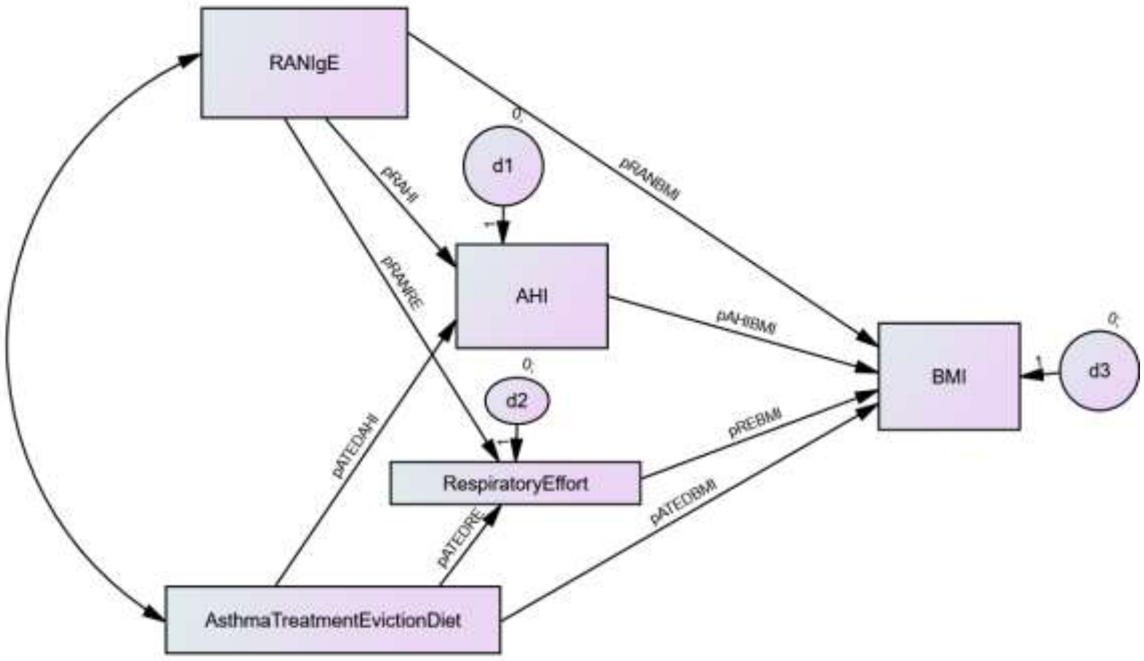

Suppl Fig.2a. Clustered Bar Mean values of Apnoea Hypopnea Index (AHI), Body Mass Index (BMI), Respiratory Effort (RE) by AsthmaTreatment or EvictionDiet (AT or ED) filtered by the co-existence of Respiratory and Non-IgE mediated allergies (RANIgE). Mean, low and high values for AHI, BMI and RE along to percentages (%) of AT or ED are shown.

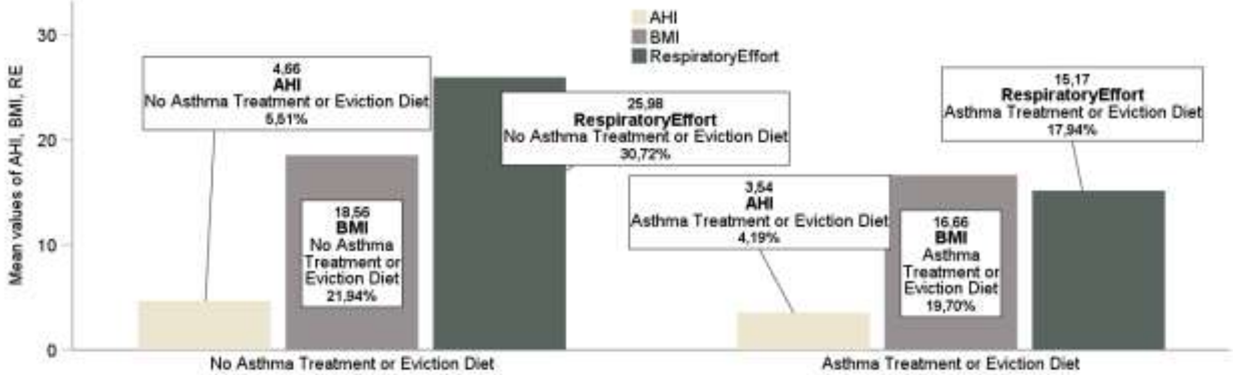

Note.This figure shows the changes of AHI, BMI, RE under AT or ED considering RANIgE. Low/high values of AHI, BMI, RE and AT or ED percentages are shown. The median AHI and RE are lower under AT or ED. AT or ED alone does not have a major impact on BMI, indicating supplementary factors mediating BMI increase to obesity.

Supplementary Figure 2b. Multiple Line Mean of Apnoea Hypopnea Index (AHI), Body Mass Index (BMI), RespiratoryEffort (RE) by Asthma Treatment or Eviction Diet (AT or ED) filtered by the co-existence of Respiratory and non-IGE mediated allergies (RANIgE variable). Mean, low and high values for AHI, BMI and RE along of percentages (%) of asthma treatment or eviction diet are also shown.

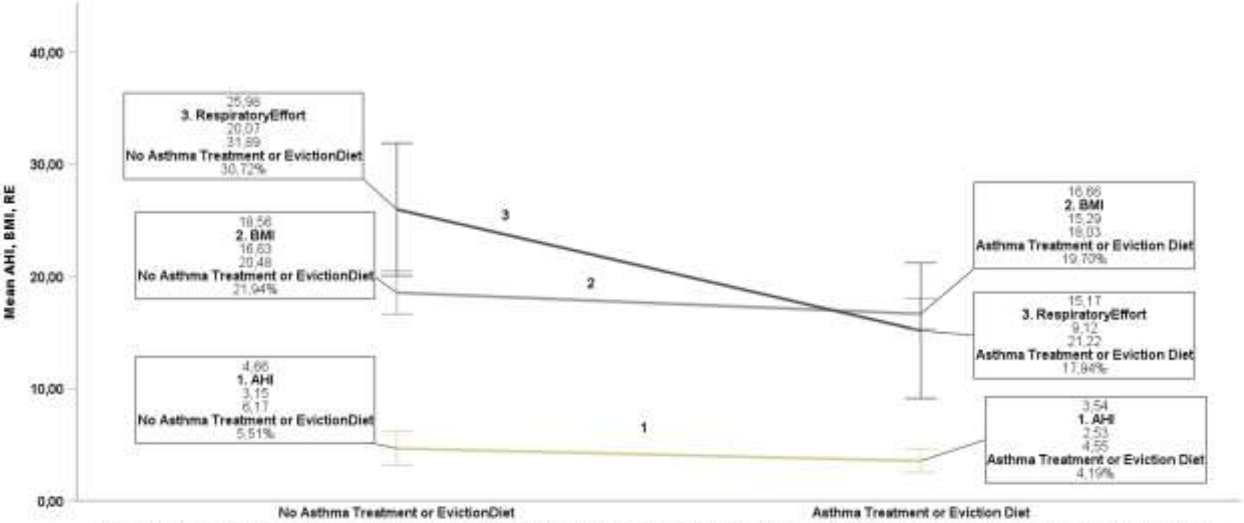

Note. This figure shows the mean values of AHI, BMI, RE under AT or ED, considering RANIgE. Low/high values of AHI, BMI, RE and percentages of AT or ED are shown. Median AHI/RE are lower under AT or ED. AT or ED alone does not have a major impact on BMI alone, indicating supplementary factors mediating upon BMI change and obesity.

Suppl.Figure 2c.Clustered Bar Mean of AHI, Median of BMI, Median of RespiratoryEffort by AsthmaTreatmentEvictionDiet while taking into consideration the co-existence of respiratory and non-IgE mediated allergies (RANIgE)

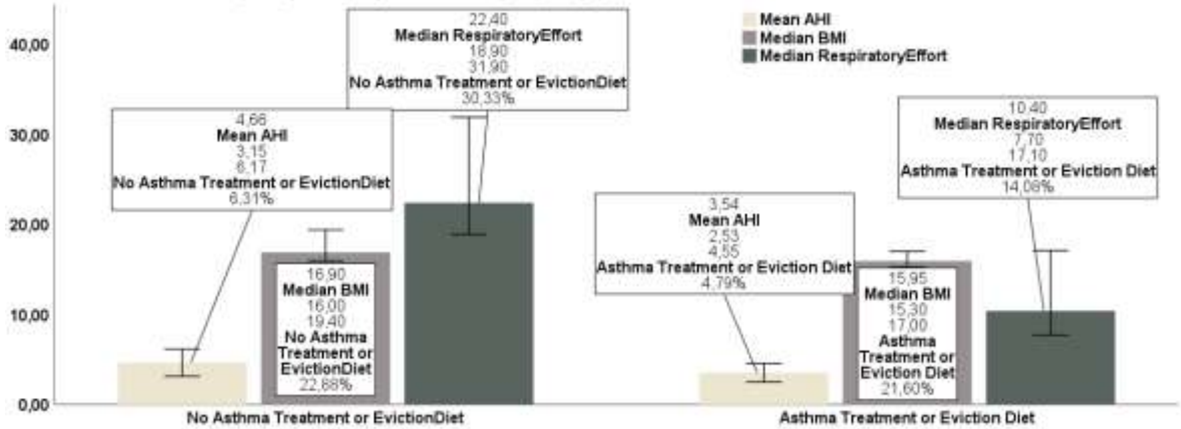

Note. This figure shows the changes of mean values of AHI, median values of BMI, RE along to already initiated Asthma treatment or eviction diet (AT or ED), while taking into consideration the co-existence of RANIgE. Low and high values of AHI, BMI, RE along to percentages of AT or ED are shown. We note that the values of AHI and RE are lower under AT or ED. AT or ED alone does not seem to have a major impact on BMI alone, indicating that there may be other factors mediating upon BMI change and obesity.

Suppl. Figure 3a. Clustered Boxplot of RespiratoryEffort by Asthma Treatment or Eviction Diet by RANIgE

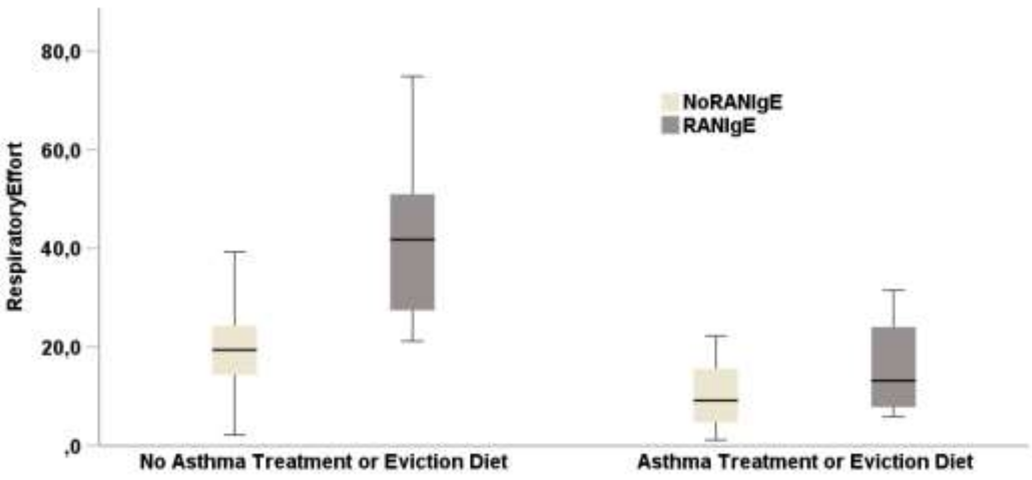

Note. This figure shows that the asthma treatment or eviction diet (AT or ED) keeps the respiratory effort during sleep(RE) in low levels in both groups of patients: those suffering (RANIgE) and those who do not suffer the co-existence of RANIgE. For children who are not under AT or ED, RE is higher for children who suffer RANIgE than in children who do not suffer RANIgE.

Suppl. Figure 3b. Clustered Boxplot of RespiratoryEffort by RANIgE by Asthma Treatment or Eviction Diet (AT or ED)

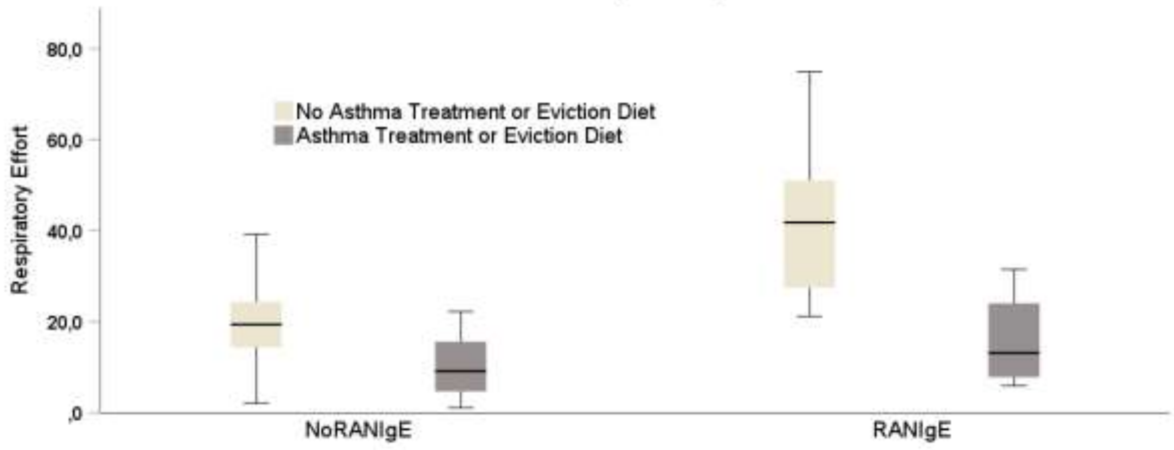

Note. This figure shows that the RANIgE keeps the respiratory effort during sleep (RE) in higher levels in both groups of patients: those who are under asthma treatment or eviction diet (AT or ED) and those who are not under AT or ED.

Supplementary Figure 3c. Clustered Bar Mean values of Apnoea Hypopnea Index (AHI), Body Mass Index (BMI), RespiratoryEffort (RE) by the co-existence of Respiratory and non-IgE mediated allergies (RANIgE variable) filtered by Asthma Treatment or Eviction Diet (AT or ED). Mean, low and high values for AHI, BMI and RE along to percentages (%) of RANIgE are also shown.

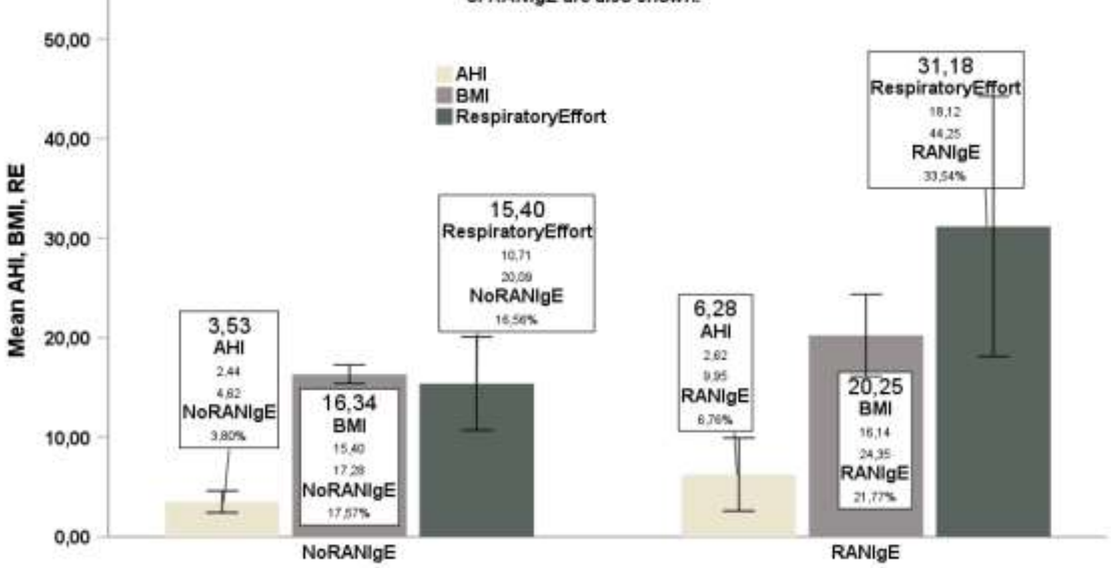

Note. This figure shows that RANIgE correlates toincreased AHI, BMI, RE compared to No RANIgE, under AT or ED. Low/high AHI, BMI, RE along to RANIgE percentages are shown. RANIgE had a major impact on RE increase and less upon AHI increase. RANIgE do not significantly impact BMI increase compared to RE increase, under AT or ED, indicating factors to mediate/moderate BMI increase.

Supplementary Figure 3d. Clustered Bar Median values of Apnoea Hypopnea Index (AHI), Body Mass Index (BMI), RespiratoryEffort (RE) by the co-existence of Respiratory and non-IgE mediated allergies (RANIgE variable) filtered by Asthma Treatment or Eviction Diet (AT or ED). Mean, low and high values for AHI, BMI and RE along to percentages (%) of RANIgE are also shown.

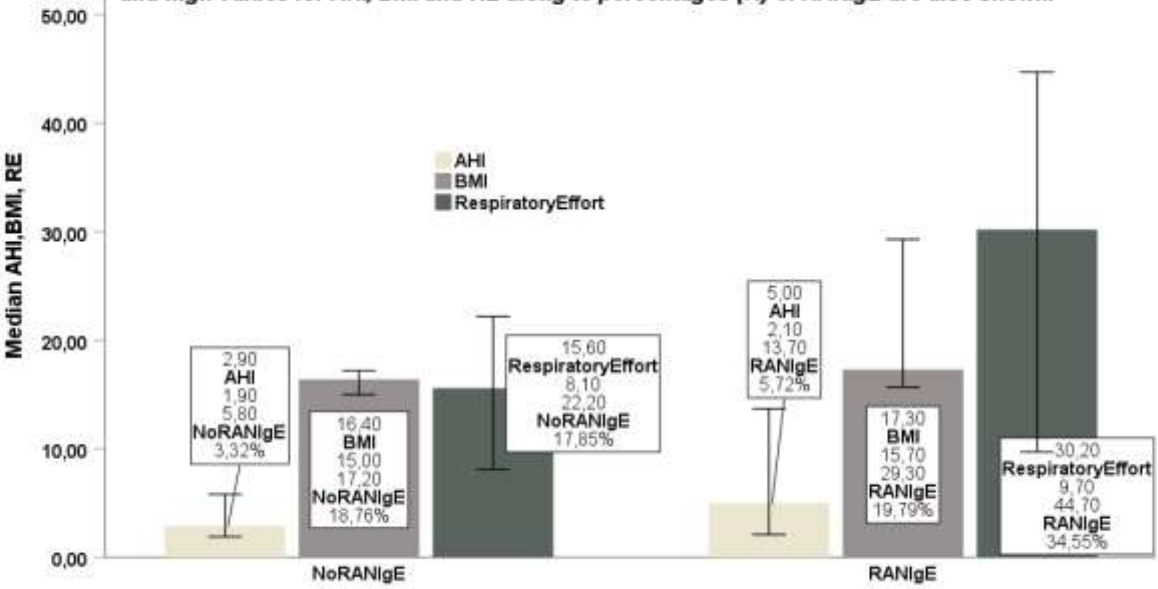

Note. This figure shows that RANIgE correlates to increased median AHI, BMI, RE compared to No RANIgE, considering AT or ED. Low/high AHI, BMI, RE and RANIgE percentages are shown. RANIgE had a major impact on RE increase and less on AHI increase. RANIgE do not have such a significant impact upon BMI increase as on the RE increase, when considering AT or ED.

Supplementary Figure 3e. Clustered Bar Median values of Apnoea Hypopnea Index (AHI), Body Mass Index (BMI), RespiratoryEffort (RE) by the co-existence of Respiratory and non-IgE mediated allergies (RANIgE variable) filtered by Asthma Treatment or Eviction Diet (AT or ED). Mean, low and high values for AHI, BMI and RE along to percentages (%) of RANIgE are also shown.

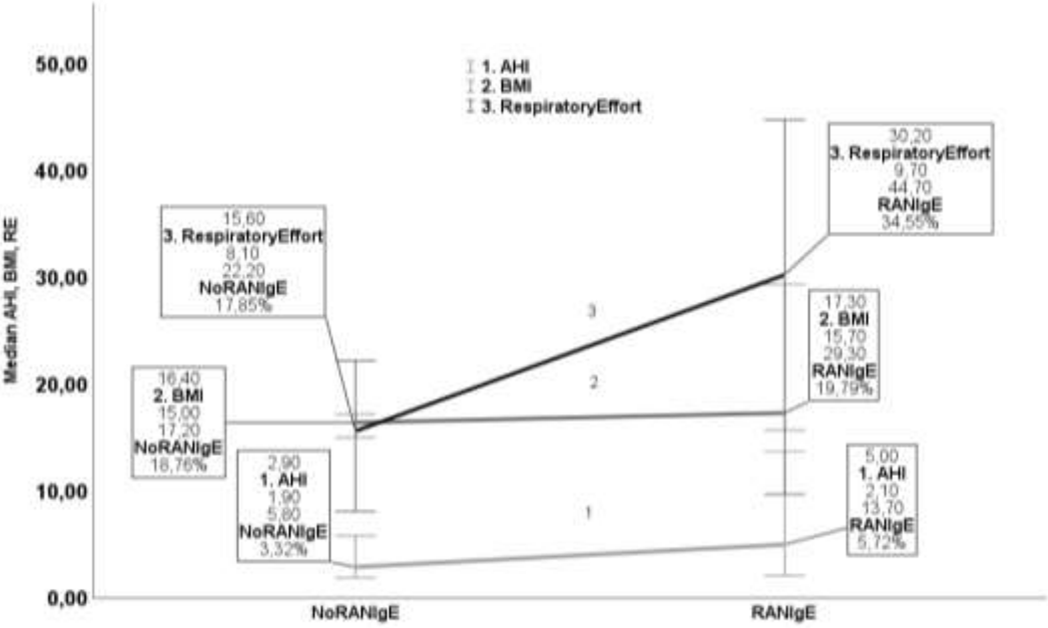

Note. This figure shows that RANIgE correlate to increased median AHI/BMI/RE compared to No RANIgE, considering for AT or ED. Low/high AHI/BMI/RE along to RANIgE percentages RANIgE are shown. RANIgE significantly impact on RE increase and less upon AHI. RANIgE do not significantly impact BMI increase as compared to RE increase, while considering for AT or ED.

Suppl. Figure 4.Clustered Boxplot of RespiratoryEffort (RE) by obesitygroup by the co-existence of respiratory and non-IgE mediated allergies (RANIgE)

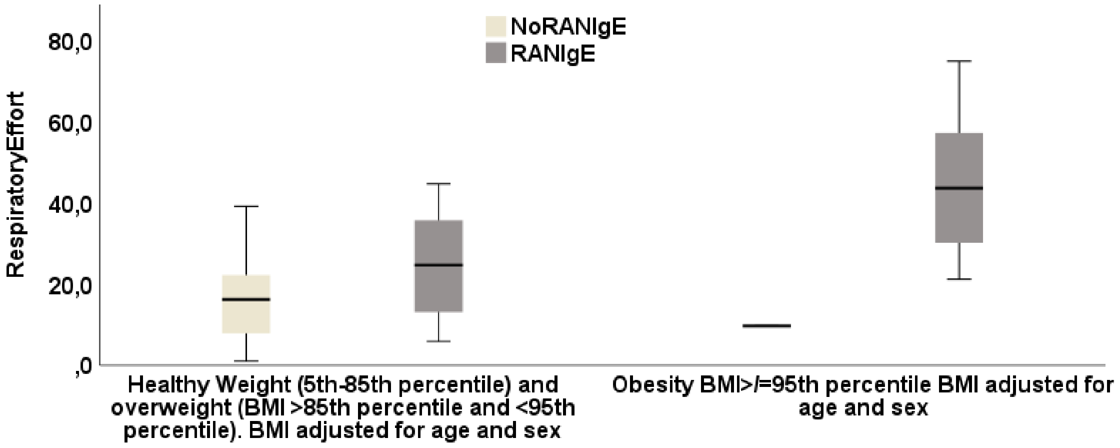

Note. This figure shows that a) the healthy weight and overweight children who do not suffer RANIgE have lower levels of RE than the healthy weight or overweight children who suffer RANIgE b) the obese children suffer predominantly RANIgE. Thus, the increased RE characterises the RANIgE patients and could favor obesity.

Suppl. Figure 4a.

Multiple Line Mean of Apnoea Hypopnea Index (AHI), Mean of Body Mass Index (BMI), Mean of Respiratory Effort (RE) by obesity versus non obesity.

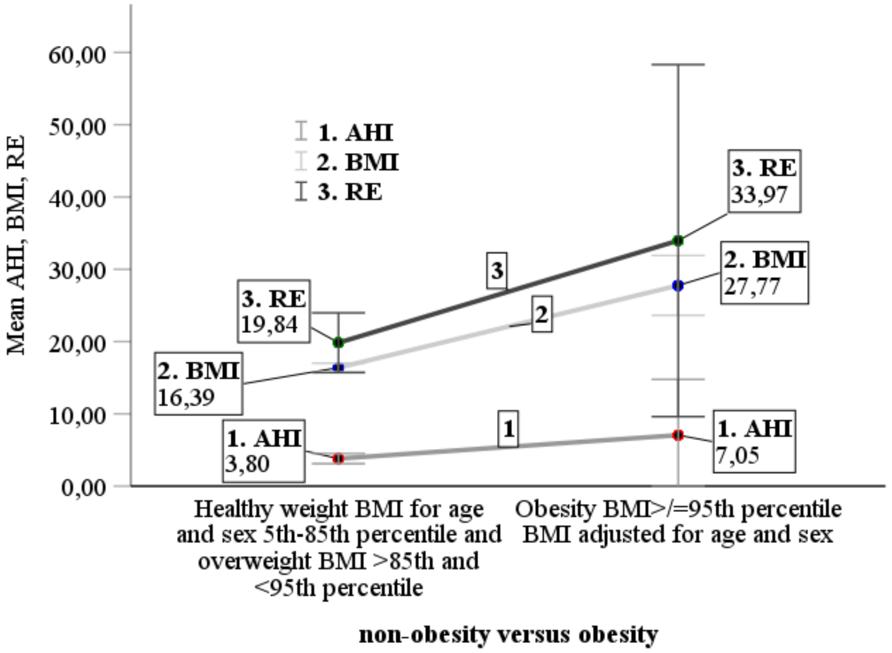

Note. This figure shows that the Respiratory Effort increase in the obese group follows the increase of BMI in a more parallel way than the increase of AHI in the same obese group.

Figure 4b.

Clustered Boxplot of RespiratoryEffort during sleep (RE) by healthy weight versus overweight and obesity through the co-existence of Respiratory and non-IgE mediated allergies (RANIgE)

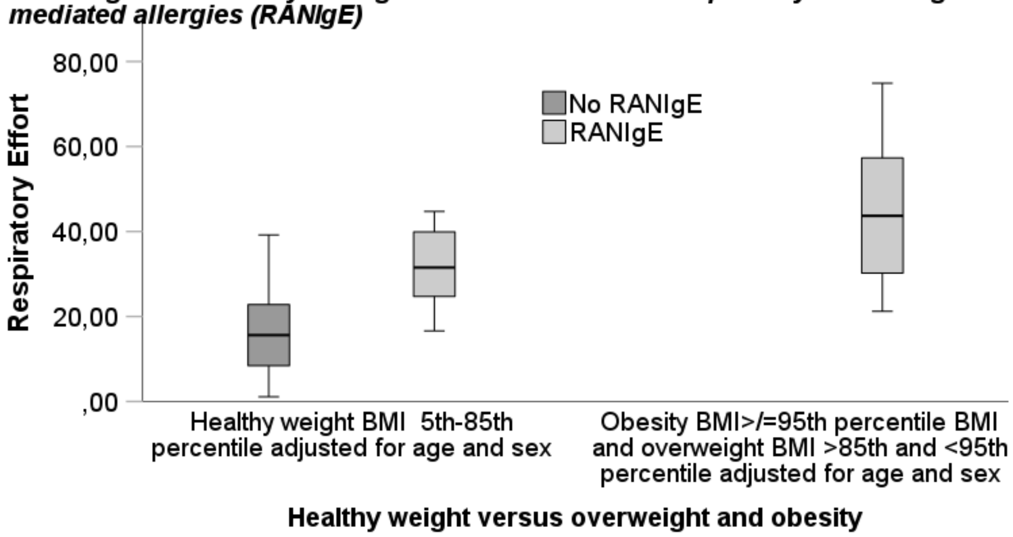

Note.This figure shows that: a) patients with RANIgE presented higher levels of RE during sleep in all groups b) patients who suffered obesity/overweight they also suffered RANIgE. The obese children presented the most increased RE. It indicates that: 1) increased RE is favored by RANIgE, 2) the co-existence of RANIgE could favor obesity/overweight through persistently increased RE due to RANIgE.

Figure 4c.

Clustered Boxplot of Respiratory Effort by obesity by Asthma Treatment or Eviction Diet (ATED)

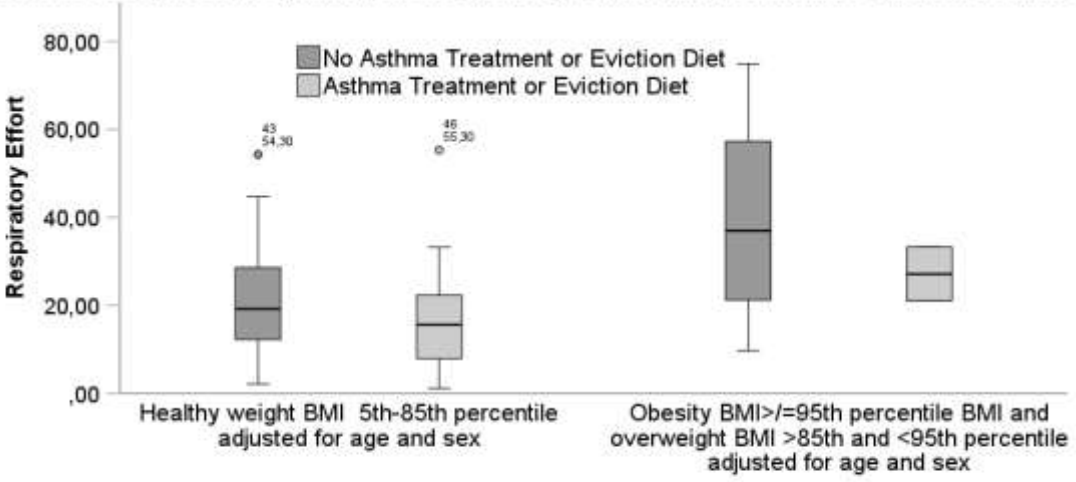

Healthy weight versus overweight and obesity

Note. This figure shows that the patients who did not follow ATED had higher RE than those who were under ATED. The majority of obese did not follow ATED and had more increased levels of RE than the obese who followed ATED. The obese had higher levels of RE than the healthy weight patients with or without ATED.

Figure 4d.

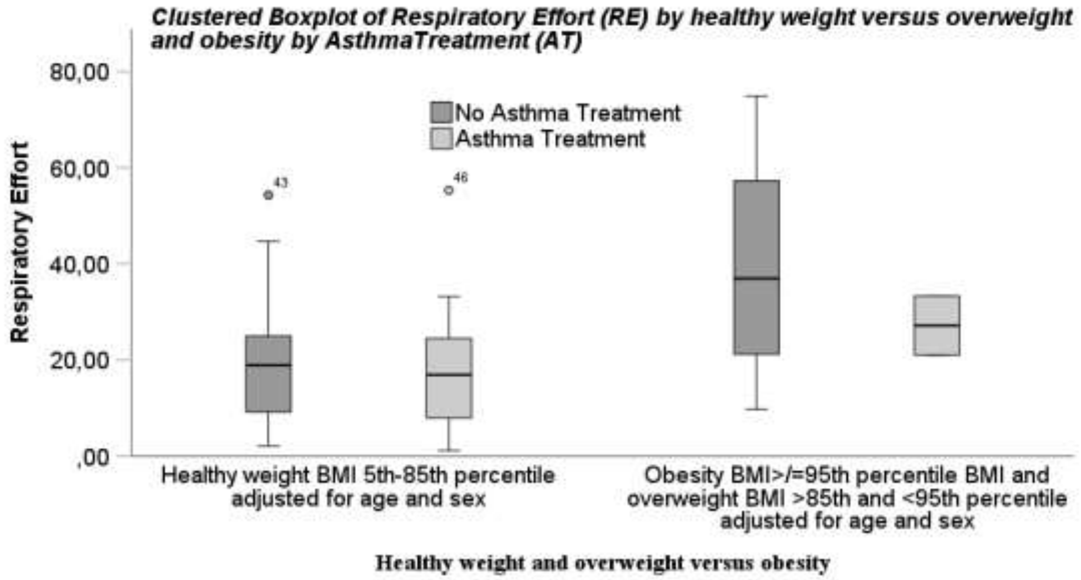

Note.This figure shows that when eviction diet is not taken into consideration, AT alone does not decrease the mean RE in healthy weight children. The obese/overweight who did not follow AT, they also had increased RE as compared to the obese/overweight patients who followed AT.

Suppl.Figure 4e.

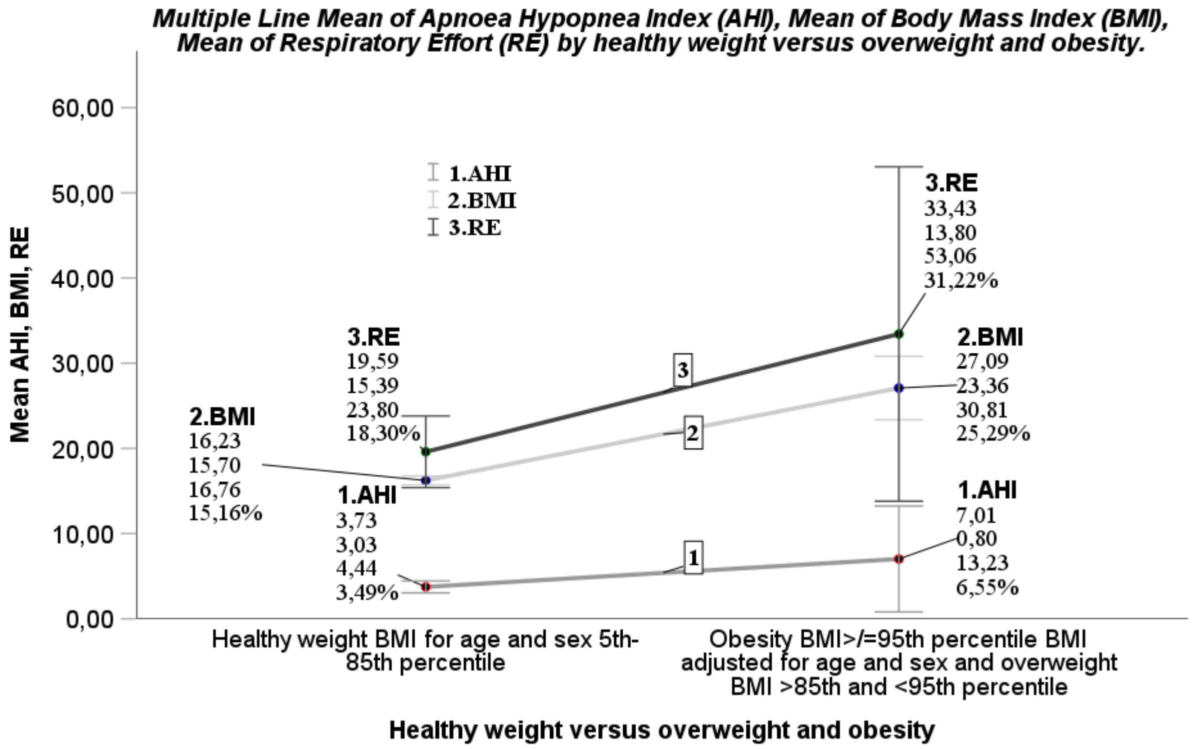

Note. This figure shows that the increase of the RE during sleep follows the increase of BMI more closely than the increase of AHI in the obese/overweight children as compared to healthy weight children. Mean values, low and high values of AHI, BMI and RE are shown. Percentages of healthy weight and overweight/obesity in each group are also shown.

Figure 4f.

Clustered Boxplot of Respiratory Effort (RE) by Asthma Treatment or Eviction Diet (ATED)by obesity and overweight

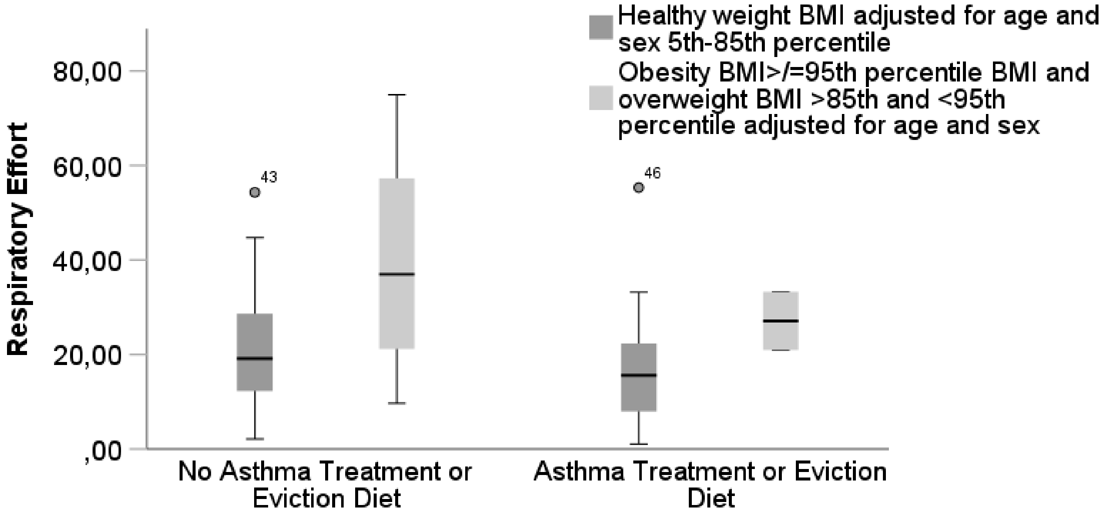

Note. This figure shows that the ATED keeps the level of the RE during sleep lower in both groups of healthy weight children and in obese/overweight children.

Figure 4g.

Clustered Boxplot of Respiratory Effort by RANIgE by obesity/overweight

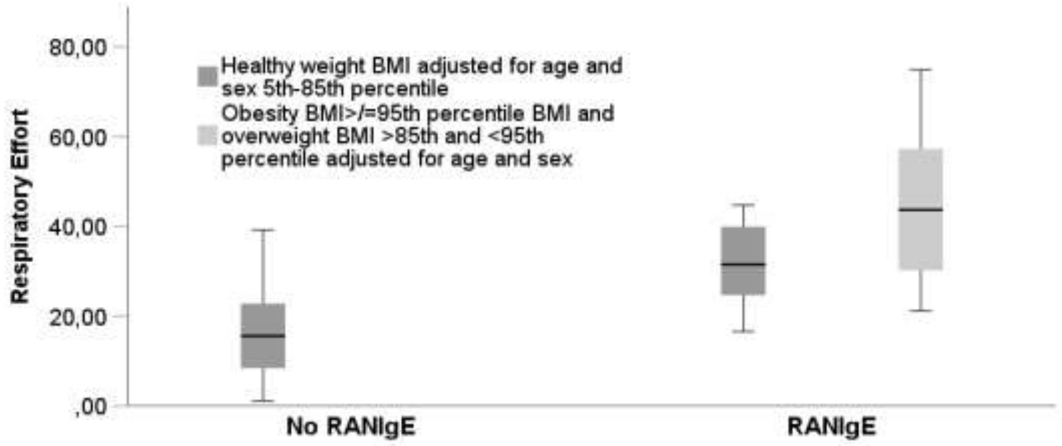

Note. This figure shows that the obese/overweight children suffer RANIgE. The obese who suffer RANIgE have more increased RE than the non-obese who suffer RANIgE. Moreover, the healthy weight patients who do not suffer RANIgE have lower RE than the healthy weight patients with RANIgE. Thus,the increased RE characterises the RANIgE and could favor obesity.

Suppl. Figure 5. ROC Curve of AHI to predict obesity and overweight group versus healthy weight

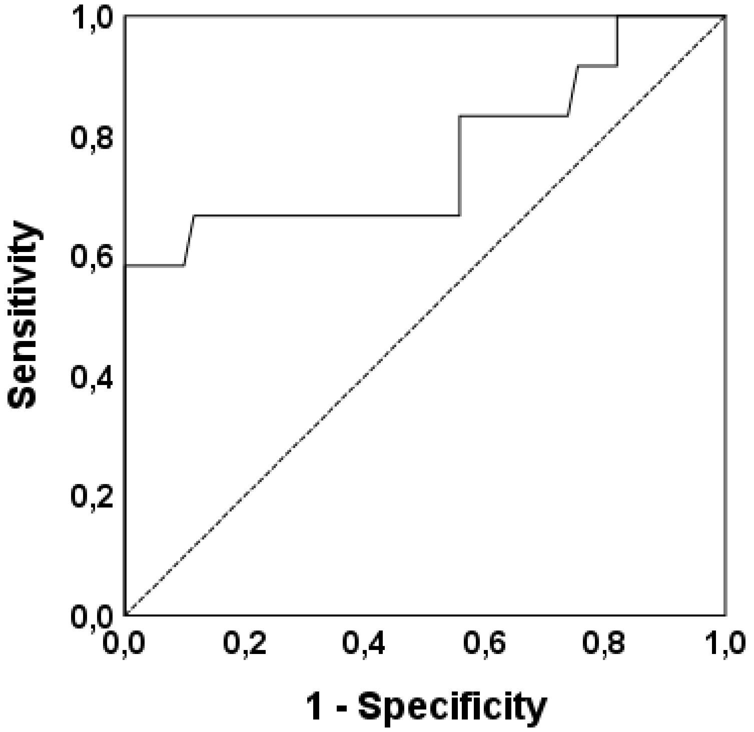

Suppl. Figure 6. ROC Curve of RANIgE to predict obesity and overweight versus healthy weight

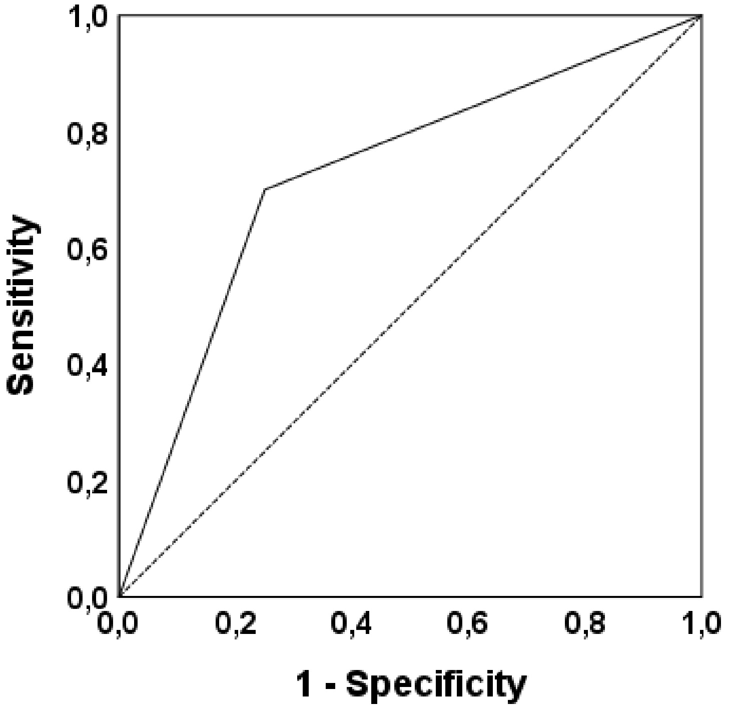

Suppl.Figure 7. ROC Curve of co-existence of Respiratory allergies and Non-IgE mediated allergies (RANIgE) to predict BMI

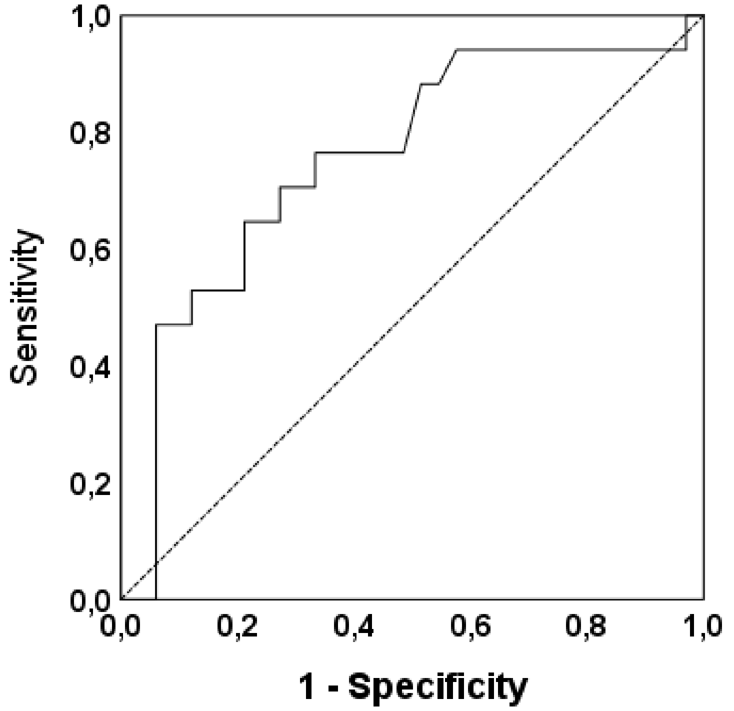

Suppl.Figure 8. ROC Curve of the co-existence of respiratory and Non-IgE mediated allergies (RANIgE) to predict Respiratory effort during sleep (RE)

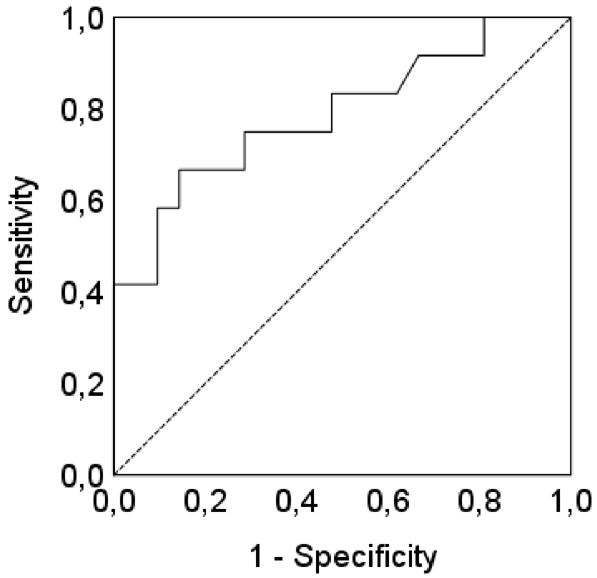

Suppl.Figure 9. ROC Curve of Asthma treatment or Eviction Diet (ATED) to predict obesity versus normal weight and overweight.

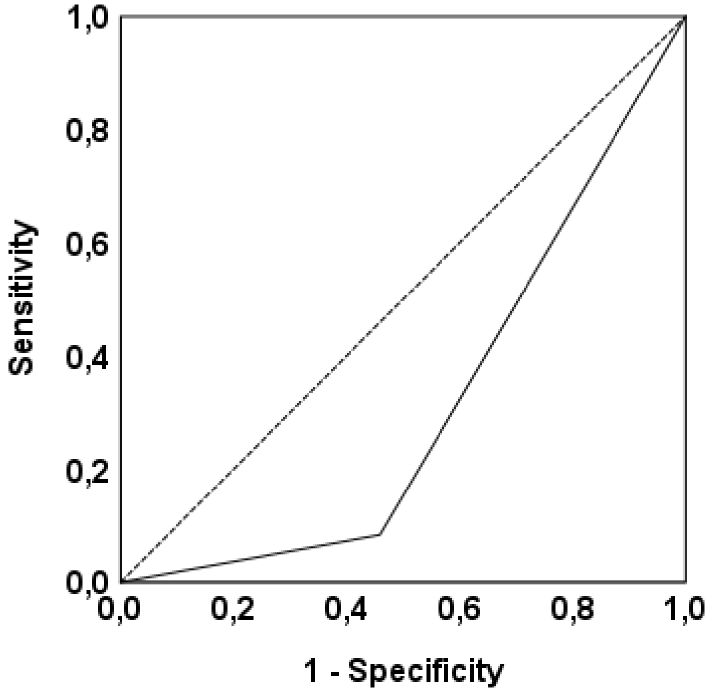

Suppl. Figure 10. Profile plot of the RE by both AT or ED and RANIgE**.**

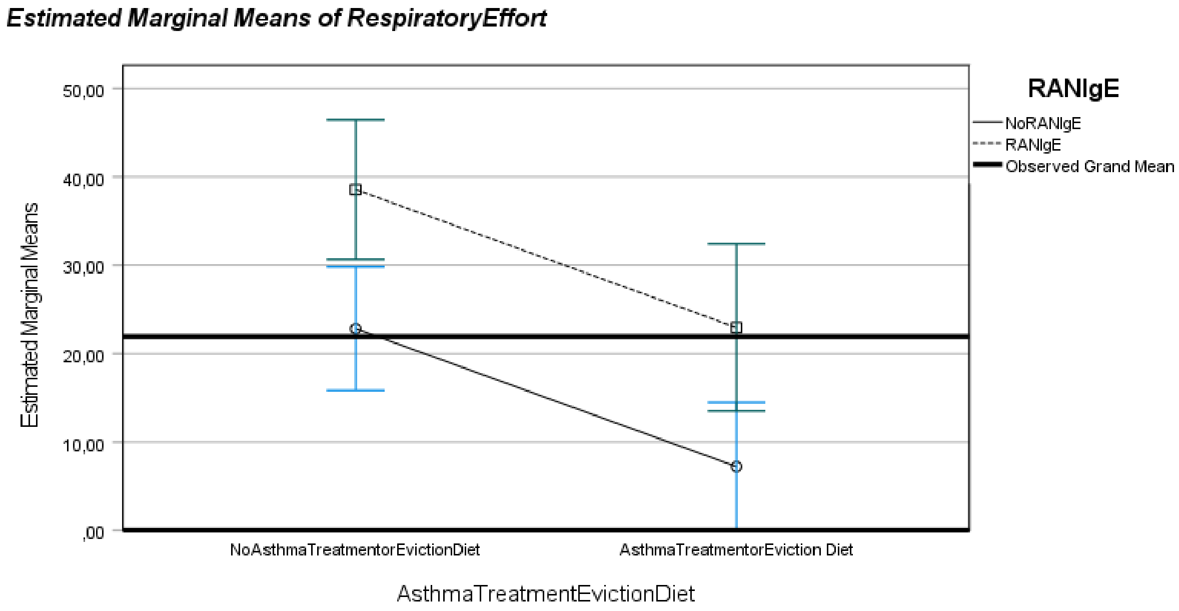

Suppl. Figure 11. Profile plot of RE according to RANIgE.ATED subgroups.

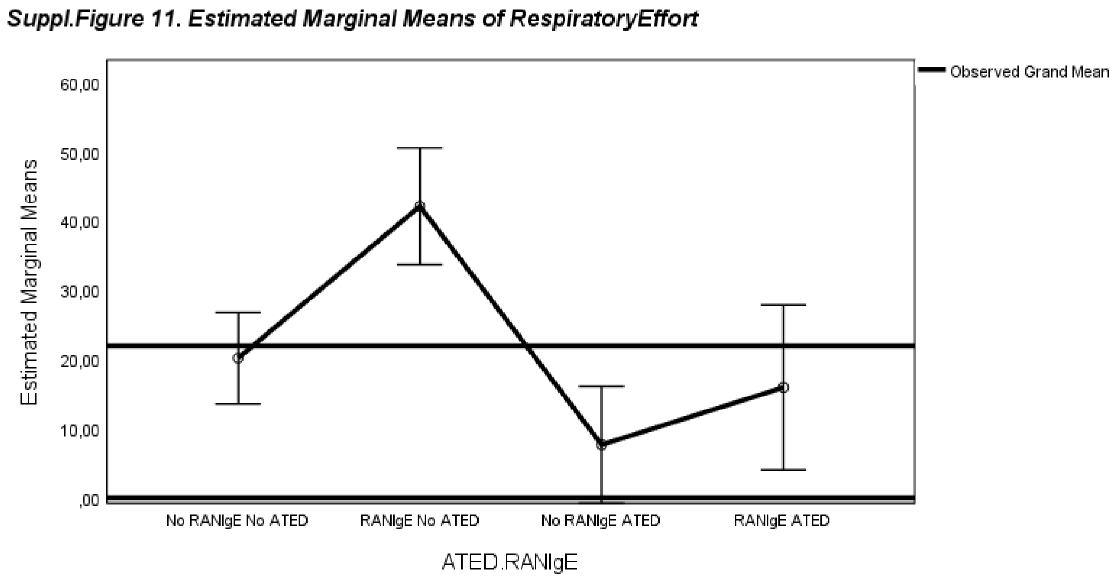

Suppl. Figure 12. Multiple Line Median of AHI, Median of BMI, Median of RespiratoryEffort by a combined (dummy) variable) ATED.RANIgE representing the treatment (asthma treatment or eviction diet) (AT or ED) and the co-existence of respiratory and non-IgE mediated allergies (RANIgE).

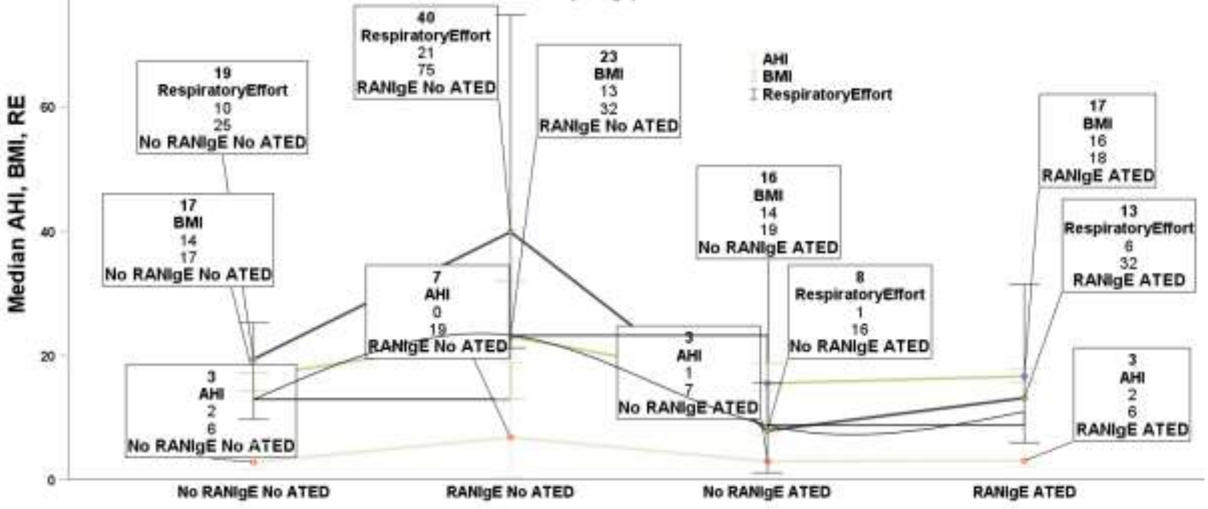

Note.This figure shows that the co-existence of RANIgE and lack of asthma treatment or eviction diet (AT or ED) results to a predominant increase of respiratory effort during sleep (RE) and to a mild increase on BMI and AHI. AT or ED and non-coexistence of RANIgE result to a prominent decrease of RE and a mild decrease of BMI and AHI. The decrease of BMI and AHI seems to be mainly result by the non-coexistence of RANIgE. The median values of AHI are similar in the No AT or ED No RANIgE and the no RANIgE ATED group.

Supplementary Figure 13. Profile plot of Respiratory Effort according to both RANIgE and healthy weight and overweight versus obesity.

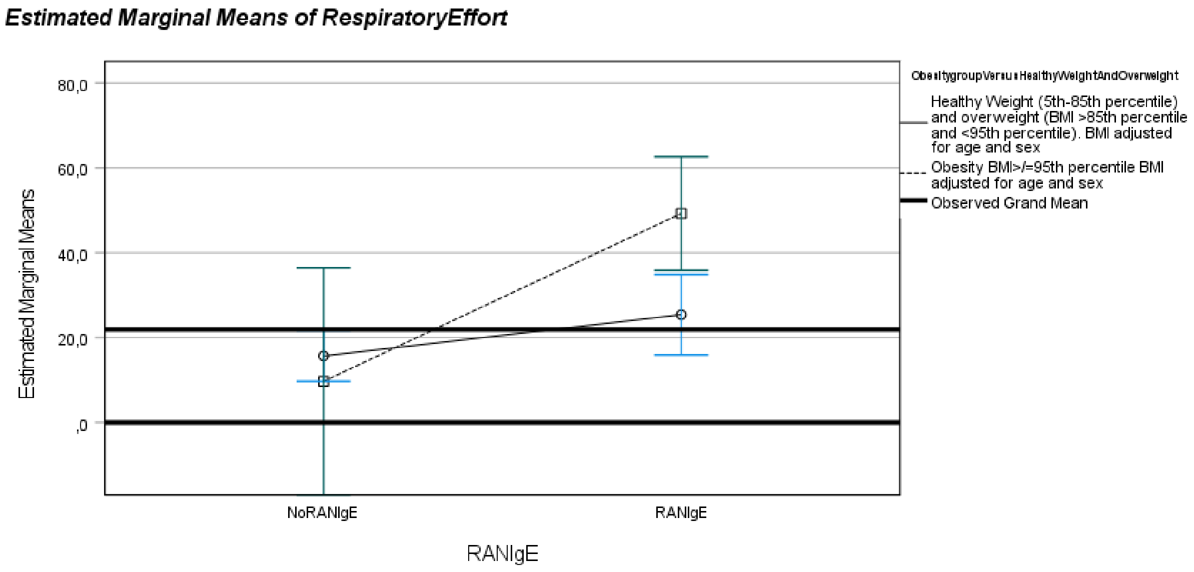

Supplementary Figure 14. Profile plot of AHI according to both AT or ED and healthy weight and overweight versus obesity.

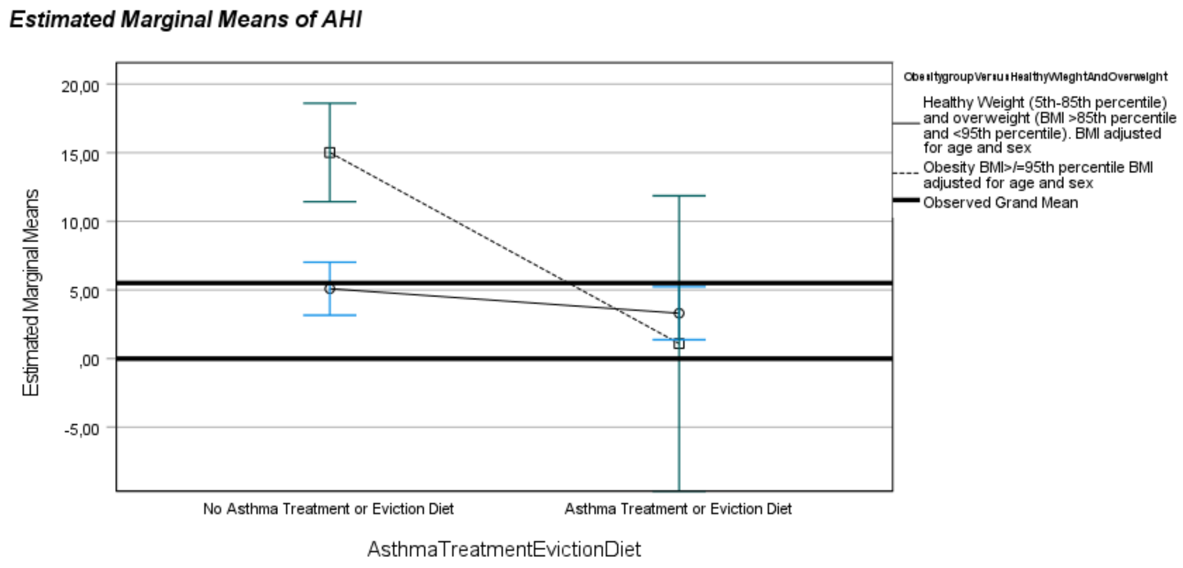

Supplementary Figure 15. Profile plot of AHI according to both AT or ED & obesity and overweight versus healthy weight.

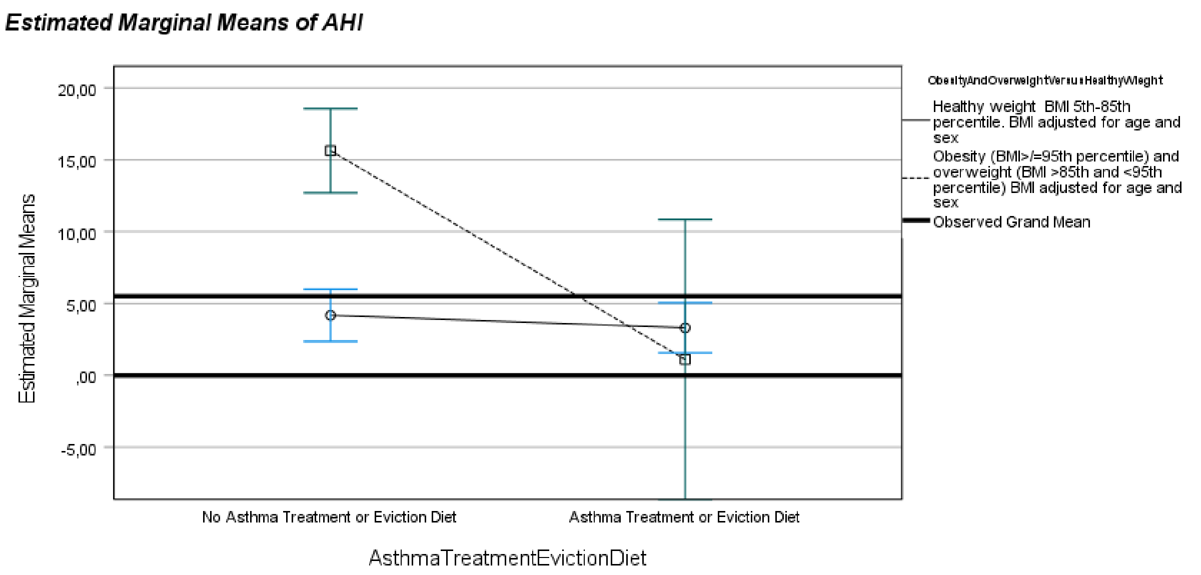

Suppl.Fig.16a. Estimated Means for Top Significant Fixed Effects: BMI by ATED

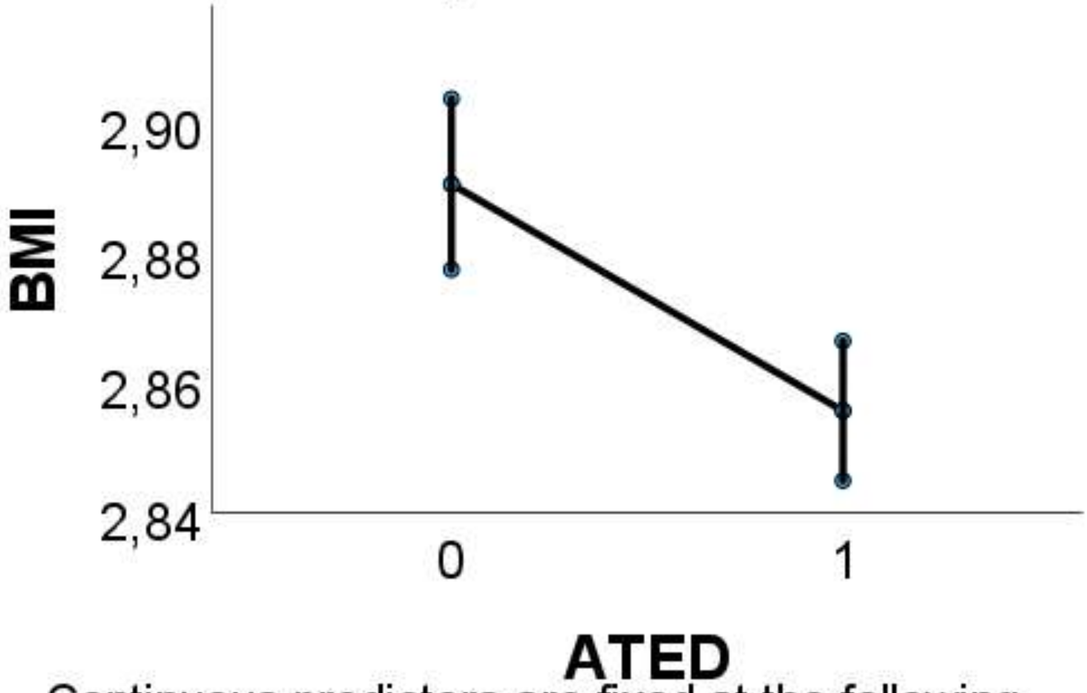

Continuous predictors are fixed at the following values: AHI = 4,4781, RE = 20,825

Suppl.Fig.16b. Estimated Means for Top Significant Fixed effects: BMI by RANIgE

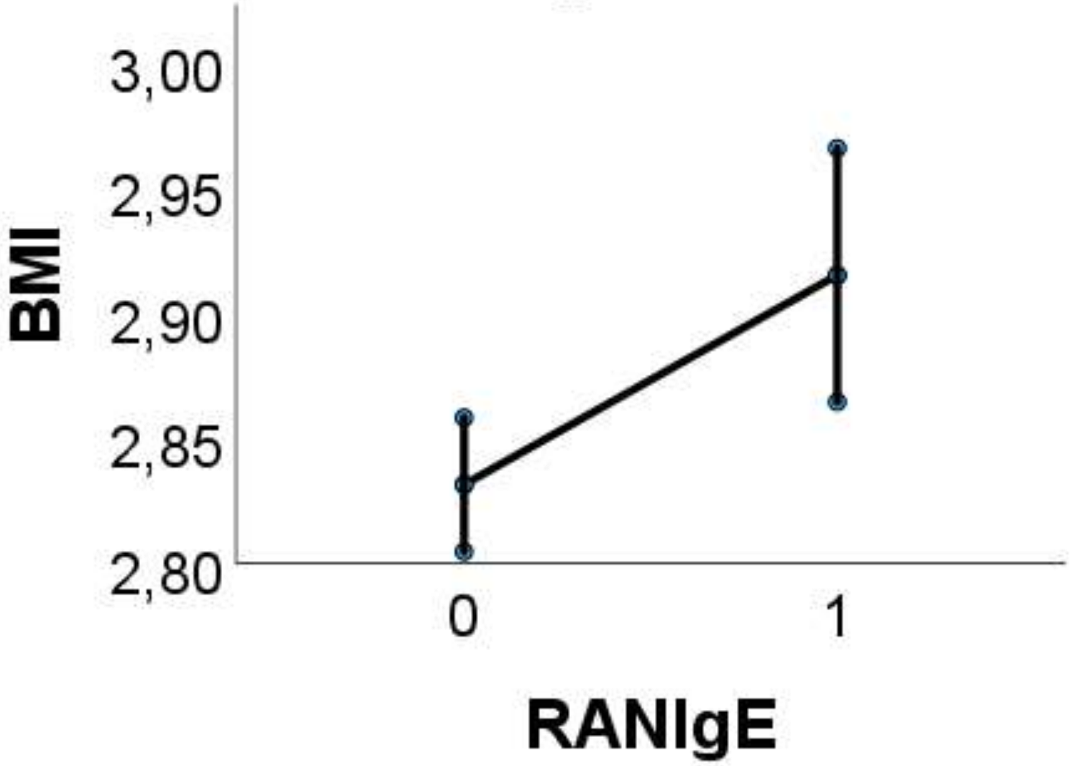

Continuous predictors are fixed at the following values: AHI = 4,4781, RE = 20,825

Suppl. Figure 17. Unstandardized Estimates of Path Analysis with serial mediation in AMOS.

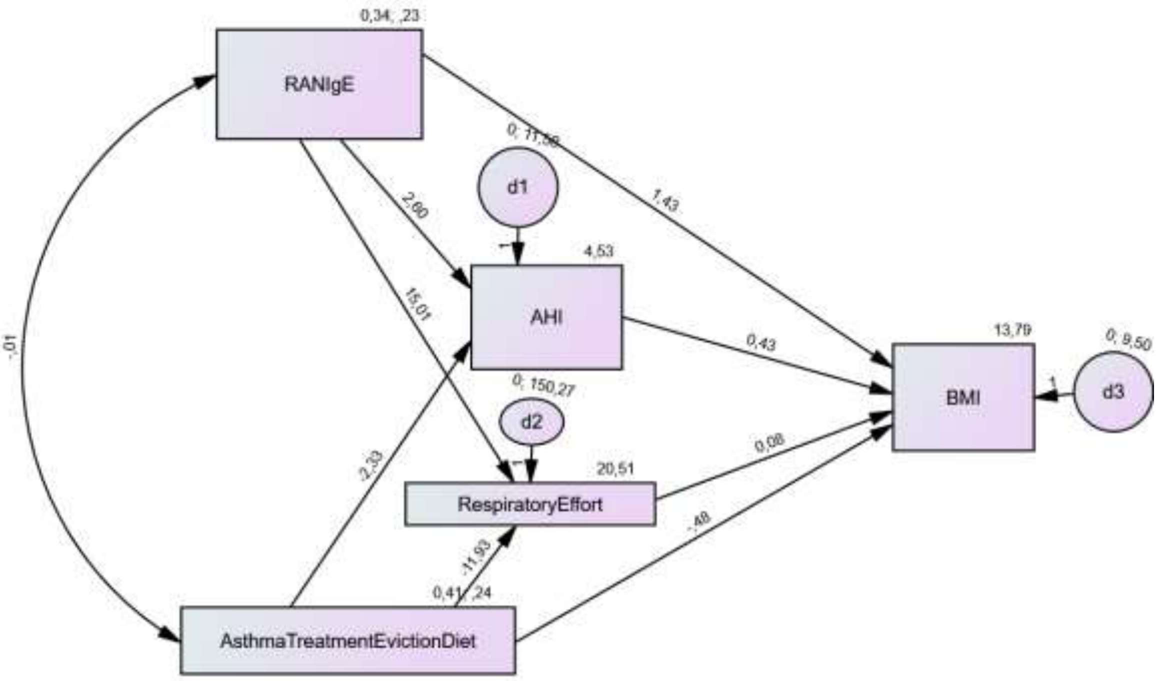

Suppl. Figure 18A-D (Case 10): A: SPT reaction to milk (L) and mites (D for Dermatophagoides pteronyssinus and A for Acarus) (Suppl. Figure 18A). The SPT in milk continued to increase (Suppl. Figure 18B), although the SPT to mites did not increase further (Suppl. Figure 18C). The patch tests were positive for milk and wheat (Suppl. Figure 18D).

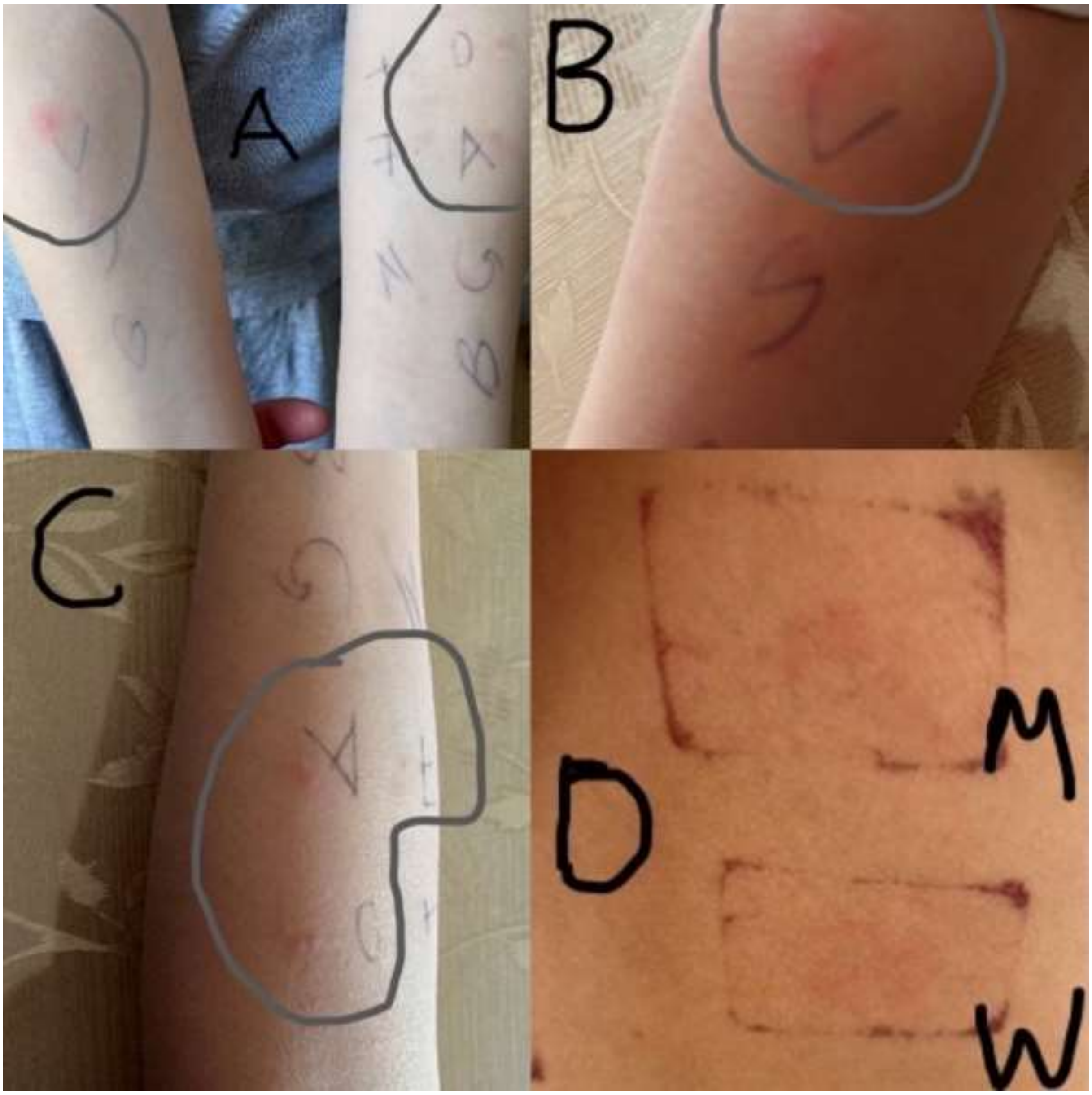

Supplement: Supplementary file 2 [file medi-105-e41730-s002.pdf]
