# Supplementary material for: Allergies, asthma treatment, and eviction diet have a significant impact on the respiratory effort during sleep and the apnea-hypopnea index in children with obstructive sleep apnea-obesity/asthma association: A STROBE-compliant study
Source: Medicine (Baltimore). 2026 Feb 13;105(7):e41730. doi: 10.1097/MD.0000000000041730 (PMC12908835; doi:10.1097/MD.0000000000041730)
Supplement: Supplementary file 3 [file medi-105-e41730-s003.pdf]

Suppl. Table 1. Statistical comparisons (ANOVA) were performed in between RE*AT, RE*AT or AE, AHI*Dust Mites A+NIgE, AHI*RANIgE, AHI*AT, AHI*AT or AE, RE*AT or AE, and RE* Dust Mites A+NIgE. *Eta, Eta-squared, Cohen's d, Hedge's correction, Glass's delta, were calculated to evaluate effect sizes.*

**Suppl. Table 1. Compare means ANOVA**	***Sig.***	***Eta***	***Eta-squared***	***Cohen's d***	***Hedge's correction***	***Glass's delta***
**RE*AT**	.099	.236	.056	-.519	-.511	.570
**RE*AT or AE**	.009	.366	.134	.788	.775	.918
**AHI*Dust Mites A+NIgE**	.043	.290	.084	.658	.648	.496
**AHI*RANIgE**	.055	.276	.076	.600	.591	.473
**AHI*AT**	.004	.272	.074	.580	.574	1.673
**AHI*AT or AE**	.002	.317	.107	.662	.655	1.904
**RE*RANIgE**	.002	.523	.274	1.237	1.207	.897
**RE* Dust Mites A+NIgE**	.022	.396	.157	.888	.886	.714

Suppl. Table 2. *Coordinates of the ROC Curve of* RANIgE to predict obesity (BMI>/=95th percentile) and overweight (BMI >85th percentile and <95th percentile) versus normal weight (BMI 5th-85th percentile). BMI adjusted for age and sex.

Test Result Variable(s): RANIgE

Positive if Greater Than or Equal To[a]	Sensitivity	1 – Specificity
-1,00	1,000	1,000
,50	,700	,250
2,00	,000	,000

The test result variable(s): RANIgE has at least one tie between the positive actual state group and the negative actual state group.

a. The smallest cutoff value is the minimum observed test value minus 1, and the largest cutoff value is the maximum observed test value plus 1. All the other cutoff values are the averages of two consecutive ordered observed test values.

Suppl. Table 3. *Coefficients* of a univariate general linear model which was estimated to investigate whether Asthma Treatment or Eviction Diet (AT or AE) and RANIgE predict RE [a]

		Collinearity Statistics	
Model		Tolerance	VIF
1	AT or ED	,981	1,020
	RANIgE	,981	1,020

a. Dependent Variable : RespiratoryEffort

b. Computed using alpha = ,05

Suppl. Table 4. *Tests of Between-Subjects Effects of* a univariate general linear model was estimated to investigate whether Asthma Treatment or Eviction Diet (AT or AE) and RANIgE predict RE.

Dependent Variable : RespiratoryEffort

Source	Type III Sum of Squares	Df	Mean Square	F	Sig.	Partial Eta Squared	Noncent. Parameter	Observed Power[b]
Corrected Model	4387,038[a]	2	2193,519	14,293	<,001	,488	28,586	,997
Intercept	15466,763	1	15466,763	100,781	<,001	,771	100,781	1,000
AT or ED	1924,297	1	1924,297	12,539	,001	,295	12,539	,929
RANIgE	1852,819	1	1852,819	12,073	,002	,287	12,073	,919
Error	4604,072	30	153,469					
Total	24862,070	33						
Corrected Total	8991,110	32						

a. R Squared = ,488 (Adjusted R Squared = ,454)

b. Computed using alpha = ,05

Suppl. Table 5. *Parameter Estimates of* a univariate general linear model was estimated to investigate whether Asthma Treatment or Eviction Diet (AT or AE) and RANIgE predict RE.

Dependent Variable : RespiratoryEffort

Parameter	B	Std. Error	T	Sig.	95% Confidence Interval Lower Bound	95% Confidence Interval Upper Bound	Partial Eta Squared	Noncent. Parameter	Observed Power[b]
Intercept	22,957	4,628	4,960	<,001	13,505	32,408	,451	4,960	,998
[AT or ED=0]	15,602	4,406	3,541	,001	6,604	24,601	,295	3,541	,929
[AT or ED=1]	0[a]	.	.	.	.	.	.	.	.
[RANIgE=0]	-15,729	4,527	-3,475	,002	-24,975	-6,484	,287	3,475	,919
[RANIgE=1]	0[a]	.	.	.	.	.	.	.	.

a. This parameter is set to zero because it is redundant.

Suppl. Table 6. *Coefficients*[a] of a univariate general linear model which was estimated to investigate whether RANIgE and obesity versus healthy weight/overweight (obesityVsHW/overw predict RE.

		Collinearity Statistics	
Model		Tolerance	VIF
1	RANIgE	,853	1,172
	ObesityVsHW/overw	,853	1,172

a. Dependent Variable : RespiratoryEffort

Suppl. Table 7. *Tests of Between-Subjects Effects* of a univariate general linear model which was estimated to investigate whether RANIgE and obesity versus healthy weight/overweight (obesityVsHW/overw predict RE.

Dependent Variable : RespiratoryEffort

Source	Type III Sum of Squares	Df	Mean Square	F	Sig.	Partial Eta Squared	Noncent. Parameter	Observed Power[b]
Corrected Model	4016,897[a]	3	1338,966	7,806	<,001	,447	23,419	,977
Intercept	7025,967	1	7025,967	40,962	<,001	,585	40,962	1,000
RANIgE	1704,915	1	1704,915	9,940	,004	,255	9,940	,861
ObesityVsHW/Overw	224,598	1	224,598	1,309	,262	,043	1,309	,198
RANIgE * ObesityVs HW/Overw	625,698	1	625,698	3,648	,066	,112	3,648	,455
Error	4974,213	29	171,525					
Total	24862,070	33						
Corrected Total	8991,110	32						

a. R Squared = ,447 (Adjusted R Squared = ,390)

b. Computed using alpha = ,05

Suppl. Table 8. *Parameter Estimates* of a univariate general linear model which was estimated to investigate whether RANIgE and obesity versus healthy weight/overweight (obesityVsHW/overw predict RE.

Dependent Variable : RespiratoryEffort

Parameter	B	Std. Error	t	Sig.	95% Confidence Interval Lower Bound	95% Confidence Interval Upper Bound	Partial Eta Squared	Noncent. Parameter	Observed Power[b]
Intercept	49,275	6,548	7,525	<,001	35,882	62,668	,661	7,525	1,000
[RANIgE=0]	-39,575	14,643	-2,703	,011	-69,522	-9,628	,201	2,703	,743
[RANIgE=1]	0[a]	.	.	.	.	.	.	.	.
[ObesityVsHW/Overw=,00]	-23,875	8,020	-2,977	,006	-40,278	-7,472	,234	2,977	,820
[Obesity Vs HW/Overw=1,00]	0[a]	.	.	.	.	.	.	.	.
[RANIgE=0] * [ObesityVsHW/Overw=,00]	29,860	15,634	1,910	,066	-2,115	61,835	,112	1,910	,455
[RANIgE=0] * [ObesityVs HW/Overw=1,00]	0[a]	.	.	.	.	.	.	.	.
[RANIgE=1] * [ObesityVs HW/Overw=,00]	0[a]	.	.	.	.	.	.	.	.
[RANIgE=1] * [ObesityVs HW/Overw=1,00]	0[a]	.	.	.	.	.	.	.	.

a. This parameter is set to zero because it is redundant.

b. Computed using alpha = ,05

Suppl. Table 9. *Coefficients*[a] *of a* univariate general linear model which was estimated to investigate whether AT or AE and obesity versus Healthy Weight and Overweight (obesityVsHW/Overw) predict AHI.

Model		Collinearity Statistics	
		Tolerance	VIF
1	AT or ED	,922	1,084
	Obesity Vs HW/Over	,922	1,084

a. Dependent Variable : AHI

Suppl. Table 10*. Between-Subjects Factors of a univariate general linear model which was estimated to investigate whether AT or AE and obesity versus Healthy Weight and Overweight (obesityVsHW/Overw) predict AHI.*

		Value Label	N
AT or ED	0	No AT or ED	40
	1	AT or ED	32
ObesityVsHW/Overw	,00	Healthy Weight (5th-85th percentile) and overweight (BMI >85th percentile and <95th percentile). BMI adjusted for age and sex	62
	1,00	Obesity BMI>/=95th percentile BMI adjusted for age and sex	10

Suppl. Table 11. *Tests of Between-Subjects Effects of a* a univariate general linear model which was estimated to investigate whether AT or AE and obesity versus Healthy Weight and Overweight (obesityVsHW/Overw) predict AHI.

Dependent Variable : AHI

Source	Type III Sum of Squares	df	Mean Square	F	Sig.	Partial Eta Squared	Noncent. Parameter	Observed Power[b]
Corrected Model	989,690[a]	3	329,897	11,336	<,001	,333	34,009	,999
Intercept	511,505	1	511,505	17,577	<,001	,205	17,577	,985
AT or ED	209,916	1	209,916	7,213	,009	,096	7,213	,754
ObesityVsHW/Overw	50,724	1	50,724	1,743	,191	,025	1,743	,256
AT or ED * ObesityVsHW/Overw	125,262	1	125,262	4,304	,042	,060	4,304	,534
Error	1978,853	68	29,101					
Total	5154,250	72						
Corrected Total	2968,543	71						

a. R Squared = ,333 (Adjusted R Squared = ,304)

b. Computed using alpha = ,05

Suppl. Table 12. *Parameter Estimates of a* a univariate general linear model which was estimated to investigate whether AT or AE and obesity versus Healthy Weight and Overweight (obesityVsHW/Overw) predict AHI.

Dependent Variable : AHI

					95% Confidence Interval				
Parameter	B	Std. Error	T	Sig.	Lower Bound	Upper Bound	Partial Eta Squared	Noncent. Parameter	Observed Power[b]
Intercept	1,100	5,395	,204	,839	-9,665	11,865	,001	,204	,055
[AT or ED =0]	13,922	5,686	2,448	,017	2,575	25,269	,081	2,448	,675
[AT or ED =1]	0[a]	.	.	.	.	.	.	.	.
[ObesityVsHW/Overw =,00]	2,206	5,481	,403	,689	-8,730	13,143	,002	,403	,068
[ObesityVsHW/Overw =1,00]	0[a]	.	.	.	.	.	.	.	.
[AT or ED =0] * [ObesityVsHW/Overw =,00]	-12,135	5,849	-2,075	,042	-23,807	-,463	,060	2,075	,534
[AT or ED =0] * [ObesityVsHW/Overw =1,00]	0[a]	.	.	.	.	.	.	.	.
[AT or ED =1] * [ObesityVsHW/Overw =,00]	0[a]	.	.	.	.	.	.	.	.
[AT or ED =1] * ObesityVsHW/Overw [=1,00]	0[a]	.	.	.	.	.	.	.	.

a. This parameter is set to zero because it is redundant.

b. Computed using alpha = ,05

Suppl. Table 13. Coefficients[a] of a univariate general linear model was estimated to investigate whether AT or AE and obesity predict AHI.

Model
AT or ED
obesity/overwVsHW

a. Dependent Variable : AHI

Suppl. Table 14. *Tests of Between-Subjects* Effects of a univariate general linear model which was estimated to investigate whether AT or AE and obesity predict AHI.

Dependent Variable : AHI

Source	Type III Sum of Squares	df	Mean Square	F	Sig.	Partial Eta Squared	Noncent. Parameter	Observed Power[b]
Corrected Model	1349,566[a]	3	449,855	18,895	<,001	,455	56,684	1,000
Intercept	506,668	1	506,668	21,281	<,001	,238	21,281	,995
AT or ED	205,017	1	205,017	8,611	,005	,112	8,611	,825
obesity/overwVsHW	73,975	1	73,975	3,107	,082	,044	3,107	,412
AT or ED * *obesity/overwVsHW*	161,349	1	161,349	6,777	,011	,091	6,777	,728
Error	1618,977	68	23,808					
Total	5154,250	72						
Corrected Total	2968,543	71						

a. R Squared = ,455 (Adjusted R Squared = ,431)

b. Computed using alpha = ,05

Suppl. Table 15. *Parameter Estimates of a univariate general linear model which was estimated to investigate whether AT or AE and obesity predict AHI.* Dependent Variable : AHI

Parameter	B	Std. Error	t	Sig.	95% Confidence Interval Lower Bound	95% Confidence Interval Upper Bound	Partial Eta Squared	Noncent. Parameter	Observed Power[b]
Intercept	1,100	4,879	,225	,822	-8,637	10,837	,001	,225	,056
[AT or ED =0]	14,536	5,096	2,852	,006	4,367	24,706	,107	2,852	,803
[AT or ED =1]	0[a]	.	.	.	.	.	.	.	.
[*obesity/overwVsHW* =,00]	2,206	4,957	,445	,658	-7,686	12,099	,003	,445	,072
[*obesity/overwVsHW* =1,00]	0[a]	.	.	.	.	.	.	.	.
[AT or ED =0] * [*obesity/overwVsHW* =,00]	-13,667	5,250	-2,603	,011	-24,143	-3,191	,091	2,603	,728
[AT or ED =0] * [*obesity/overwVsHW* =1,00]	0[a]	.	.	.	.	.	.	.	.
[AT or ED =1] * [*obesity/overwVsHW* =,00]	0[a]	.	.	.	.	.	.	.	.
[AT or ED =1] * [*obesity/overwVsHW* =1,00]	0[a]	.	.	.	.	.	.	.	.

a. This parameter is set to zero because it is redundant.

b. Computed using alpha = ,05

Suppl. Table 16. *Tests of Between-Subjects Effects* of a univariate general linear model which was estimated to investigate whether a variable with combined information for AT or AE and RANIgE predict RE.

Dependent Variable : RespiratoryEffort

Source	Type III Sum of Squares	Df	Mean Square	F	Sig.	Partial Eta Squared	Noncent. Parameter	Observed Power[b]
Corrected Model	5059,958[a]	3	1686,653	12,442	<,001	,563	37,327	,999
Intercept	12767,327	1	12767,327	94,184	<,001	,765	94,184	1,000
ATED.RANIgE	5059,958	3	1686,653	12,442	<,001	,563	37,327	,999
Error	3931,152	29	135,557					
Total	24862,070	33						
Corrected Total	8991,110	32						

a. R Squared = ,563 (Adjusted R Squared = ,518)

b. Computed using alpha = ,05

Suppl.Table 17. *Parameter* Estimates of a univariate general linear model which was estimated to investigate whether a variable with combined information for AT or AE and RANIgE predict RE.

Dependent Variable : RespiratoryEffort

Parameter	B	Std. Error	t	Sig.	95% Confidence Interval Lower Bound	95% Confidence Interval Upper Bound	Partial Eta Squared	Noncent. Parameter	Observed Power[b]
Intercept	15,925	5,821	2,736	,011	4,019	27,831	,205	2,736	,753
[ATED.RANIgE=,00]	4,237	6,657	,636	,530	-9,379	17,852	,014	,636	,094
[ATED.RANIgE=1,00]	26,150	7,130	3,668	<,001	11,568	40,732	,317	3,668	,943
[ATED.RANIgE=2,00]	-8,263	7,130	-1,159	,256	-22,845	6,320	,044	1,159	,202
[ATED.RANIgE=3,00]	0[a]	.	.	.	.	.	.	.	.

a. This parameter is set to zero because it is redundant.

b. Computed using alpha = ,05

Suppl. Table 18. *Multiple Comparisons of* a univariate general linear model which was estimated to investigate whether a variable with combined information for AT or AE and RANIgE predict RE. Post hoc comparisons (LSD) have shown that the group RANIgENoATAE had the highest levels of RE, while the group NoRANIgE.ATAE had the lowest levels of RE.

Dependent Variable : RespiratoryEffort

	(I) ATED.RANIgE	(J) ATED.RANIgE	Mean Difference (I-J)	Std. Error	Sig.	95% Confidence Interval Lower Bound	95% Confidence Interval Upper Bound
LSD	No RANIgE No ATED	RANIgE No ATED	-21,9135*	5,23183	<,001	-32,6138	-11,2132
		No RANIgE ATED	12,4990*	5,23183	,024	1,7987	23,1993
		RANIgE ATED	4,2365	6,65708	,530	-9,3787	17,8518
	RANIgE No ATED	No RANIgE No ATED	21,9135*	5,23183	<,001	11,2132	32,6138
		No RANIgE ATED	34,4125*	5,82145	<,001	22,5063	46,3187
		RANIgE ATED	26,1500*	7,12979	<,001	11,5679	40,7321
	No RANIgE ATED	No RANIgE No ATED	-12,4990*	5,23183	,024	-23,1993	-1,7987
		RANIgE No ATED	-34,4125*	5,82145	<,001	-46,3187	-22,5063
		RANIgE ATED	-8,2625	7,12979	,256	-22,8446	6,3196
	RANIgE ATED	No RANIgE No ATED	-4,2365	6,65708	,530	-17,8518	9,3787
		RANIgE No ATED	-26,1500*	7,12979	<,001	-40,7321	-11,5679
		No RANIgE ATED	8,2625	7,12979	,256	-6,3196	22,8446

Based on observed means.

The error term is Mean Square (Error) = 135,557.

*. The mean difference is significant at the ,05 level.

Suppl. Table 19. *Omnibus Test[a] of a poisson regression analysis was used to investigate whether AT or ED and RE could predict BMI.*

Likelihood Ratio Chi-Square	Df	Sig.
5,982	2	,050

Dependent Variable : BMI

Model : (Intercept), RespiratoryEffort, AsthmaTreatmentEvictionDiet

a. Compares the fitted model against the intercept-only model.

Suppl. Table 20. *Goodness of Fit*[a] *of a poisson regression analysis which was used investigate whether AT or ED and RE c*ould predict BMI.

	Value	Df	Value/df
Deviance	8,658	8	1,082
Scaled Deviance	8,658	8	
Pearson Chi-Square	8,378	8	1,047
Scaled Pearson Chi-Square	8,378	8	
Log Likelihood[b]	-30,063		
Akaike's Information Criterion (AIC)	66,127		
Finite Sample Corrected AIC (AICC)	69,555		
Bayesian Information Criterion (BIC)	67,321		
Consistent AIC (CAIC)	70,321		

Dependent Variable : BMI

Model : (Intercept), RespiratoryEffort, AsthmaTreatmentEvictionDiet

a. Information criteria are in smaller-is-better form.
b. The full log likelihood function is displayed and used in computing information criteria.

Suppl. Table 21. *Parameter* Estimates of a poisson regression analysis which was used to *investigate whether AT or ED and RE could predict BMI.*

Parameter	B	Std. Error	95% Wald Confidence Interval Lower	95% Wald Confidence Interval Upper	Hypothesis Test Wald Chi-Square	Hypothesis Test Df	Hypothesis Test Sig.
(Intercept)	2,662	,1834	2,303	3,022	210,671	1	,000
RespiratoryEffort	,009	,0035	,002	,016	6,092	1	,014
[AT or ED =0]	-,074	,2025	-,471	,323	,133	1	,715
[AT or ED =1]	0[a]	.	.	.	.	.	.
(Scale)	1[b]						

Dependent Variable : BMI

Model : (Intercept), RespiratoryEffort, AsthmaTreatmentEvictionDiet

a. Set to zero because this parameter is redundant.

b. Fixed at the displayed value.

Suppl. Table 22. *Coefficients[a] of a binary logistic model of regression through generalized linear model was performed to ascertain the effects of RE and ATAE on the likelihood that participants develop obesity or overweight versus healthy weight (obesity/overwVsHW).*

Model		Collinearity Statistics	
		Tolerance	VIF
1	AT or ED	,866	1,155
	RespiratoryEffort	,866	1,155

a. Dependent Variable: Obesity and Overweight Versus Healthy Weight (obesity/overwVsHW)

Suppl. Table 23. *Omnibus Test[a] of a binary logistic model of regression through generalized linear model was performed to ascertain the effects of RE and ATAE on the likelihood that participants develop obesity or overweight versus healthy weight (obesity/overwVsHW).*

Likelihood Ratio Chi-Square	Df	Sig.
8,000	2	,018

Dependent Variable: Obesity and Overweight Versus Healthy Weight

Model: (Intercept), Respiratory Effort, Asthma Treatment Eviction Diet

a. Compares the fitted model against the intercept-only model.

Suppl. Table 24. *Parameter Estimates of a binary logistic model of regression through generalized linear model which was performed to ascertain the effects of RE and ATAE on the likelihood that participants develop obesity or overweight versus healthy weight (obesity/overwVsHW).*

			95% Wald Confidence Interval		Hypothesis Test		
Parameter	B	Std. Error	Lower	Upper	Wald Chi-Square	Df	Sig.
(Intercept)	4,002	1,2468	1,558	6,445	10,301	1	,001
RespiratoryEffort	-,053	,0261	-,104	-,002	4,120	1	,042
[AT or ED =0]	-1,225	1,1661	-3,510	1,061	1,103	1	,294
[AT or ED =1]	0[a]	.	.	.	.	.	.
(Scale)	1[b]						

Dependent Variable: ObesityAndOverweightVersusHealthyWeight
Model: (Intercept), RespiratoryEffort, AT or ED
a. Set to zero because this parameter is redundant.
b. Fixed at the displayed value.

Suppl. Table 25. *Goodness of Fit[a] of a binary logistic model of regression through generalized linear model which was performed to ascertain the effects of RE and ATAE on the likelihood that participants develop obesity or overweight versus healthy weight (obesity/overwVsHW).*

	Value	Df	Value/df
Deviance	35,967	47	,765
Scaled Deviance	35,967	47	
Pearson Chi-Square	48,865	47	1,040
Scaled Pearson Chi-Square	48,865	47	
Log Likelihood[b]	-17,983		
Akaike's Information Criterion (AIC)	41,967		
Finite Sample Corrected AIC (AICC)	42,488		
Bayesian Information Criterion (BIC)	47,703		
Consistent AIC (CAIC)	50,703		

Dependent Variable: ObesityAndOverweightVersusHealthyWeight

Model: (Intercept), RespiratoryEffort, AT or ED

a. Information criteria are in smaller-is-better form.

b. The full log likelihood function is displayed and used in computing information criteria.

Univariate general linear model UGLM are reported in the Suppl. Tables 3-18 and 26-32. Some UGLM are reported in more details in the Repository Text

Suppl. Table 26. *Coefficients[a] of a univariate general linear model which was estimated to investigate whether AT or AE and RANIgE predict BMI while controlling for AHI as a covariate.*

		Collinearity Statistics	
Model		Tolerance	VIF
1	RANIgE	,916	1,091
	AT or ED	,837	1,195
	AHI	,802	1,247

a. Dependent Variable : BMI

Suppl. Table 27. *Tests of Between-Subjects Effects of a univariate general linear model which was estimated to investigate whether AT or AE and RANIgE predict BMI while controlling for AHI as a covariate when controlling for the assumption of homogeneity of regression slopes.*

Dependent Variable : BMI

Source	Type III Sum of Squares	Df	Mean Square	F	Sig.	Partial Eta Squared	Noncent. Parameter	Observed Power[b]
Corrected Model	4628,111[a]	6	771,352	38,667	<,001	,847	232,000	1,000
Intercept	3403,608	1	3403,608	170,617	<,001	,802	170,617	1,000
RANIgE	162,755	1	162,755	8,159	,007	,163	8,159	,797
ATorED	130,600	1	130,600	6,547	,014	,135	6,547	,705
AHI	40,280	1	40,280	2,019	,163	,046	2,019	,284
RANIgE * ATorED * AHI	1466,611	3	488,870	24,506	<,001	,636	73,519	1,000
Error	837,849	42	19,949					
Total	24137,230	49						
Corrected Total	5465,960	48						

a. R Squared = ,847 (Adjusted R Squared = ,825)

b. Computed using alpha = ,05

Abbreviations : AsthmaTreatmentEvictionDiet : ATorED

Suppl. Table 28. *Parameter Estimates of a univariate general linear model which was estimated to investigate whether AT or AE and RANIgE predict BMI while controlling for AHI as a covariate when controlling for the assumption of homogeneity of regression slopes.*

Dependent Variable : BMI

					95% Confidence Interval				
Parameter	B	Std. Error	T	Sig.	Lower Bound	Upper Bound	Partial Eta Squared	Noncent. Parameter	Observed Power[b]
Intercept	21,977	2,562	8,579	<,001	16,807	27,146	,637	8,579	1,000
[RANIgE=0]	-6,745	2,361	-2,856	,007	-11,510	-1,979	,163	2,856	,797
[RANIgE=1]	0[a]	.	.	.	.	.	.	.	.
[ATD=0]	-5,412	2,115	-2,559	,014	-9,681	-1,143	,135	2,559	,705
[ATorED =1]	0[a]	.	.	.	.	.	.	.	.
AHI	-1,255	,808	-1,553	,128	-2,885	,376	,054	1,553	,329
[RANIgE=0] * [ATorED =0] * AHI	3,386	,849	3,991	<,001	1,674	5,099	,275	3,991	,974
[RANIgE=0] * [ATorED =1] * AHI	1,357	,808	1,680	,100	-,273	2,987	,063	1,680	,375
[RANIgE=1] * [ATorED =0] * AHI	1,797	,767	2,343	,024	,249	3,345	,116	2,343	,629
[RANIgE=1] * [ATorED =1] * AHI	0[a]	.	.	.	.	.	.	.	.

a. This parameter is set to zero because it is redundant.

b. Computed using alpha = ,05

Abbreviations: AsthmaTreatmentEvictionDiet : ATorED

Suppl Table 29. *Levene's Test of Equality of Error Variances*[a]

Dependent Variable: BMI

F	df1	df2	Sig.
2,866	3	20	,062

Tests the null hypothesis that the error variance of the dependent variable is equal across groups.

a. Design: Intercept + AsthmaTreatmentOrEvictionDiet * RANIgE * AHI + RANIgE * RespiratoryEffort

Suppl Table 30. *F Test for Heteroskedasticity*[a,b,c]

F	df1	df2	Sig.
1,463	6	17	,249

a. Dependent variable: BMI

b. Tests the null hypothesis that the variance of the errors does not depend on the values of the independent variables.

c. Design: Intercept + AsthmaTreatmentOrEvictionDiet * RANIgE * AHI + RANIgE * RespiratoryEffort

Suppl Table 31. *Tests of Between-Subjects Effects* of a univariate general linear model which was estimated to investigate whether AT or ED and RANIgE predict BMI while controlling AHI and RE as covariates.

Dependent Variable : BMI

Source	Type III Sum of Squares	df	Mean Square	F	Sig.	Partial Eta Squared	Noncent. Parameter	Observed Power[b]
Corrected Model	361,659[a]	6	60,277	6,336	,001	,691	38,016	,985
Intercept	602,418	1	602,418	63,324	<,001	,788	63,324	1,000
AsthmaTreatmentOr EvictionDiet * RANIgE * AHI	157,748	4	39,437	4,145	,016	,494	16,582	,823
RANIgE * RespiratoryEffort	20,315	2	10,157	1,068	,366	,112	2,135	,206
Error	161,726	17	9,513					
Total	8277,800	24						
Corrected Total	523,385	23						

a. R Squared = ,691 (Adjusted R Squared = ,582)

b. Computed using alpha = ,05

Suppl Table 32. *Parameter Estimates* of a univariate general linear model was estimated to investigate whether AT or ED and RANIgE predict BMI while controlling AHI and RE as covariates.

Dependent Variable : BMI

Parameter	B	Std. Error	t	Sig.	95% Confidence Interval Lower Bound	95% Confidence Interval Upper Bound	Partial Eta Squared	Noncent. Parameter	Observed Power[a]
Intercept	14,482	1,820	7,958	<,001	10,642	18,322	,788	7,958	1,000
[AsthmaTreatmentOrEvictionDiet=0] * [RANIgE=0] * AHI	-,190	,344	-,553	,587	-,915	,535	,018	,553	,082
[AsthmaTreatmentOrEvictionDiet=0] * [RANIgE=1] * AHI	,708	,185	3,817	,001	,317	1,099	,462	3,817	,949
[AsthmaTreatmentOrEvictionDiet=1] * [RANIgE=0] * AHI	,312	,609	,512	,615	-,974	1,598	,015	,512	,077
[AsthmaTreatmentOrEvictionDiet=1] * [RANIgE=1] * AHI	,064	,553	,115	,910	-1,102	1,229	,001	,115	,051
[RANIgE=0] * RespiratoryEffort	,096	,080	1,212	,242	-,071	,264	,080	1,212	,208
[RANIgE=1] * RespiratoryEffort	,064	,052	1,222	,238	-,046	,174	,081	1,222	,211

a. Computed using alpha = ,05

A Generalized Linear Mixed Model(GLMM) to evaluate the coefficients estimates of AHI, RE, ATED and RANIgE on BMI as a target variable (Suppl.Tables 33-44).

Suppl.Table 33. *Model Summary of a Generalized Linear Mixed Model to evaluate the coefficients estimates of AHI, RE, ATED and RANIgE on BMI as a target variable.*

Target		BMI
Probability Distribution		Gamma
Link Function		Log
Information Criterion	Akaike Corrected	7,416
	Bayesian	9,507

Information criteria are based on the -2 log likelihood (2,916) and are used to compare models. Models with smaller information criterion values fit better.

Suppl.Table 34. *Coefficients of Determination[a] of of a Generalized Linear Mixed Model to evaluate the coefficients estimates of AHI, RE, ATED and RANIgE on BMI as a target variable.*

Pseudo-R Square Measures	Marginal	,286
	Conditional	,536

a. Observation-level variance estimated via trigamma method.

Suppl. Table 35. *Intraclass Correlation Coefficients[a] of a of a Generalized Linear Mixed Model to evaluate the coefficients estimates of AHI, RE, ATED and RANIgE on BMI as a target variable.*

Overall	Adjusted	,351
ICCs	Conditional	,250

a. Observation-level variance estimated via trigamma method.

Suppl.Table 36. *Fixed Effects[a] of of a Generalized Linear Mixed Model to evaluate the coefficients estimates of AHI, RE, ATED and RANIgE on BMI as a target variable.*

Source	F	df1	df2	Sig.
Corrected Model	29,686	1	27	<,001
AHI	29,686	1	27	<,001
RE	24,918	1	27	<,001
RANIgE	4,924	1	27	,035
ATED	882,289	1	27	<,001

Probability distribution: Gamma

Link function: Log

a. Target: BMI

Suppl. Table 37. *Fixed Coefficients[a] of a Generalized Linear Mixed Model to evaluate the coefficients estimates of AHI, RE, ATED and RANIgE on BMI as a target variable.*

					95% Confidence Interval	
Model Term	Coefficient	Std. Error	T	Sig.	Lower	Upper
Intercept	2,745	,0239	114,645	<,001	2,696	2,794
AHI	,018	,0033	5,448	<,001	,011	,025
RE	,003	,0007	4,992	<,001	,002	,005
RANIgE=0	-,083	,0376	-2,219	,035	-,161	-,006
RANIgE=1	0[b]	.	.	.	.	.
ATED=0	,035	,0012	29,703	<,001	,032	,037
ATED=1	0[b]	.	.	.	.	.

Probability distribution: Gamma

Link function: Log

a. Target: BMI

b. This coefficient is set to zero because it is redundant.

Suppl.Table 38. *Correlations of Fixed Coefficients[a] of a Generalized Linear Mixed Model to evaluate the coefficients estimates of AHI, RE, ATED and RANIgE on BMI as a target variable.*

	Intercept	AHI	RE	RANIgE= 0	RANIgE= 1	ATED=0	ATED=1
Intercept	1,000	1,000	-1,000	-1,000	.[b]	1,000	.[b]
AHI	1,000	1,000	-1,000	-1,000	.[b]	1,000	.[b]
RE	-1,000	-1,000	1,000	1,000	.[b]	-1,000	.[b]
RANIgE= 0	-1,000	-1,000	1,000	1,000	.[b]	-1,000	.[b]
RANIgE= 1	.[b]	.[b]	.[b]	.[b]	.[b]	.[b]	.[b]
ATED=0	1,000	1,000	-1,000	-1,000	.[b]	1,000	.[b]
ATED=1	.[b]	.[b]	.[b]	.[b]	.[b]	.[b]	.[b]

Probability distribution: Gamma

Link function: Log

a. Target: BMI

b. One or both coefficients are redundant.

Suppl.Table 39. *Random Effect of ATED in a Generalized Linear Mixed Model to evaluate the coefficients estimates of AHI, RE, ATED and RANIgE on BMI as a target variable.*
Block 1

Random Effect	
Block	Intercept
Intercept	,016

Covariance Structure: Variance components
Subject Specification: ATED

Suppl.Table 40. *Covariance Parameters Summary in a Generalized Linear Mixed Model to evaluate the coefficients estimates of AHI, RE, ATED and RANIgE on BMI as a target variable.*

Covariance Parameters	Residual Effect	1
	Random Effects	1
Design Matrix Columns	Fixed Effects	7
	Random Effects	1[a]
Common Subjects		2

Common subjects are based on the subject specifications for the residual and random effects and are used to chunk the data for better performance.

a. This is the number of columns per common subject.

Suppl. Table 41. *Residual Effect in a Generalized Linear Mixed Model to evaluate the coefficients estimates of AHI, RE, ATED and RANIgE on BMI as a target variable.*

Estimate	Std. Error	Z
,029	,008	3,674

Covariance Structure: Scaled Identity
Subject Specification: (None)

Suppl.Table 42. *Random Effect in a Generalized Linear Mixed Model to evaluate the coefficients estimates of AHI, RE, ATED and RANIgE on BMI as a target variable.*

Random Effect Covariance	Estimate	Std. Error	Z	Sig.	95% Confidence Interval Lower	95% Confidence Interval Upper
Var(Intercept)	,016[a]	.	.	.	.	.

Covariance Structure: Variance components

Subject Specification: ATED

a. This parameter is redundant.

Suppl. Table 43. *Estimated Means of RANIgE/No RANIgE in a Generalized Linear Mixed Model to evaluate the coefficients estimates of AHI, RE, ATED and RANIgE on BMI as a target variable.*

			95% Confidence Interval	
RANIgE	Mean	Std. Error	Lower	Upper
NoRANIgE	2,831	,013	2,805	2,858
RANIgE	2,915	,025	2,864	2,965

Continuous predictors are fixed at the following values: AHI=4,4781,RE=20,825

Suppl. Table 44. *Estimated Means of No ATED/ATED in a Generalized Linear Mixed Model to evaluate the coefficients estimates of AHI, RE, ATED and RANIgE on BMI as a target variable.*

			95% Confidence Interval	
ATED	Mean	Std. Error	Lower	Upper
No Asthma Treatment or Eviction Diet	2,890	,006	2,877	2,903
Asthma Treatment or Eviction Diet	2,856	,005	2,845	2,866

Continuous predictors are fixed at the following values: AHI=4,4781,RE=20,825

Path analysis (Suppl.Tables 45-51)

Suppl Table 45. Mediation analysis was performed to assess the mediating role of RE and AHI in the relationship between RANIgE / AT or ED and BMI.

Statistics. The minimum values, maximum values, mean values, standard deviations, skewness, and kurtosis coefficients of RANIgE, AT or ED, RE, AHI, BMI are reported.

Variable	Min	Max	Mean	St.Dev	Skewness	c.r.	**kurtosis**	**c.r.**
RANIgE	,000	1,000	,34	,483	,658	1,519	-1,567	-1,810
AT or ED	,000	1,000	,41	,499	,382	,882	-1,854	-2,141
Respiratory Effort	1,100	74,900	20,825	15,761	1,365	3,153	2,435	2,811
AHI	,100	18,800	4,4781	3,88	1,933	4,465	4,522	5,221
BMI	12,800	32,000	17,681	4,28	1,922	4,438	3,469	4,006
Multivariate							6,946	2,348

Suppl Table 46. Mediation analysis was performed to assess the mediating role of RE and AHI in the relationship between RANIgE / AT or ED and BMI.

Correlations of RANIgE, AT or ED, RE, AHI, BMI are reported

		RANIgE	AT or ED	RE	AHI	BMI
RANIgE	Pearson Correlation	1	-,063	,483**	,342	,440*
	Sig. (2-tailed)		,733	,005	,056	,012
	N	32	32	32	32	32
AT or ED	Pearson Correlation	-,063	1	-,407*	-,320	-,310
	Sig. (2-tailed)	,733		,021	,074	,084
	N	32	32	32	32	32
RespiratoryEffort	Pearson Correlation	,483**	-,407*	1	,349	,530**
	Sig. (2-tailed)	,005	,021		,050	,002
	N	32	32	32	32	32
AHI	Pearson Correlation	,342	-,320	,349	1	,567**
	Sig. (2-tailed)	,056	,074	,050		<,001
	N	32	32	32	32	32
BMI	Pearson Correlation	,440*	-,310	,530**	,567**	1
	Sig. (2-tailed)	,012	,084	,002	<,001	
	N	32	32	32	32	32

**. Correlation is significant at the 0.01 level (2-tailed).

*. Correlation is significant at the 0.05 level (2-tailed).

Suppl Table 47. A mediation analysis was performed to assess the mediating role of RE and AHI in the relationship between RANIgE / AT and ED. Collinearity Statistics of RANIgE, AT or ED, AHI, RE were reported.

		Collinearity Statistics	
Model		Tolerance	VIF
1	(Constant)		
	RespiratoryEffort	,539	1,856
	AHI	,825	1,212
	AT or ED	,694	1,442
	RANIgE	,699	1,431

**Suppl.Table 48. Regression Weights**			**Estimate**	**S.E.**	**c.r.**	***p***	**Label**
AHI	<---	AT or ED	-2.332	1.247	-1.870	**.061**	PATAEAHI
AHI	<---	RANIgE	2.597	1.289	2.014	**.044**	PRAHI
Respiratory Effort	<---	RANIgE	15.007	4.645	3.231	**.001**	Pranre
Respiratory Effort	<---	AT or ED	-11.931	4.492	-2.656	**.008**	Patedre
BMI	<---	AT or ED	-.477	1.308	-.365	.715	Patedbmi
BMI	<---	AHI	.432	.163	2.655	**.008**	Pahibmi
BMI	<---	RANIgE	1.431	1.415	1.011	.312	pRANBMI
BMI	<---	Respiratory Effort	.080	.045	1.766	**.077**	pREBMI

Suppl Table 49. Squared Multiple Correlations.

Path analysis with serial mediation in AMOS.

Variable	Estimate	Lower	Upper	*p*
RespiratoryEffort	,453	,236	,577	**,003**
AHI	,162	,011	,356	**,001**
BMI	,453	,104	,687	**,003**

Note. Standard errors and significance testing for the indirect effects were estimated using the bootstapping method in Amos.

	Total effects				Direct Effect				Indirect effects							
Hypothesis	B	SE	T	P	B	SE	T	P	Hypothesis	B	SE	T	P	Percentile bootstrap 95% CI		Result
														L	U	
RANIgE→ BMI	3.749	1.76	2.14	**.022**	1.431	1.48	0.96	.306	RANIgE→(RE+AHI) →BMI	2.318	1.688	1.37	.155	-.234	5.22	
RANIgE→ RE	15.007	5.48	2.73	**.002**	15.007	5.48	2.73	**.002**								
RE→ BMI	.080	.065	1.33	.326	.080	.065	1.23	.326								
RANIgE→ AHI	2.597	1.62	1.60	**.082**	2.597	1.62	1.60	**.082**								
AHI→ BMI	.432	.307	1.40	.295	.432	.307	1.40	.295								

AT or AE→BMI	*-2.435*	*1.13*	*-2.15*	***.021***	*-.477*	*1.25*	*-.381*	.605	AT or ED→(RE+AHI)→BMI	-1.958	1.361	-1.43	.212	-3.982	.436
AT or AE→RE	*-11.931*	*4.36*	*-2.73*	***.006***	*-11.93*	*4.36*	*-2.73*	**.006**							
AT or AE→AHI	*-2.332*	*1.10*	*-2.12*	***.025***	*-2.332*	*1.10*	*-2.12*	**.025**							

Note. SE: Standard Error, RANIgE: Respiratory and Non-IgE mediated Allergies, RE: Respiratory Effort, AHI: Apnoea Hypopnea Index, BMI: Body Mass Index, AT or AE: Asthma treatment or eviction diet, β=Estimate, CI: Confidence Intervals, L: Lower, U: Upper, Bootstrap 5000

Suppl Table 50. Unstandardized total, direct and indirect effects through path analysis with serial mediation in AMOS.

Suppl Table 51. User-defined estimands. Path analysis with serial mediation in AMOS.

Variable	Estimate	Lower	Upper	P
RANIgEtoAHItoBMI	1,122	-,219	3,228	,251
RANIgEtoREtoBMI	1,197	-,531	3,444	,325
ATAEtoAHItoBMI	-1,007	-2,446	,414	,295
ATAEtoREtoBMI	-,951	-2,477	,504	,328
RANIgEtoAHI	2,597	,104	5,415	**,082**
RANIgEtoRE	15,007	6,645	24,329	**,002**
ATAEtoAHI	-2,332	-4,150	-,556	**,025**
ATAEtoRE	-11,931	-18,840	-4,752	**,006**
ATAEtoBMI	-,477	-2,634	1,431	,605
REtoBMI	,080	-,051	,161	,326
AHItoBMI	,432	-,340	,690	,295
RANIgEtoBMI	1,431	-1,006	3,821	,306
